# The Regulatory Factor ZFHX3 Modifies Circadian Function in SCN via an AT Motif-Driven Axis

**DOI:** 10.1016/j.cell.2015.06.060

**Published:** 2015-07-30

**Authors:** Michael J. Parsons, Marco Brancaccio, Siddharth Sethi, Elizabeth S. Maywood, Rahul Satija, Jessica K. Edwards, Aarti Jagannath, Yvonne Couch, Mattéa J. Finelli, Nicola J. Smyllie, Christopher Esapa, Rachel Butler, Alun R. Barnard, Johanna E. Chesham, Shoko Saito, Greg Joynson, Sara Wells, Russell G. Foster, Peter L. Oliver, Michelle M. Simon, Ann-Marie Mallon, Michael H. Hastings, Patrick M. Nolan

**Affiliations:** 1 MRC Harwell, Harwell Science and Innovation Campus, Oxfordshire OX11 0RD, UK; 2 MRC Laboratory of Molecular Biology, Cambridge Biomedical Campus, Cambridge CB2 0QH, UK; 3 New York Genome Center, 101 Avenue of the Americas, New York, NY 10013, USA; 4 Department of Biology, New York University, New York, NY 10012, USA; 5 Nuffield Department of Clinical Neurosciences, University of Oxford, Oxford OX3 9DU, UK; 6 Acute Stroke Program, Radcliffe Department of Clinical Medicine, University of Oxford, Oxford OX3 9DU, UK; 7 MRC Functional Genomics Unit, Department of Physiology, Anatomy and Genetics, University of Oxford, Parks Road, Oxford OX1 3PT, UK; 8 Department of Genetics, Erasmus University Medical Center, 3000 CA Rotterdam, the Netherlands; 9 Faculty of Medicine, University of Tsukuba, 1-1-1 Tennodai, Tsukuba 305-8575, Japan

## Abstract

We identified a dominant missense mutation in the SCN transcription factor *Zfhx3*, termed short circuit (*Zfhx3*^*Sci*^), which accelerates circadian locomotor rhythms in mice. ZFHX3 regulates transcription via direct interaction with predicted AT motifs in target genes. The mutant protein has a decreased ability to activate consensus AT motifs in vitro. Using RNA sequencing, we found minimal effects on core clock genes in *Zfhx3*^*Sci/+*^ SCN, whereas the expression of neuropeptides critical for SCN intercellular signaling was significantly disturbed. Moreover, mutant ZFHX3 had a decreased ability to activate AT motifs in the promoters of these neuropeptide genes. Lentiviral transduction of SCN slices showed that the ZFHX3-mediated activation of AT motifs is circadian, with decreased amplitude and robustness of these oscillations in *Zfhx3*^*Sci/+*^ SCN slices. In conclusion, by cloning *Zfhx3*^*Sci*^, we have uncovered a circadian transcriptional axis that determines the period and robustness of behavioral and SCN molecular rhythms.

## Highlights

• *Zfhx3* missense mutation underlies the short circuit (*Zfhx3*^*Sci*^) circadian phenotype
• *Zfhx3*^*Sci*^ reduces the ability of ZFHX3 to activate transcription via AT motifs
• *Zfhx3*^*Sci*^ phenotype is associated with decreased activation of AT motif in neuropeptide promoters
• Circadian activation in SCN reveals AT motif as a new clock-regulated transcriptional axis

## Graphical Abstract

**Figure:**
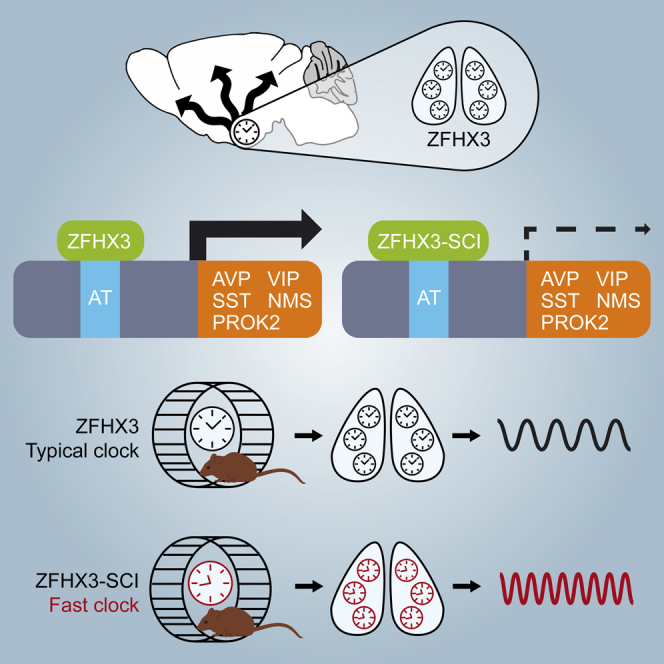

## Teaser

A transcription factor expressed in discrete adult hypothalamic nuclei, including the suprachiasmatic nucleus, regulates circadian locomotor rhythms in vivo through the expression of distinct neuropeptidergic genes to ensure robust synchronous oscillations and circadian rhythms.

## Introduction

Circadian rhythms are daily cycles with periods of ∼24 hr that affect multiple distinct physiological and behavioral outputs. Temporal regulation of these biological processes is managed, in part, by changes to the 24-hr transcriptional profile within a tissue (Koike et al., 2012). This transcriptional landscape is governed by a number of processes, including modification of DNA structure (Feng et al., 2011), and by the interaction of transcription factors with DNA sequence motifs in gene promoters (Bozek et al., 2009). Some motifs involved in circadian regulation, including E-box, D-box, and Rev-responsive elements (RREs) (Bozek et al., 2009, 2010; Ukai and Ueda, 2010), have been well characterized but have only a limited ability to explain the extensive influence of the core clockwork on circadian patterns of gene expression. Therefore, the discovery and characterization of additional motifs promises to reveal further levels of circadian regulatory complexity and facilitate a system-wide appreciation of the principles that underpin the temporal orchestration of gene expression.

In mammals, the current canonical model of the core molecular clock consists of a well-characterized transcriptional-translational feedback loop (TTFL), where CLOCK and BMAL1 drive the expression of E-box-regulated genes, including *Per* and *Cry*, whose protein products, in turn, negatively regulate these same genes by interacting with the CLOCK:BMAL1 complex (Zhang and Kay, 2010). Most cells contain such molecular clocks (Albrecht, 2012) synchronized to each other and to the external environment by signals derived from the principal circadian pacemaker, the suprachiasmatic nucleus (SCN) (Ralph et al., 1990). Within the SCN, the TTFL drives the molecular clock in individual cells, while at the tissue level, these individual SCN cells are synchronized through intercellular coupling to form a coherent oscillator (Aton and Herzog, 2005, Maywood et al., 2006). This coupling is driven by several neuropeptides, including vasoactive intestinal peptide (VIP) (Aton et al., 2005; Maywood et al., 2011; Brown et al., 2005) and gastrin-releasing peptide (GRP) (Albers et al., 1995; Brown et al., 2005). Together, they synchronize the intracellular molecular clockworks of individual neurons while also conferring robustness against perturbations (Liu et al., 2007). Moreover, these and additional peptides, including neuromedin S (NMS) and prokineticin 2 (PROK2), are important in conveying circadian signals from the SCN to the rest of the brain and periphery (Li et al., 2006; Prosser et al., 2007; Lee et al., 2015).

Insight into the molecular basis of circadian rhythms in mammals has advanced through gene discovery with the use of numerous approaches, including *N*-ethyl-*N*-nitrosourea (ENU) (Bacon et al., 2004). Discoveries of mammalian circadian genes, including *Clock* (Vitaterna et al., 1994) and *Fbxl3* (Godinho et al., 2007), attest to its utility. Uniquely, ENU-induced mutations can generate not only null alleles but also partial loss-of-function (hypomorphic) and dominant-negative alleles (Acevedo-Arozena et al., 2008). Consequently, ENU mutants have often revealed phenotypes that are novel, different, or more robust than the corresponding knockout mutant, including the dominant-negative *Clock*^Δ19^ mutant (Vitaterna et al., 1994; DeBruyne et al., 2006). Moreover, null mutations in genes with fundamental developmental roles are often embryonic lethal, while corresponding ENU mutants often survive to a later stage than that of the earliest essential function of the gene, thereby enabling the investigation of relevant phenotypes in adults (Acevedo-Arozena et al., 2008).

Here, we investigate the molecular mechanisms underlying the dominant short-circadian-period phenotype in the ENU mutant, short circuit, *Sci* (MGI: 2679554) (Bacon et al., 2004). Through positional candidate analysis, we establish that the *Sci* phenotype is caused by a mutation in the SCN transcription factor *Zfhx3*. This gene acts through transcriptional regulation of genes containing AT motifs in their promoter regions (Yasuda et al., 1994). We found that ZFHX3^Sci^ has a decreased ability to activate a consensus AT motif in vitro. Moreover, using lentiviral transduction of SCN slice cultures, we discovered that activation of AT motifs is under circadian control in SCN and is compromised by the *Zfhx3*^*Sci*^ mutation. We further found that, among the transcriptional consequences in adult *Zfhx3*^*Sci/+*^ SCN, expression of a number of neuropeptides critical for intercellular signaling was decreased. Using chromatin immunoprecipitation (ChIP), we found that ZFHX3 directly interacts with the AT motif in certain neuropeptide promoters. Importantly, we determined that the effect of the mutation on circadian period was associated with its diminished ability to regulate the transcription of these genes in adult animals. Pharmacological slowing of the TTFL caused a corresponding lengthening of the period of AT activation, suggesting that the ZFHX3/AT axis is sensitive to the core circadian loop. Furthermore, we confirmed that *Sci* is a gain-of-function mutation, as knockdown of *Zfhx3* in vivo and ex vivo can significantly lengthen behavioral and SCN molecular rhythms. In summary, circadian transcription driven by AT motifs is evident in adult SCN and represents a circadian gene-regulatory axis, extending beyond the well-characterized TTFL.

## Results

### Short Circuit, a Dominant Circadian Mutation in *Zfhx3*

**Figure 1:**
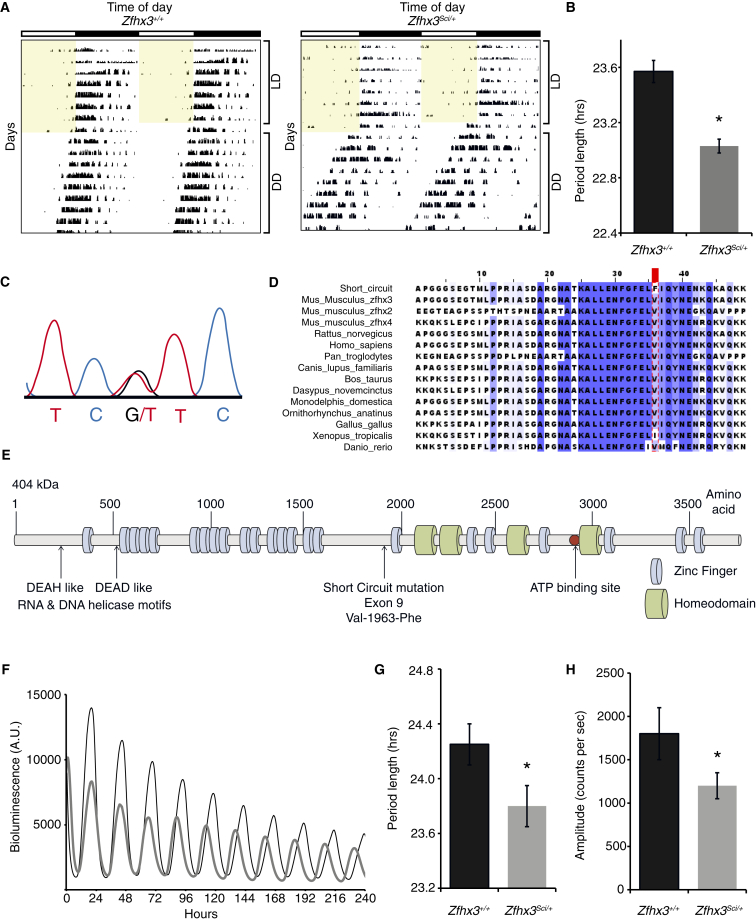
The Short Circuit (*Sci*) Phenotype Results from a Mutation in the Transcription Factor *Zfhx3* (A) Representative double-plotted actograms of wheel-running activity in *Zfhx3*^*Sci/+*^ and *Zfhx3*^*+/+*^ mice (7 days on a 12-hr light:dark (LD) schedule, followed by 2 weeks in constant darkness). Yellow shading represents periods when lights are on. Vertical black bars represent wheel running activity. (B) *Zfhx3*^*Sci/+*^ mice have a shorter free-running period than littermate controls in constant darkness (n = 6). ^∗^p = 0.0009. (C) The *Zfhx3*^*Sci*^ mutation mapped to the zinc-finger homeobox 3 (*Zfhx3*) locus and results in a G → T transversion at position 6620. (D) Multiple protein sequence alignment of ZFHX3 protein and its paralogues. The *Zfhx3*^*Sci*^ mutation, a V1963F substitution, is in a highly conserved region. (E) A schematic of the functional domains of ZFHX3; the *Zfhx3*^*Sci*^ mutation lies upstream of a zinc-finger domain. (F) Representative plots showing circadian activation of PER2::LUC expression in ex vivo SCN organotypic slices from *Zfhx3*^*Sci/+*^ (gray line) or *Zfhx3*^*+/+*^ (black line) animals. (G and H) The mean (G) period and (H) amplitude of PER2::LUC expression were decreased in *Zfhx3*^*Sci/+*^ (gray bars, n = 29) compared to *Zfhx3*^*+/+*^ (black bars, n = 21). ^∗^p < 0.05, t test. Error bars indicate SEM. See also Figures S1 and S2.

We conducted an ENU screen to uncover genetic factors affecting mammalian circadian behavior (Bacon et al., 2004). Among G1 animals in a dominant screen, we identified a mouse with a circadian period (τ_DD_; behavioral circadian period of the animals in constant darkness) shorter than the population mean (23.6 ± 0.08 hr; mean ± SEM). This phenotype was inherited in a dominant fashion, with a τ_DD_ (23.0 ± 0.05 hr) ranging from 21.4 to 23 hr (Figures 1A and 1B). We named the mutation short circuit (*Sci*) and mapped the dominant phenotype to mouse chromosome 8 between D8Mit138 (107.67 Mb) and D8Mit213 (110.57 Mb), containing 25 annotated genes. Among the candidates, zinc-finger homeobox 3 (*Zhfx3*) was highly and almost exclusively expressed in adult SCN (Lein et al., 2007). We identified a point mutation in exon 9, resulting in a G → T transversion at position 6620 (*Zfhx3* transcript, ENSMUST00000043896) (Figure 1C). The *Sci* mutation substitutes a phenylalanine for a valine at residue 1963 (V1963F) in a highly conserved region just upstream of the 17th zinc-finger motif (Figures 1D and 1E). Following the identification of the causative mutation, genotype and phenotype correlation demonstrated that the mutation causes homozygous lethality during embryonic development; therefore, only *Zfhx3*^*Sci/+*^ adult animals could be assessed phenotypically.

**Figure S1:**
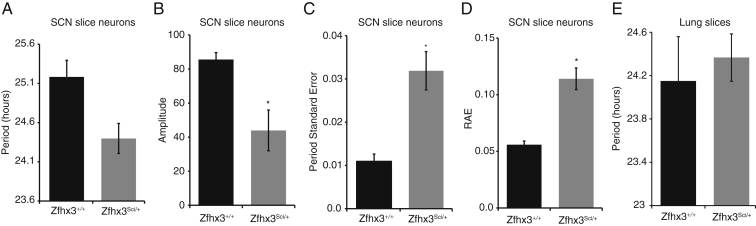
Disruption of PER2::LUC Rhythm in Individual Neurons In Ex Vivo SCN Slices, but Not in Ex Vivo Lung Slices, Related to Figure 1 (A–D) The (A) period and (B) amplitude of fusion protein expression was decreased in individual neurons from *Zfhx3*^*Sci/+*^ ex vivo SCN slices compared to similar wild-type neurons. Furthermore, the (C) period distribution was broader, and (D) RAE increased in the *Zfhx3*^*Sci/+*^ neurons (p < 0.05, t test). There were no differences in period in organotypic lung slices (Figure S1E), suggesting these differences may be specific to the central oscillator.

**Figure S2:**
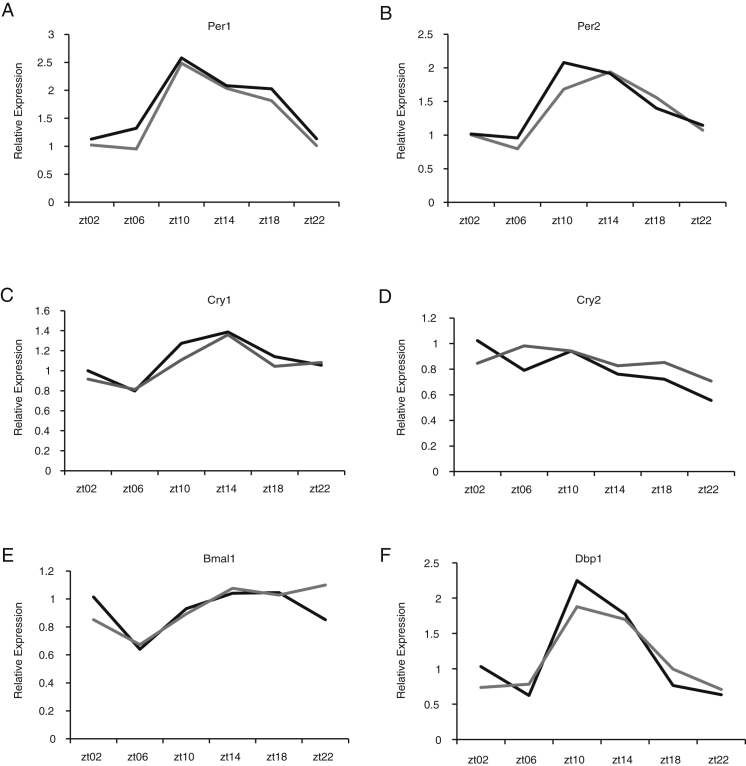
Circadian Gene Expression in *Zfhx3*^*Sci/+*^ and *Zfhx3*^*+/+*^ SCN across the Light:Dark Cycle, Related to Figure 1 (A–F) mRNA expression for (A) *Per1*, (B) *Per2*, (C) *Cry1*, (D) *Cry2*, (E) *Bmal1*, and (F) *Dbp1* showed no significant differences in the SCN of *Zfhx3*^*Sci/+*^ (gray lines) compared to *Zfhx3*^*+/+*^ (black lines) at multiple time points throughout the day (n = 4, p > 0.2, ANOVA).

Using ex vivo organotypic slices from *Zfhx3*^*Sci/+*^ or *Zfhx3*^*+/+*^ animals on a PER2::LUC background, we found that *Zfhx3*^*Sci/+*^ SCN had a shorter circadian period and a decreased amplitude of fusion protein expression relative to wild-type (p < 0.05, t test) (Figures 1F–1H). Similar decreases were found in individual neurons from *Zfhx3*^*Sci/+*^ SCN slices compared to wild-type neurons (Figures S1A and S1B). Furthermore, the period distribution was broader, and RAE increased in individual *Zfhx3*^*Sci/+*^ neurons imaged across the SCN circuit (Figures S1C and S1D) (p < 0.05, t test). There were no period differences in organotypic lung slices (Figure S1E), suggesting a central oscillator specific effect. These ex vivo SCN findings mirror the differences seen in locomotor behavior and suggest that photic inputs are not necessary for the expression of the short-period phenotype. Conversely, there were no significant differences in mRNA expression patterns of core circadian genes in the SCN of *Zfhx3*^*Sci/+*^ and *Zfhx3*^*+/+*^ animals sampled at six time points across the light:dark cycle (Figure S2), suggesting that any differences in clock gene expression are masked by the light:dark cycle. Collectively, these data predict that the mutation disrupts a *Zfhx3*-dependent and canonical TTFL-independent effect on SCN circadian period.

### Transcriptional Consequences in *Zfhx3*^*Sci/+*^ SCN

**Figure 2:**
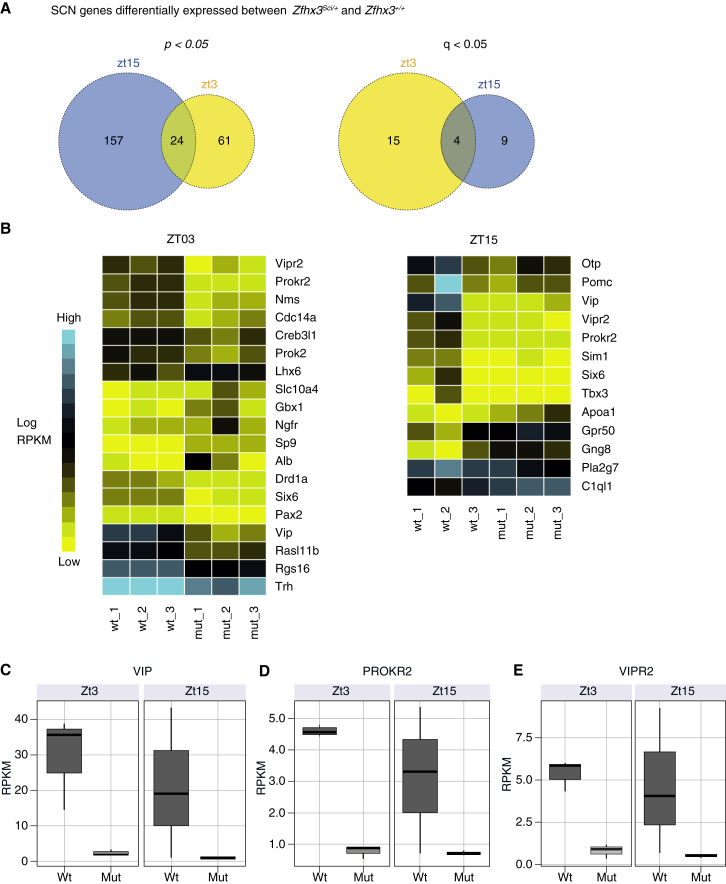
RNA Sequencing Reveals Transcripts Differentially Expressed in *Zfhx3*^*Sci/+*^ SCN (A) Venn diagrams depicting the number of differentially regulated transcripts, at ZT3 and ZT15, with a log_2_ fold change of >1 between *Zfhx3*^*Sci/+*^ and *Zfhx3*^*Sci/+*^ SCN. Genes were considered differentially expressed if they passed significance (left, p < 0.05; right, q < 0.05) for at least one of the three analysis methods (EdgeR, DESeq, or Cufflinks). (B) Transcripts with significant q values at each time point were used to populate heatmaps. For each time point, a heatmap shows the average expression in reads per kilobase per million (RPKM) for each transcript. wt, wild-type; mut, mutant. (C–E) Representative box plots of gene expression for (C) *Vip*, (D) *Prokr2*, and (E) *Vipr2* (q < 0.05). See also Tables S1 and S2.

We used RNA sequencing to identify transcriptional targets of ZFHX3. RNA was extracted from SCN tissue punches from *Zfhx3*^*Sci/+*^ and *Zfhx3*^*+/+*^ animals at zeitgeber time (ZT)3 and ZT15 (n = 3 for each time by genotype combination). RNA sequencing revealed that 242 genes were differentially expressed at either one or both time points (log_2_ fold change > 1, p < 0.05) (Table S1), with 28 of those surviving multiple testing correction (q < 0.05 in at least two quantification methods). At this more stringent level, 19 genes were differentially expressed at ZT3 and 13 at ZT15, while 4 genes were affected at both time points (Figure 2A). The majority of genes (17 of 28) showed a decrease in expression in *Zfhx3*^*Sci/+*^ (Figure 2B). Interestingly, the expression of a number of circadian-related neuropeptides was decreased in *Zfhx3*^*Sci/+*^ SCN, including *Vip*, its receptor (*Vipr2*), and prokineticin receptor 2 (*Prokr2*) (Figures 2C–2E). We conducted gene ontology (GO) enrichment analysis using all of the differentially expressed genes (q < 0.05 in at least one quantification method, n = 169) and found significant enrichment for a number of GO terms including neuron differentiation, regulation of cellular metabolic process, and regulation of cell proliferation (Table S2).

**Figure 3:**
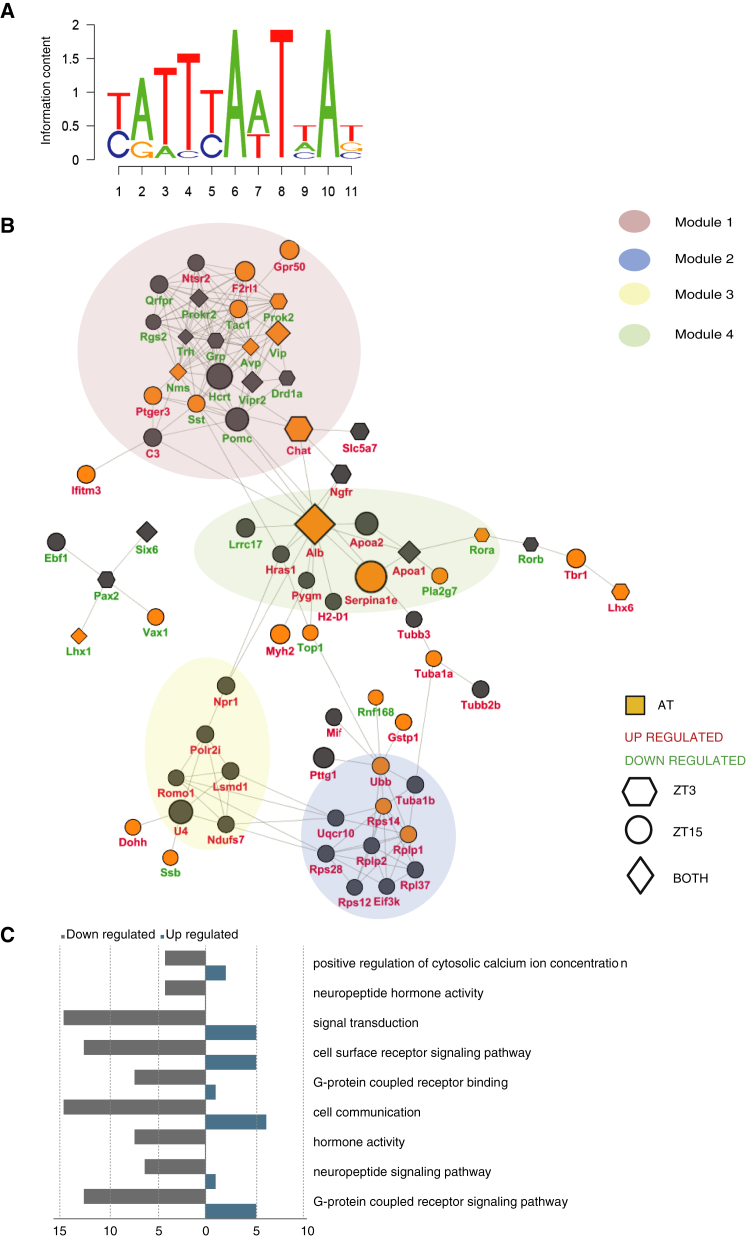
Functional Modules Determined for *Zfhx3*^*Sci/+*^ SCN Expression (A) The consensus AT motif derived from published AT sequences of genes regulated by *Zfhx3* (see Experimental Procedures). (B) A protein-protein interaction network representing the differentially expressed transcripts in *Zfhx3*^*Sci/+*^ SCN for all modules. Clustering analysis compartmentalized the network into modules of densely connected nodes. Positive identification of the consensus AT motif within the promoter regions of these transcripts is denoted by yellow shading. Node shape denotes the significance at different time points. Module 1 contains the greatest number of downregulated genes, AT motifs, and connectivity compared to other modules. (C) The top nine overrepresented GO terms for genes in module 1. The number downregulated (gray) and number upregulated (blue) are listed for each term. Neuropeptide and signaling activities are among the top functional roles of the genes in this module. See also Tables S3 and S4.

The limited work investigating this transcription factor to date has revealed a number of its gene targets (Berry et al., 2001; Mori et al., 2007; Qi et al., 2008; Yasuda et al., 1994). Using a phylogenetic shadowing-based approach (Bailey and Elkan, 1994), we constructed a motif-binding model for ZFHX3 based on the characterized binding motifs in these promoters (see Supplemental Experimental Procedures for more details) (Figure 3A). We then investigated whether this consensus AT motif, as well as the canonical circadian E-box, D-box, and RRE, were present in sequences upstream of the genes differentially regulated by ZFHX3 (Pscan score > 0.88). We found that the AT motif was present in promoter sequences from 39% of the differentially expressed genes, while the circadian motifs E-box, D-box, and RRE were absent (Figure 3B).

### Network Analysis of *Zfhx3*^*Sci/+*^*SCN* Gene Expression Reveals Functionally Distinct Modules

To investigate whether any biological pathways were specifically altered in *Zfhx3*^*Sci/+*^ SCN, we conducted further network analysis using the genes that were differentially expressed (q < 0.05 in at least one quantification method). More specifically, we used the Search Tool for the Retrieval of Interacting Genes/Proteins (STRING) to construct a protein interaction network. In order to characterize this network, we used a molecular complex detection algorithm (MCODE; Bader and Hogue, 2003). MCODE identified four modules, where module 1 achieved the highest ranked score (10.1, i.e., greatest connectivity), compared to module 2, which received a score of 5.5. Module 1 contained genes associated with neuropeptide function, whereas module 2 contained genes related to ribosome binding (Figure 3B), confirmed by GO analysis for both module 1 (Figure 3C; Table S3) and module 2 (Table S4).

### Downregulation of the Neuropeptide-Rich Module in *Zfhx3*^*Sci/+*^ SCN

**Figure 4:**
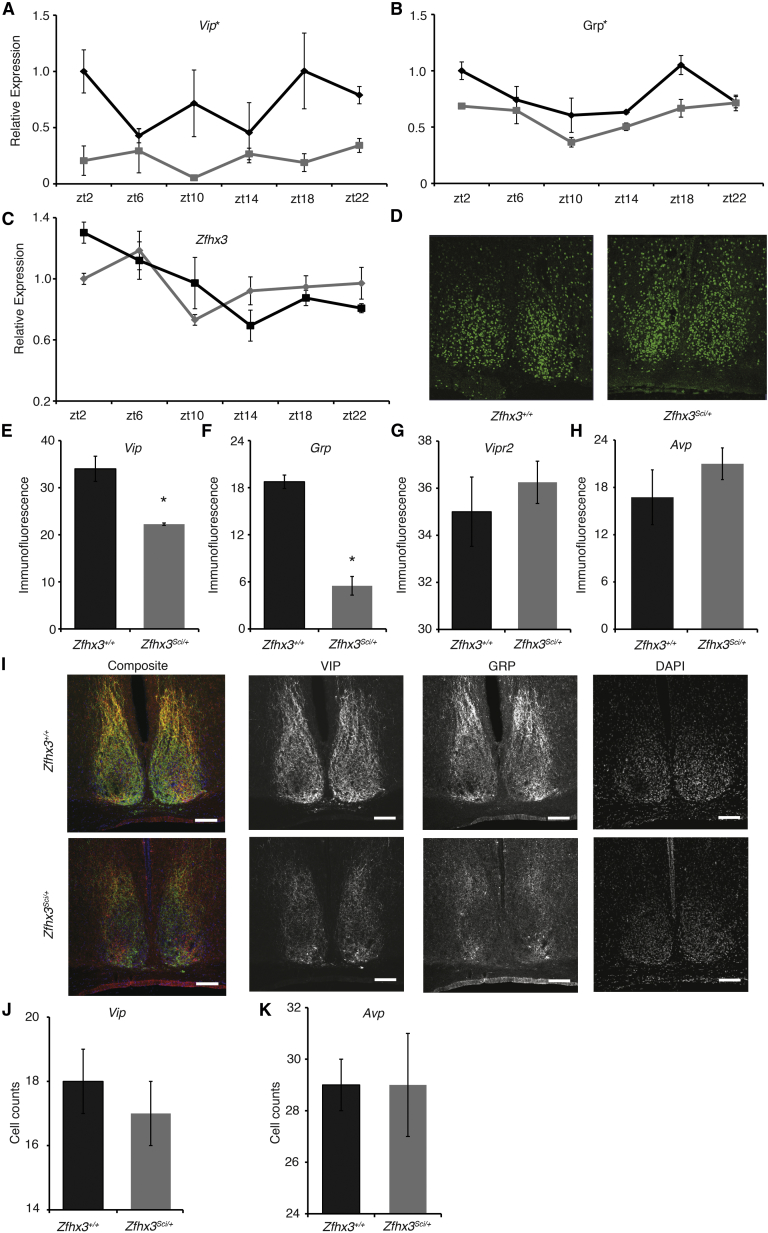
Significant Decreases in Neuropeptide Expression Detected in *Zfhx3*^*Sci/+*^ SCN (A and B) mRNA expression of both (A) *Vip* and (B) *Grp* was significantly decreased in SCN of *Zfhx3*^*Sci/+*^ (gray lines) compared to *Zfhx3*^*+/+*^ (black lines) at multiple time points (n = 4). p < 0.05, ANOVA. (C) *Zfhx3* mRNA expression in SCN is stable throughout the day and does not significantly differ by genotype (n = 4, gray and black lines represent *Zfhx3*^*Sci/+*^ and *Zfhx3*^*+/+*^ respectively). (D) ZFHX3 protein localization does not grossly differ across genotype at ZT6. (E and F) As shown here, (E) VIP and (F) GRP immunofluorescence in SCN was decreased in *Zfhx3*^*Sci/+*^ animals (p < 0.05). (G and H) As shown here, (G) VIPR2 and (H) AVP SCN protein expression was not different between genotypes (p > 0.1). (I) Confocal images (20× magnification) of immunostaining for VIP, GRP, and DAPI and the composite image in the SCN of *Zfhx3*^*+/+*^ (top panel) and *Zfhx3*^*Sci/+*^ (bottom panel). Scale bars, 100 μm. (J and K) The number of (J) VIP- and (K) AVP-immunopositive cells was quantified, with no significant differences detected across genotype. Error bars indicate SEM. ^∗^p < 0.05, t test. See also Figure S3.

A comparison of module 1 elements in *Zfhx3*^*Sci/+*^ and *Zfhx3*^*+/+*^ SCN revealed that 15 of 21 genes showed decreased expression in mutants. This included a number of neuropeptides and their receptors, such as: arginine vasopressin (*Avp*), gastrin-releasing peptide (*Grp*), *Nms*, prokineticin 2 (*Prok2*), *Prokr2*, *Vip*, and *Vipr2*. We measured their SCN mRNA expression at six time points across the light:dark cycle, using qPCR. *Grp*, *Vip*, and *Vipr2* had damped mRNA expression in *Zfhx3*^*Sci/+*^ SCN (two-way ANOVA, genotype main effect, p < 0.05). Expression of *Grp* and *Vip*, two neuropeptides previously shown to be important for regulating the firing patterns of SCN neurons (Aton et al., 2005; Brown et al., 2005; Maywood et al., 2006;), was decreased but not absent at most time points (Figures 4A and 4B). It should be noted that *Zfhx3* expression is not cyclic in the SCN, with no significant differences in mRNA expression (two-way ANOVA, time main effect, p > 0.2) and no gross differences in protein localization within the SCN between *Zfhx3*^*Sci/+*^ and *Zfhx3*^*+/+*^ animals (Figures 4C and 4D).

**Figure S3:**
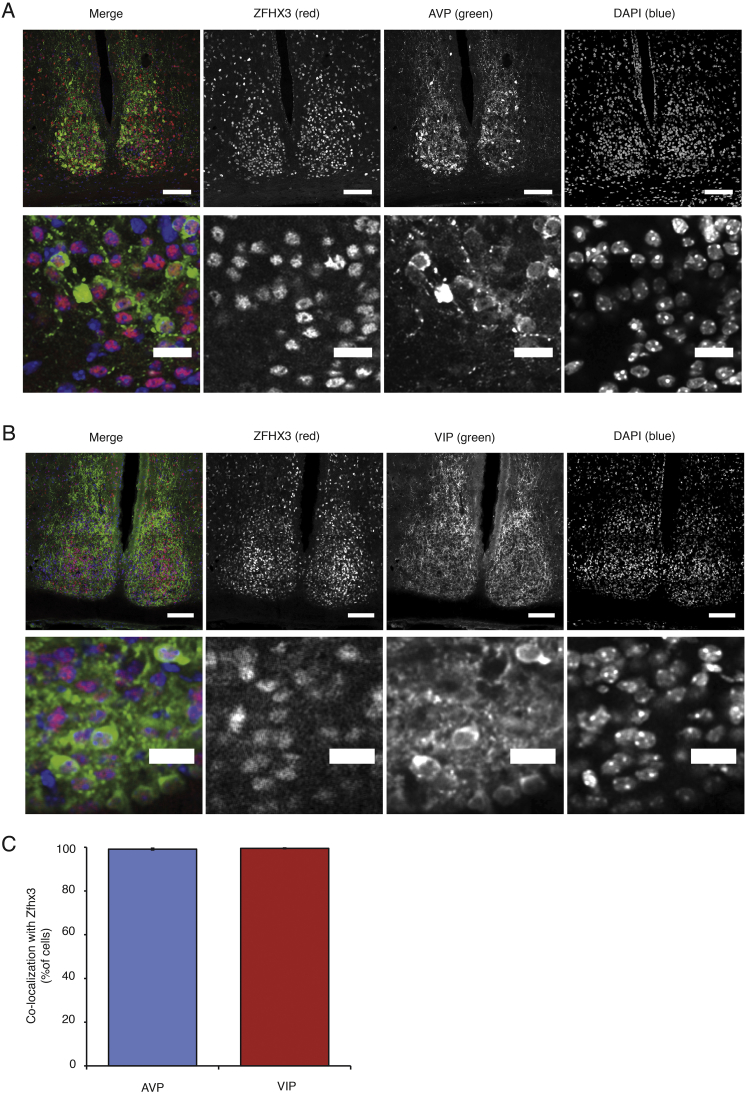
AVP and VIP Immunofluorescence Co-localizes with ZFHX3 Immunofluorescence, Related to Figure 4 (A and B) Confocal micrographs showing co-immunofluorescence of ZFHX3 and (A) AVP or (B) VIP within the SCN (scale bar, 100 μm) and at high magnification (scale bar, 20 μm) at ZT4. (C) Both AVP (blue bar) and VIP (red bar) neurons almost completely co-localized with ZFHX3 (n = 3 brains), graph shows mean ± SEM for the percentage of co-localized cells.

Using immunofluorescence, we compared AVP, GRP, VIP, and VIPR2 levels in *Zfhx3*^*Sci/+*^ and *Zfhx3*^*+/+*^ SCN. We found that GRP and VIP levels, but not AVP and VIPR2 levels, were significantly decreased in *Zfhx3*^*Sci/+*^ at ZT6 (Figures 4E–4I). There was a small but significant decrease in *Zfhx3*^*Sci/+*^ SCN size compared to *Zfhx3*^*+/+*^ (*Zfhx3*^*Sci/+*^ SCN size: 53,600 ± 2,400 μm^2^, n = 4; *Zfhx3*^*+/+*^ SCN size: 66,900 ± 1,600 μm^2^, n = 4; p < 0.05, t test, area of DAPI signal fluorescence). The data suggest that ZFHX3 actively influences the expression of these clock-relevant neuropeptides in the adult SCN and that this activation is disrupted in *Zfhx3*^*Sci/+*^ mice. These differences were not primarily due to aberrant terminal differentiation, as there was no significant reduction in the number of AVP or VIP immuno-positive neurons (Figures 4J and 4K). Interestingly, ZFHX3 immuno-positive neurons almost completely overlapped with those for both AVP and VIP (Figure S3).

### ZFHX3^Sci^ Has a Diminished Ability to Activate Transcription via the Circadian AT Motif

In order to investigate whether ZFHX3 might actively regulate module 1 genes in the adult SCN, we searched for strongly conserved consensus AT-motif, E-box, D-box, and RRE sequences within this module (Figure 3B). While none of the genes in the module had strongly conserved E-box, D-box, or RRE sequences, 7 of 15 genes showing damped oscillations in adult *Zfhx3*^*Sci/+*^ SCN had a predicted AT motif (motif conservation score ≥ 0.88) within 0.5 kb of the transcription start site (TSS). This finding suggests that the motif may play a specific role in regulating the neuropeptide network and reveals a mechanism in regulating neural rhythms within the SCN.

**Figure 5:**
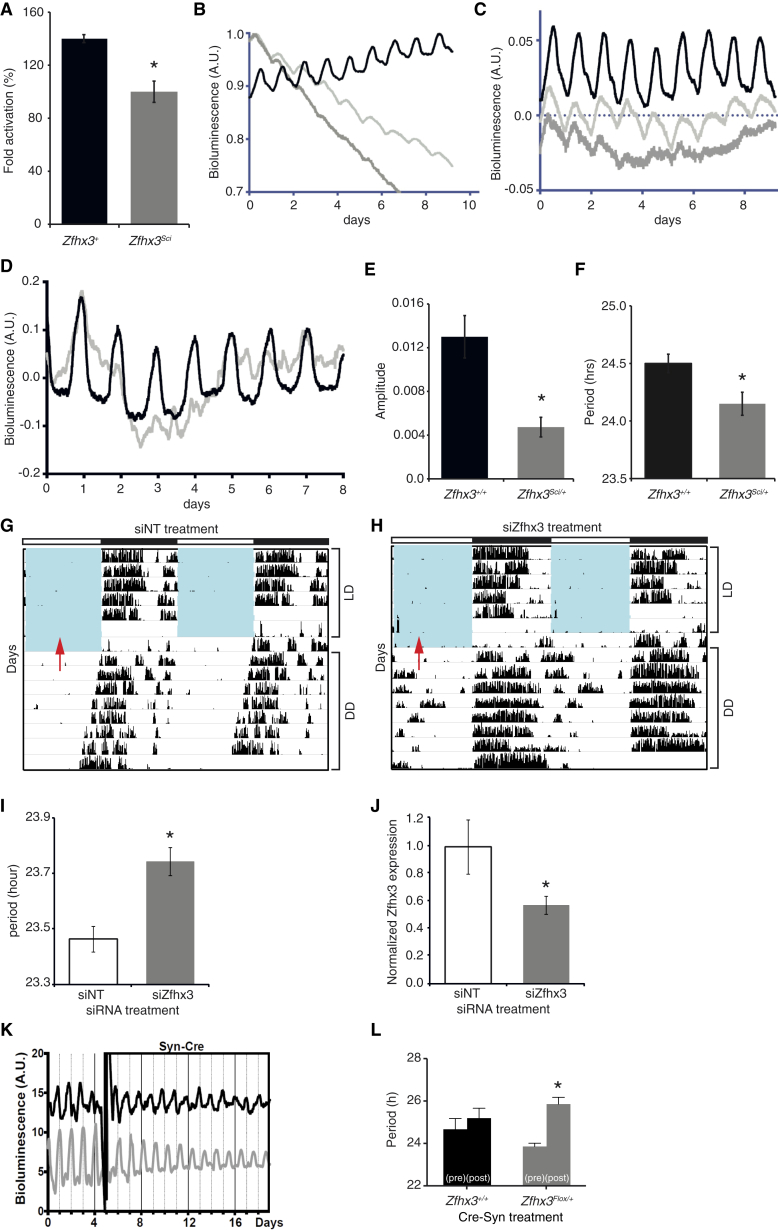
ZFHX3^SCI^ Differentially Activates a Circadian Motif in SCN (A) In vitro activation of the AT motif by overexpressing *Zfhx3* without and with the *Sci* mutation (*Zfhx3*^*+*^ and *Zfhx3*^*Sci*^ respectively) using a luciferase reporter construct driven by the AT motif (×7) in HEK293 cells. *Zfhx3*^*+*^ transcriptionally activated the AT motif, while *Zfhx3*^*Sci*^ did not (p < 0.05, t test). (B and C) As shown, (B) raw data and (C) de-trended data show circadian activation of AT sequences in SCN slices transduced by LVs coding for the luciferase reporter driven by the AT motif. A.U., arbitrary units. (D) AT-motif-driven luciferase expression in SCN slices from *Zfhx3*^*Sci/+*^ (gray lines) and *Zfhx3*^*+/+*^ (black lines) mice. (E and F) There was a substantial decrease in (E) the amplitude of AT motif activation in *Zfhx3*^*Sci/+*^ and a small, but significant decrease in (F) period compared to *Zfhx3*^*+/+*^ (p < 0.05, t test). (G and H) Representative double-plotted actograms of wheel-running activity in C57Bl/6 mice injected with (G) control siRNA (siNT) or (H) siZfhx3 (arrow denotes time of injection). Blue shading represents periods when lights are on. Vertical black bars represent wheel running activity. LD, light:dark cycle. (I) Animals injected with siZfhx3 had a significantly lengthened τ_DD_ (23.74 ± 0.05 hr, mean ± SEM) compared to control siRNA (23.46 ± 0.05 hr). p < 0.05, t test. (J) Injection of siZfhx3 led to a 43% downregulation of *Zfhx3* mRNA levels compared to control siRNA (p < 0.05, t test). (K and L) In (K), representative plots are shown of AT activation before and after transduction with the Syn-CRE vector in ex vivo SCN of *Zfhx3*^*+/+*^ (black lines) and *Zfhx3*^*Flox/+*^ mice (gray lines). (L) *Zfhx3* deletion in *Zfhx3*^*Flox/+*^ SCN significantly lengthened the period of AT activation relative to Zfhx3^+/+^. Error bars indicate SEM. ^∗^p < 0.05, t test. See also Figure S4.

**Figure S4:**
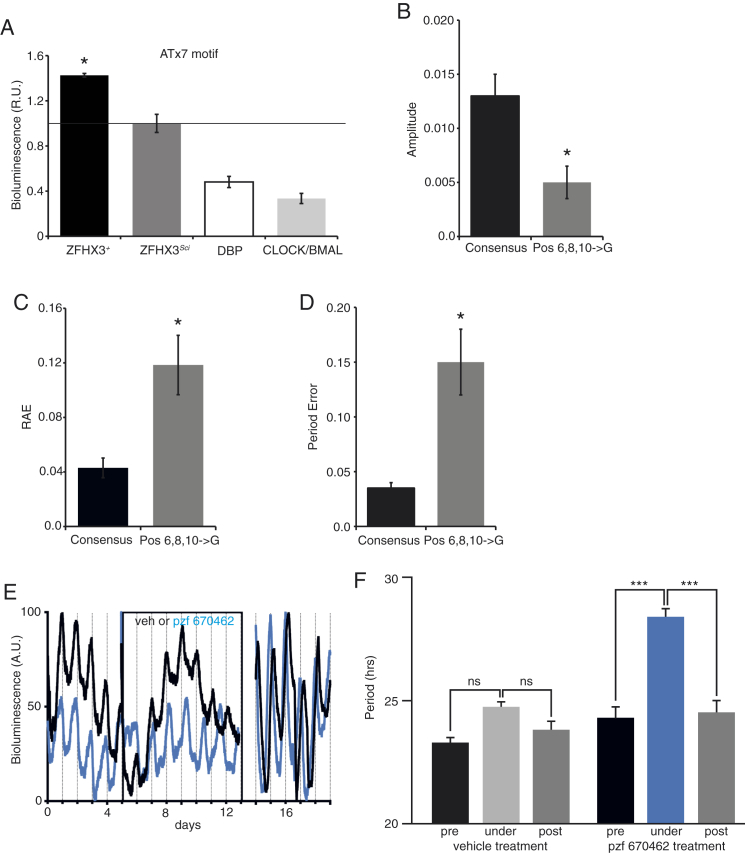
ZFHX3^+^ Preferentially Activates a Novel, Circadian Motif in SCN, Related to Figure 5 (A) In vitro activation of the AT motif by recombinant ZFHX3, DBP, or combined CLOCK and BMAL using a luciferase reporter construct driven by the AT motif (×7) in HEK293 cells. *Zfhx3*^*+*^ activation of the AT motif was more than 3 times that seen for either DBP or CLOCK/BMAL (p < 0.05, t test). Activation of AT sequences in SCN slices transduced by LVs showed cyclic activation in SCN slices from *Zfhx3*^*+/+*^ animals (Figures 5B and 5C). (B–D) Mutations of the three most conserved residues (positions 6, 8, 10) in the AT motifs strongly reduced AT activation rates (gray bars) when compared to the AT consensus (black bars). This effect was quantified by measuring both the (B) amplitude and robustness of these oscillations, (C) relative amplitude error, and (D) period error (p < 0.05, t test). (E) SCN slices expressing the AT-luciferase reporters were treated with pzf 670462 1 μM (blue lines) or vehicle (black lines), respectively. (F) Period of AT-mediated oscillations was significantly lengthened by pzf 670462 (p < 0.001, 2-way ANOVA repeated-measures), whereas it was unaffected by vehicle treatment. This effect was reversible upon removal of the drug.

To test whether the *Sci* mutation affects the ability of ZFHX3 to regulate transcription via the AT consensus motif, we cloned this motif (×7) into the pGL3-Enhancer Luciferase Reporter Vector. This reporter was then co-transfected with an expression vector containing recombinant *Zfhx3*, with or without the *Sci* mutation (*Zfhx3*^*Sci*^ and *Zfhx3*^*+*^, respectively), into HEK293 cells. We assayed transcriptional activity in cell lysates using the dual luciferase assay and found that *Zfhx3*^*+*^ showed an increased activation relative to the empty vector. In contrast, activation by *Zfhx3*^*Sci*^ was no different from that of the empty vector, supporting our hypothesis that the *Sci* mutation results in a decreased ability of ZFHX3 to activate transcription via the AT motif (p < 0.05, t test) (Figure 5A). We confirmed that the AT reporter response was specific to ZFHX3, as activation was at least three times higher than that of DBP or CLOCK/BMAL (p < 0.05, t test) (Figure S4A).

### AT Motif Reporter Oscillation Is Circadian and Is Damped in *Zfhx3*^*Sci/+*^ SCN

We explored whether similar deficits in AT-motif activation might underlie the *Zfhx3*^*Sci/+*^ phenotype in ex vivo SCN. To investigate this, we generated lentiviral vectors (LVs) encoding a luciferase reporter driven by the AT motif (×7) to transduce slices. Transcription from the AT motif was active and showed a distinct circadian oscillation in the SCN (period = 24.5 ± 0.1 hr; n = 5) (Figure 5B). To test the specificity of the AT motif, we mutated its three most conserved residues (residues 6, 8, and 10), and generated LV-driving luciferase expression from these mutated sequences. Amplitude and robustness (as assessed by relative amplitude error and period error) of the oscillations driven by these “defective” sequences in the SCN were strongly reduced (p < 0.05, t test) (Figures 5B and 5C; Figures S4B–S4D), consistent with the specificity of AT-motif-mediated transcription.

We then compared AT-motif-driven luciferase expression in SCN slices from *Zfhx3*^*+/+*^ and *Zfhx3*^*Sci/+*^ animals. We found a 65% decrease in amplitude of the circadian oscillation of the AT motif in *Zfhx3*^*Sci/+*^ SCN (p < 0.05, t test) (Figure 5D). Moreover, consistent with the amplitude and period shortening of PER2::LUC oscillations in *Zfhx3*^*Sci/+*^ SCN slices, both the amplitude and period of AT-motif-driven luciferase oscillations were reduced when compared to *Zfhx3*^*+/+*^ (period for *Zfhx3*^*Sci/+*^: 24.1 ± 0.1 hr; n = 8; period for *Zfhx3*^*+/+*^: 24.5 ± 0.1 hr; n = 8; p < 0.05, t test) (Figures 5E and 5F).

To determine whether the circadian oscillation of AT activation may be sensitive to the TTFL, we treated SCN with PF670462, an inhibitor of casein kinase 1 delta/epsilon that is known to slow down the TTFL by delaying degradation of PER proteins in the SCN (Meng et al., 2010). Before treatment, transduced SCN showed clear circadian oscillations of AT-driven bioluminescence (Figure S4E). On treatment with vehicle, period did not change, whereas on addition of PF670462, there was a significant period lengthening, which was completely reversible on washout (Figure S4F). Thus, the circadian oscillation of the ZFHX3/AT axis may be sensitive to the core TTFL and, in turn, acts as an output transducer of the circadian signal to its respective target genes.

### Ex Vivo or In Vivo Knockdown of *Zfhx3* SCN Expression Lengthens Circadian Period

To investigate the nature of the *Sci* mutation further and to establish a sustained regulatory role for *Zfhx3* in postnatal and adult SCN, we used two complementary approaches to knock down *Zfhx3*. In the first, we used in vivo RNAi to knock down in the adult SCN of C57Bl/6 mice. SiRNA was administered via stereotaxic injection into the third ventricle adjacent to the SCN to mice on a 12:12 light:dark cycle. One day after injection, mice were placed in constant darkness (DD) for 8 days to measure τ_DD_. Surprisingly, animals injected with siZfhx3 had a significantly lengthened τ_DD_ (23.74 ± 0.05 hr), compared to control siRNA-injected animals (23.46 ± 0.05 hr), p < 0.05, t test (Figures 5G–5I). Injection of siZfhx3 resulted in 43% downregulation of *Zfhx3* mRNA levels (p < 0.05, t test) (Figure 5J).

In a second experiment, we compared periods of AT-motif-driven molecular oscillations in ex vivo SCN slices using a previously described *Zfhx3*^*Flox*^ line (Sun et al., 2012). We compared AT-motif oscillations in *Zfhx3*^*+/+*^ and the *Zfhx3*^*Flox/+*^ SCN before and after transduction with Syn-CRE viral vectors. Treatment with Syn-CRE caused a 49% decrease in expression of the full-length *Zfhx3* transcript in the SCN *Zfhx3*^*Flox/+*^ mice (p < 0.05) and resulted in an ∼1.5-hr period lengthening (25.85 ± 0.37 hr versus 23.86 ± 0.20 hr, p < 0.05), while no lengthening was evident in controls (25.19 ± 0.47 hr versus 24.67 ± 0.51 hr) (Figures 5K and 5L), consistent with the in vivo siRNA experiment. Both in vivo and ex vivo findings confirm a sustained role for ZFHX3 in regulating circadian period in adult and postnatal SCN, and they additionally suggest that the *Sci* mutation has a dominant-negative, rather than a hypomorphic, effect on circadian period.

### ZFHX3 Directly Interacts with the AVP and VIP Promoters

**Figure 6:**
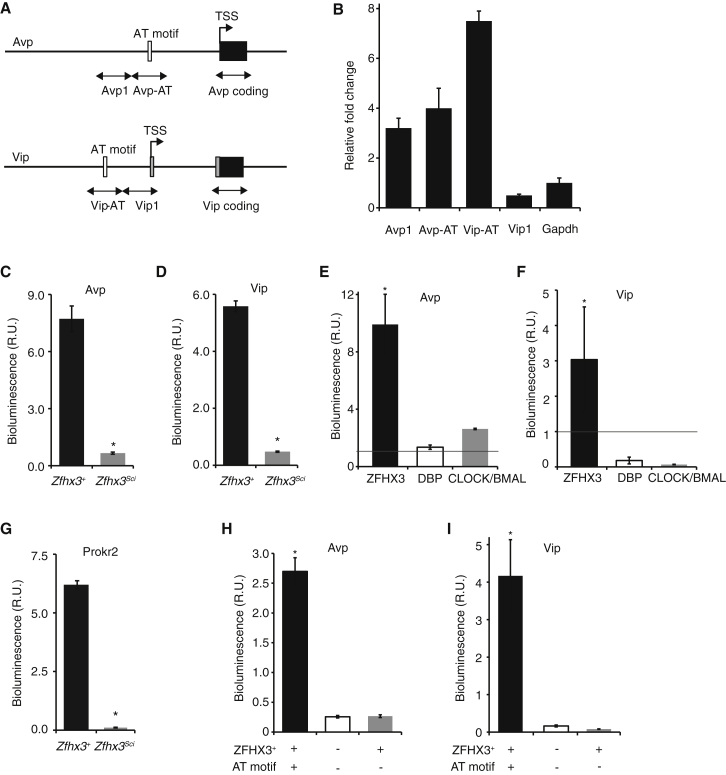
ZFHX3^SCI^ Interacts with and Differentially Activates the AT Motif in *Avp* and *Vip* Promoters Quantitative ChIP of *Zfhx3*^*+/+*^ SCN tissue samples using ZFHX3 antiserum. (A) Primer pairs were designed to span the AT motif (Avp-AT/Vip-AT) and an adjacent region (Avp-1/Vip-1) upstream of the TSS (primers in the coding region of each gene were used to normalize each reaction). (B) Zfhx3 binds to the promoter around the AT motif of both *Avp* and *Vip* compared to the control gene, *Gapdh*. Data are shown as fold change normalized for input relative to the corresponding coding region of each gene and are taken at ZT3 (n = 3). (C and D) Overexpression of ZFHX3 with the *Sci* mutation (ZFHX3^*Sci*^) was ineffective in activation of luciferase driven by AT-motif-containing promoters of (C) *Avp* and (D) *Vip* in HEK293 cells (p < 0.05, t test). R.U., relative units. (E and F) *Zfhx3*^*+*^ activation (black bars) of the (E) *Avp* and (F) *Vip* promoters was more than three times that seen for either DBP (white bars) or CLOCK/BMAL (gray bars) (p < 0.05, t test). (G) Overexpression ZFHX3^*Sci*^ was also ineffective in activation of the *Prokr2* promoter *Prokr2* in HEK293 cells (p < 0.05, t test). (H and I) Mutation of the three most conserved residues (positions 6, 8, and 10) in the AT motifs of both the (H) *Avp* and (I) *Vip* promoter constructs (gray bars) resulted in significantly decreased levels of ZFHX3+ activation compared to those with AT motifs intact (black bars) (p < 0.05, t test). White bars represent the activation of the mutated promoter constructs alone. Error bars indicate SEM. ^∗^p < 0.05, t test. See also Figure S5.

To confirm that ZFHX3 binds to the promoter of the key predicted target genes *Avp* and *Vip* in vivo, we performed quantitative ChIP on SCN tissue from *Zfhx3*^*+/+*^ animals (ZT3) using ZFHX3 antiserum. We found a significant increase in immunoprecipitated DNA upstream of the transcriptional start site (TSS) for both *Avp* and *Vip* compared to the *Gapdh* control promoter region, suggesting that ZFHX3 binds to both target gene promoters (Figures 6A and 6B). At the *Vip* promoter, this binding occurred in the vicinity of the AT motif to a far greater extent than in an adjacent upstream region. These data strongly suggest that ZFHX3 binds to *Avp* and *Vip* promoters in close proximity to AT consensus motifs.

### Failure to Activate Neuropeptide Promoter AT Motifs Underlies the *Zfhx3*^*Sci*^ Phenotype

**Figure S5:**
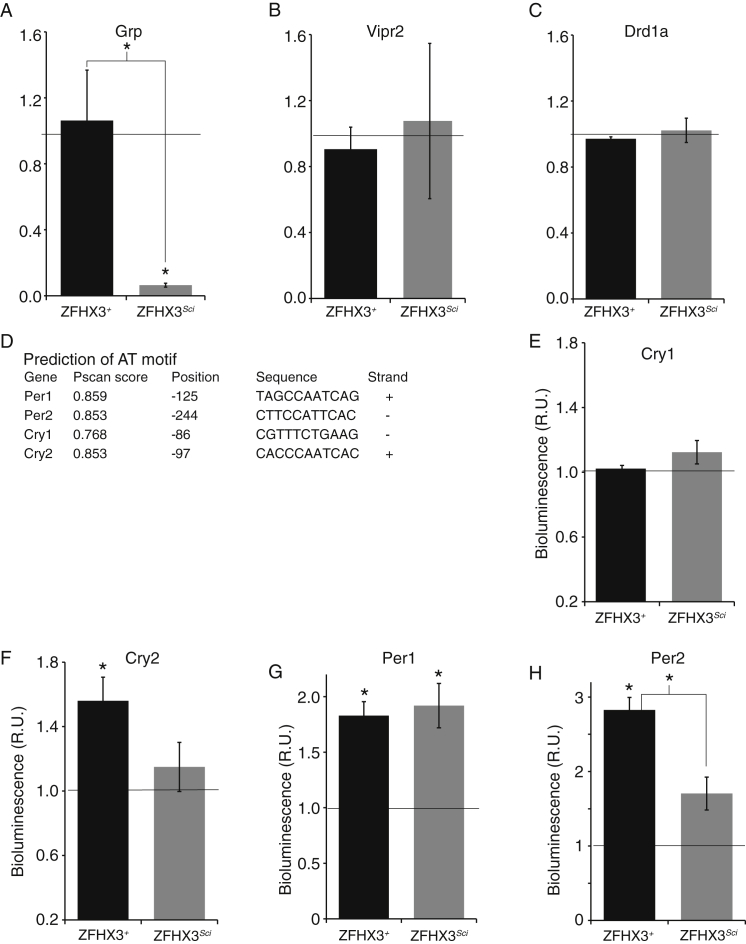
ZFHX3^SCI^ Interaction with the AT Motif in Circadian Gene Promoters, Related to Figure 6 (A–C) Overexpression of recombinant ZFHX3^Sci^ failed to activate the (A) *Grp*, (B) *Vipr2*, and (C) *Drd1a* promoter containing vectors (p > 0.05, t test, in HEK293 cells). (D) *Cry2*, *Per1*, and *Per2* have a predicted AT motif in their promoters, whereas *Cry1* does not (Pscan score and sequences shown; position is relative to start of first exon). (E) Co-transfection of ZFHX3^+^ failed to activate the *Cry1* promoter, while it did activate the (F) *Cry2*, (G) *Per1*, and (H) *Per2* promoters (p < 0.05, t test, in HEK293 cells). We found a small decrease in ZFHX3^Sci^ activation compared to ZFHX3^+^ for the *Per2* promoter (p < 0.05, t test, in HEK293 cells).

As several genes downregulated in module 1 had predicted AT motifs upstream of their TSSs (Figure 3B), we hypothesized that their diminished expression may be due to a decreased ability of ZFHX3^Sci^ to activate transcription via the AT motif. To test this, we cloned the module 1 gene putative promoters with strong predicted AT motifs (*Avp*, *Vip*; Pscan threshold > 0.85), with moderate predicted motifs (*Grp*, *Prokr2*; Pscan threshold > 0.80), or without one (*Drd1a*, *Vipr2*; Pscan threshold ≤0.8) into the pGL3-Enhancer Luciferase Reporter Vector. We co-transfected these reporters together with the *Zfhx3*^*Sci*^ or *Zfhx3*^*+*^ expression vectors into HEK293 cells. In both cases, there was increased transcriptional activation of reporters containing strong predicted AT motifs when we overexpressed ZFHX3^+^, while this activation was dramatically less when we overexpressed ZFHX3^Sci^ (Figures 6C and 6D). Furthermore, ZFHX3^+^-driven expression was significantly higher than that driven by DBP or by CLOCK/BMAL (Figures 6E and 6F). Co-transfection of reporters containing moderate predicted AT motifs with ZFHX3^+^ led to increased activation of the *Prokr2* reporter (Figure 6G; Figure S5A) but not the *Grp* reporter. Finally, we found no significant activation of those reporters without predicted AT motifs (Figures S5B and S5C).

To further investigate the role of the AT motif in ZFHX3-dependent transcription, we mutated the three most conserved residues (residues 6, 8, and 10) within the *Avp* and *Vip* promoter constructs. These constructs without AT motifs had significantly decreased levels of transcriptional activation (Figures 6H and 6I) (p < 0.05, t test). Our findings strongly support a role for ZFHX3-driven, AT-motif-mediated transcriptional activation in the maintenance and regulation of SCN neuropeptide expression. Furthermore, the short-period circadian phenotype evident both in vivo and ex vivo *Zfhx3*^*Sci/+*^ is likely mediated by a failure of ZFHX3^Sci^ to drive these oscillations effectively.

In addition to the neuropeptide genes identified in the RNA-sequencing analysis, circadian genes *Cry2*, *Per1*, and *Per2* have a predicted AT motif in their promoters, whereas *Cry1* does not (Figure S5D). Using a similar co-transfection strategy, we found a small but significant activation of the all three promoters with the predicted AT motif by ZFHX3^+^ but not for the *Cry1* promoter (Figures S5E–S5H). As all four of these promoters contain E-box sequences, these data suggest that the AT motif, not the E-box, mediates this ZFHX3^+^ activation.

## Discussion

The identification of circadian genes has advanced through the use of a number of approaches, including phylogenetic approaches, ENU mutagenesis screens, in silico analysis, and cell reporter screening methods. Identification of the *Zfhx3*^*Sci/+*^ mutant underlines the utility of mutagenesis approaches in discovering circadian phenotype/genotype associations in mammals (Vitaterna et al., 1994; Godinho et al., 2007). As with cell reporter screening methods, ENU mutagenesis has yielded molecular components that can modulate the cell-autonomous TTFL. However, as ENU mutagenesis screens assay in vivo phenotypes, they can uniquely detect mutants, such as *Sci*, that affect the SCN intercellular network. Furthermore, the ability of ENU to generate series of mutant alleles enables the discovery of different phenotypes from those detected by loss-of-function studies. This is evident in the opposing effects of *Zfhx3* siRNA knockdown and the *Zfhx3*^*Sci/+*^ mutant on τ_DD,_ suggesting that the *Zfhx3*^*Sci/+*^ mutant is not a hypomorphic allele and, instead, is a gain-of-function, possibly dominant-negative, allele. This highlights the continued value of using ENU mutagenesis, in conjunction with other methods, in circadian gene discovery.

We report that the short-period phenotype of *Sci* is caused by a mutation in *Zfhx3*, a gene highly expressed in adult SCN. We also found that the mutant protein ZFHX3^Sci^ has a decreased ability to transcriptionally activate an AT motif, a circadian motif present in the promoters of a class of neuropeptides, among other genes, that are critical for synchronous cellular rhythms of electrical firing and molecular cycling in the SCN (Maywood et al., 2006, 2011; Aton and Herzog, 2005). Furthermore, we show that activation of AT-dependent transcription follows a clear circadian cycle in the SCN, that the rhythm is sensitive to ZFHX3 function, and that it may also be downstream of the core TTFL. These results suggest a role for ZFHX3 beyond the core TTFL in modulating SCN free-running period in a cell-nonautonomous fashion, possibly by regulation of a neuropeptide network that governs the intercellular synchrony of the individual neuronal clocks in the adult SCN (Liu et al., 2007). We have provided evidence that ZFHX3 activates a clock-regulated transcriptional axis, thereby expanding the known chorus of SCN regulatory proteins responsible for the maintenance of mammalian circadian rhythms.

We provide evidence that a consensus AT motif can be transcriptionally activated in a circadian fashion in the SCN. Previous studies found that ZFHX3 can either positively (Qi et al., 2008) or negatively (Mori et al., 2007; Yasuda et al., 1994) regulate gene expression via AT motifs, depending on the functional domains and binding partners involved (Berry et al., 2001; Sakata et al., 2014). Our data show that ZFHX3 can positively regulate the expression of certain neuropeptides via a direct and AT-motif-dependent interaction with their promoters, and that this regulation is disrupted in *Zfhx3*^*Sci/+*^ mice. The ZFHX3-regulated AT motif now extends the list of DNA motifs managing the SCN circadian transcriptional landscape, thereby controlling circadian behavior (Bozek et al., 2009, 2010; Koike et al., 2012).

We have shown that the sequence specificity of the AT motif is critical for regulating the amplitude and robustness of its rhythmic activation. Furthermore, all measures were deficient in SCN slices from *Zfhx3*^*Sci/+*^ animals, supporting the case that ZFHX3 is a transregulator of this motif. It should be noted that, because *Zfhx3* mRNA expression does not strongly cycle within the SCN, there may be other circadian-regulated genes that act with ZFHX3 to activate the AT motif. This is not unprecedented, particularly within the SCN TTFL, as neither *Clock* mRNA nor its protein expression oscillate, but it still plays a key circadian role (Lee et al., 2001). Further exploration of ZFHX3 binding partners, particularly those that are circadian regulated, will contribute to our understanding of its role in adult SCN rhythmic oscillations.

**Figure 7:**
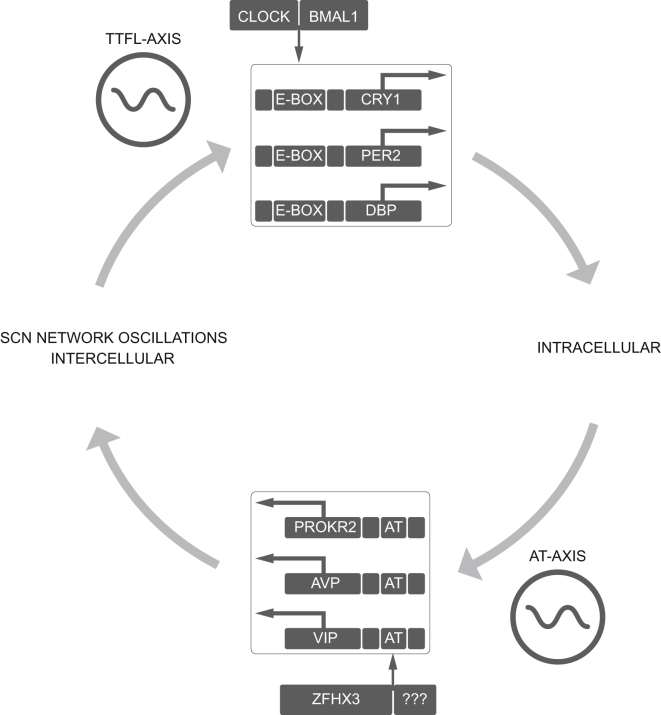
Model Depicting the Role of the ZFHX3/AT Axis in Maintaining SCN Oscillations A diagram outlining the proposed mechanism for maintaining robust circadian oscillations in the SCN. The ZFHX3/AT axis is sensitive to the TTFL and, in turn, feeds back temporal information to the TTFL through its neuropeptidergic-dependent effects on intercellular, circuit-level signaling.

It is clear from our results that the period of AT-driven transcription is sensitive to the period of the TTFL, as evidenced by the effect of CK1 inhibition. This argues that the ZFHX3/AT axis is downstream of the TTFL. However, it is also affected by the *Zfhx3*^*Sci*^ mutation, as is the period of the TTFL, as reported by PER expression. The TTFL and AT axes are, therefore, reciprocally dependent, each able to influence the other. We propose that this arises from the circuit-level effects of AT-driven transcription, specifically of neuropeptide-encoding genes, leading to a logical module in which the TTFL affects AT function. In turn, AT motif activation affects neuropeptidergic expression, leading to cell-nonautonomous effects on circuit-level signaling in the SCN, and ultimately influences TTFL activity (Figure 7).

The S*ci* mutation appears to predominantly influence circadian rhythmicity in a cell-nonautonomous manner by directly regulating the expression of specific neuropeptides that, in turn, synchronize local networks within the SCN. Nevertheless, we cannot rule out the possibility that this obscures a smaller cell-autonomous effect of *Sci*. Unlike many core clock genes, *Zfhx3* is not ubiquitously expressed, so any cell-autonomous influence would be limited to regions where it is expressed, such as the SCN. In keeping with this, we found a significant, albeit small, effect on the period of *Per2*-promoter-driven oscillation in both ex vivo SCN slices and in vitro overexpression assays. Although a previous large-scale siRNA screen failed to find significant effects of two Zfhx3 siRNAs on period length in U20S cells, both siRNAs did lengthen the period (test siRNAs: 25.99 ± 0.43 hr and 25.83 ± 0.21 hr; control: 25.20 ± 0.55 hr, mean ± SD) (Zhang et al., 2009), a trend consistent with the significant effects reported here. Further studies investigating the effects of *Sci* on circadian reporter oscillations in individual ZFHX3-positive cells would further elucidate the nature of any small, cell-autonomous role for *Sci*.

Our results indicating that mutant ZFHX3 directly influences the expression of neuropeptides in adult SCN are consistent with this model. As the intercellular coupling driven by neuropeptides synchronizes intracellular molecular clockworks of individual neurons (Maywood et al., 2006, 2011; Liu et al., 2007), misregulation of these neuropeptides will provoke disruptions in circadian parameters. This disruption in *Zfhx3*^*Sci/+*^ mice may underlie the decreased free-running period and amplitude seen for locomotor activity in vivo and PER2::LUC oscillations in ex vivo SCN. The larger distributions of cellular period length and phase seen in individual neurons in Zfhx3^Sci*/+*^ SCN cultures support this disruption of critical neuropeptides. Similarly, features of the Zfhx3^Sci*/+*^ phenotype have been reported in neuropeptidergic mutants. Mice lacking VIP either are arrhythmic or have a shorter period and decreased amplitude compared to controls (Aton et al., 2005; Brown et al., 2007). There is also a decrease in the amplitude of locomotor activity in both *Prok2*- and *Prokr2-*deficient mice (Prosser et al., 2007; Li et al., 2006). Ultimately, these findings suggest that decreased transcriptional activation of the neuropeptide complex seen in *Zfhx3*^*Sci/+*^ mice may account for its circadian phenotype. The circadian activation seen for the AT motif in SCN slices was mirrored by the rhythmic expression of *Grp*, but not *Vip*, mRNA in SCN. As *Vip* expression is likely regulated by light stimulation (Shinohara et al., 1993), this discrepancy may be due to differences in light input between intact SCN in vivo and SCN slices. Recent studies have additionally suggested that both AVP and VIP signaling play a role in entrainment (An et al., 2013: Yamaguchi et al., 2013). The *Zfhx3*^*Sci/+*^ mutant also shows deficits in entrainment under various conditions (Bacon et al., 2004). As pre-treatment with VIP facilitates re-entrainment (An et al., 2013), the decreases in VIP expression in *Zfhx3*^*Sci/+*^ SCN may partially underlie these deficits in entrainment in *Zfhx3*^*Sci/+*^ mice.

We found that knockdown of *Zfhx3* in the SCN of wild-type mice results in an altered period, which suggests that *Zfhx3* has a function in adult brain, aside from its developmental role (Ishii et al., 2003). Nevertheless, we considered whether the *Zfhx3*^*Sci/+*^ circadian phenotype is due partially to differences in terminal differentiation of SCN peptidergic cells, as reported recently for *Lhx1* mutants (Bedont et al., 2014; Hatori et al., 2014). We found no difference in the number of neuropeptide immunopositive cells in adult SCN; thus, there is no direct evidence that a deficit in terminal differentiation explains the circadian deficit in *Zfhx3*^*Sci/+*^. These observations imply that ZFHX3 regulates neuropeptide levels in adult SCN independently of its roles in developing tissue. The sustained expression of ZFHX3 in adult SCN (Lein et al., 2007; VanDunk et al., 2011) is further compatible with its playing such a role. *Zfhx3* is not the first circadian gene to be shown to play this dual role. The *Rora* mutant *staggerer*, for example, has independent roles in both cerebellum development and circadian regulation in adults (Gold et al., 2007). Given that the SCN in adult *Zfhx3*^*Sci/+*^ mice is slightly smaller, we cannot rule out the possibility that developmental changes may contribute to the adult circadian phenotype. Defining the adult versus embryonic roles for *Zfhx3* will be resolved by investigating various regional and stage-specific conditional *Zfhx3* knockout mice.

Our initial finding that *Zfhx3*^*Sci*^ fails to drive expression via the AT motif in vitro suggested to us that knockdown of *Zfhx3* would lead to a similar shortened period in vivo. The apparent lack of congruency between *Zfhx3*^*Sci*^ and *Zfhx3* knockdown effects in vivo could be related to a number of factors. In the first instance, the results of RNA sequencing confirm that many genes in SCN are either positively or negatively regulated by ZFHX3. The integrated effects of mutant and wild-type ZFHX3 in vivo need not necessarily solely reflect the outcome of in vitro studies at *Avp* and *Vip* promoters. Furthermore, previous literature confirms both positive and negative regulatory roles for ZFHX3 and that activation state is dependent on the expression of additional cofactors and the interactions of ZFHX3 with these cofactors (Qi et al., 2008; Mori et al., 2007; Yasuda et al., 1994). Although we have yet to explore this, mutant ZFHX3 may be interfering with these interactions. These observations would support our interpretation that *Sci* is a dominant-negative mutation. Allelic mutations with opposing effects on period length are not unprecedented in circadian biology as evidenced, for example, by *per* locus mutations in *Drosophila* (Konopka and Benzer, 1971).

### Conclusions

We found that the circadian phenotype in *Sci* is caused by a mutation in *Zfhx3*, a transcription factor highly expressed in adult SCN. The mutant protein has a diminished ability to activate a consensus AT motif, leading to decreased expression of a class of neuropeptides critical for intercellular synchrony and rhythm amplitude in the SCN. Importantly, the absence of gross differences in peptidergic cell numbers in adult *Zfhx3*^*Sci/+*^ SCN and the significant effects of gene knockdown postnatally suggest that this regulation occurs independently of any putative developmental effects of *Zfhx3*. Furthermore, we show that reporters driven by the consensus AT motif are activated in a circadian fashion in ex vivo SCN slices and that the rhythm of AT-driven transcription is sensitive to the *Zfhx3*^*Sci/+*^ mutation and the TTFL of the core clockwork. Thus, the activation of regulatory AT motifs by direct interaction with ZFHX3 plays a significant role in maintaining the circadian transcriptional landscape of the SCN. Moreover, the ZFHX3/ AT axis sits within an unprecedented logical module within the SCN. Its function is sensitive to the TTFL and, in turn, because of its neuropeptidergic effects on circuit level signaling, it feeds back temporal information to the TTFL. This module, therefore, encompasses cell-autonomous and circuit-level circadian pacemaking, incorporating them into a coherent oscillation to drive circadian behavior.

## Experimental Procedures

### Mice

All animal studies were performed under the guidance issued by the Medical Research Council in Responsibility in the Use of Animals for Medical Research (July 1993) and Home Office Project Licenses 30/2686 and 80/2310, with local ethical approval.

### Determination of Zfhx3-Specific AT Motif

**Figure S6:**
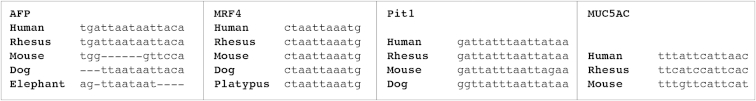
Conservation of ZFHX3 Binding Sites across Species, Related to Experimental Procedures A multiple sequence alignment for ZFHX3 binding sites for *Afp*, *Mrf4*, *Pit1*, and *Muc5ac* reveals conservation of the motif across both primates and placental mammals.

To construct a motif-binding model for ZFHX3, we searched the literature for previously identified binding sites (Figure S6). We used sequences from genes that are directly regulated through a characterized binding motif and generated the consensus sequence using a mixture model by multiple EM (expectation maximization) for motif elicitation (MEME) (Bailey and Elkan, 1994).

### Ex Vivo SCN Slice Experiments

Brains were removed and sectioned as reported previously (Maywood et al., 2006). Bioluminescent emissions from PER2::LUC SCN and lung slices were recorded using photomultiplier tubes (PMT; Hamamatsu) and CCD cameras (Hamamatsu) as described previously (Maywood et al., 2013). Lentiviral transduction of SCN slices was performed as described previously (see Supplemental Experimental Procedures) (Brancaccio et al., 2013).

### RNA Sequencing

RNA sequencing was conducted at the Oxford Genomics Centre (Wellcome Trust Centre for Human Genetics, University of Oxford). See Supplemental Experimental Procedures for more details, including SCN collection, RNA extraction, and qPCR validation.

### RNA-Sequencing Analysis and Network Analysis

Whole transcriptome analysis was carried out using a custom-developed pipeline (see Supplemental Experimental Procedures).

### Motif Analysis

The position frequency matrix of the ZFHX3 binding site, termed AT motif, was determined by MEME. Proximal enhancer regions (450 bp upstream and 50 bp downstream of the TSS) were searched for motif occurrence in the differentially expressed genes (q < 0.05) from the combined analysis (see RNA Sequencing section earlier). We identified the ZFHX3 binding site with the Pscan software (Zambelli et al., 2009), with a threshold of >0.88 defining positive hits.

### Luciferase Reporter Gene Assays

We used the Dual-Luciferase Reporter Assay (Promega) to quantify the luciferase activity (see Supplemental Experimental Procedures for more details).

### Immunofluorescence

**Figure S7:**
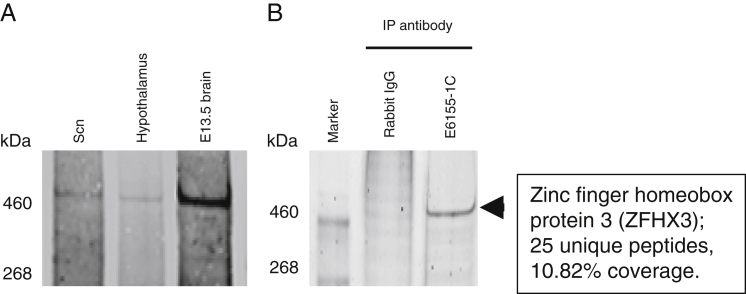
Validation of the ZFHX3 Antibody, Related to Experimental Procedures Expression and characterization of ZFHX3 in mouse tissues. (A) Western blot analysis showing ZFHX3 immunoreactive product in mouse SCN, hypothalamus, and embryonic brain lysates. (B) Immunoprecipitation of ZFHX3 using anti-ZFHX3 antibody and validation by mass spectrometry.

Confocal microscopy was used to image immunofluorescence in free-floating brain sections (40 μm) using a range of antibodies and quantified using ImageJ software (Figure S7; Supplemental Experimental Procedures).

## Author Contributions

M.J.P. conducted the positional cloning, RNA extractions, the qPCR, and the in vitro reporter gene assays. R.S., S. Sethi, M.M.S., and A.-M.M. conducted the bioinformatic analysis for the RNA sequencing data. S. Saito provided experimental materials. E.S.M. and M.H.H. conducted the ex vivo SCN slice work. M.B. performed lentiviral transductions of SCN slices and characterized the circadian oscillations of the AT motifs. E.S.M. and N.J.S. conducted the immunofluorescence. P.L.O. and M.J.F. conducted the ChIP experiments. A.J., Y.C., and R.G.F. planned and conducted the in vivo RNAi experiments. A.R.B., G.J., J.E.C., J.K.E., R.B., and S.W. all aided in the phenotyping, breeding, and management of the mouse colonies. C.E. validated the ZFHX3 antibody. P.M.N. developed the underlying scientific research project. M.H.H., M.J.P., M.B., E.S.M., and P.M.N. all aided in planning the experimental approach. All authors aided in writing the manuscript.

## Accession Numbers

The accession number for the Sci allele reported in this paper is MGI: 2679554.

## Supplemental Information

Document S1. Supplemental Experimental Procedures, Figures S1–S7, and Tables S2–S6

Table S1. List of Differentially Expressed SCN Transcripts from the RNA Sequencing Data, Related to Figure 2 Differential gene list predicted from RNA-sequencing data using three different tools at time intervals ZT3 and ZT15 (p < 0.05, log_2_ fold change >1).

Document S2. Article plus Supplemental Information
