# Supplementary material for: The Regulatory Factor ZFHX3 Modifies Circadian Function in SCN via an AT Motif-Driven Axis
Source: Cell. 2015 Jul 30;162(3):607–21. doi: 10.1016/j.cell.2015.06.060 (PMC4537516; doi:10.1016/j.cell.2015.06.060)
Supplement: Document S1. Supplemental Experimental Procedures, Figures S1–S7, and Tables S2–S6 [file mmc1.pdf]

Cell
Supplemental Information

The Regulatory Factor ZFHX3 Modifies Circadian Function in SCN via an AT Motif-Driven Axis

Michael J. Parsons, Marco Brancaccio, Siddharth Sethi, Elizabeth S. Maywood, Rahul Satija, Jessica K. Edwards, Aarti Jagannath, Yvonne Couch, Mattéa J Finelli, Nicola J. Smyllie, Christopher Esapa, Rachel Butler, Alun R. Barnard, Johanna E. Chesham, Shoko Saito, Greg Joynson, Sara Wells, Russell G. Foster, Peter L Oliver, Michelle M. Simon, Ann-Marie Mallon, Michael H. Hastings, and Patrick M. Nolan

Supplemental Experimental Procedures

Mice

When not being tested, mice were housed in individually ventilated cages under 12/12 h light/dark (LD) conditions with food and water available *ad libitum*. Inbred strains and mutant colonies were bred and maintained at MRC Harwell. The $Zfhx3^{Flox/+}$ mice were imported from Dr. Dong's laboratory (Emory University)and are described in detail elsewhere (Sun et al., 2012).

ENU Screen and Genetic Localization of the *Sci* Circadian Mutant

Wheel-running activity was used to identify circadian phenodeviants following ENU mutagenesis (Bacon et al., 2004). The original *Sci* founder, on a C3H/HeH x BALB/cAnNCrl background, displayed reduced amplitude, a short circadian period length under constant darkness and a reduced or absent phase shift in response to a light pulse in constant darkness. The mutant phenotype was tested for inheritance, crossing to C3H/HeH and the behavioral phenotype was completely penetrant on this background. Low resolution mapping identified a novel mutant locus to be associated with circadian rhythms, from 101.86 Mb to 110.46 Mb on distal chromosome 8 (see Bacon et al., 2004), followed by positional sequencing of *in silico* prioritized candidate genes to reveal the causal mutation. All genes within this locus underwent complete Sanger sequencing of their coding regions. Besides the Sci mutation, no other mutations were detected. The *Sci* animals were bred onto both C57BL/6J and C3H/HeH backgrounds and backcrossed for 10 generations until congenic, ensuring that other functional ENU induced mutations were removed. Breeding difficulties however were encountered on both backgrounds. To circumvent these problems, the *Sci* animals were maintained on a mixed C3H/HeH x C57BL6/J F1 background. The mutation for *Sci* was genotyped using a real time PCR (RT-PCR) Taqman assay. DNA extracted from ear biopsies was diluted 1:20 in H_2O for the reaction (forward primer: TCCACGCATTGCTTCAGATG; reverse primer: TGTGCCTTCTGCTTGTTCTCA; wildtype probe:

CTTTGAGCTCGTCATT; mutant probe: TTTGAGCTCTTCATTCA). Circadian wheel running analysis was performed as outlined in (Banks and Nolan, 2011). Mice were individually housed in cages within light-tight chambers, kept under environmentally controlled conditions. Cages contained running wheels in which the revolutions were monitored and plotted as activity on double-plotted actograms. Initially, cages were exposed to a week-long entrainment period of a 12:12 light-dark cycle, followed by two weeks of constant darkness to assess the free-running ability. A light intensity of 100 lux using a fluorescent light source was used for all screens. Food and water were provided *ad libitum*. Littermate controls were run alongside heterozygous mutants.

SCN Collection and RNA Extraction

SCN punches were collected as in (Jagannath et al., 2013). We verified the quality of the dissection by checking for enrichment of *Six6,* a SCN specific marker, compared to cortical punches. Total RNA was extracted using the RNAeasy column method (QIAGEN, Hilden, Germany). Quality and quantity of RNA were measured using an Agilent Bioanalyzer and a Nanodrop1000 (Thermo Fisher Scientific, Waltham, MA USA), respectively.

qPCR

cDNA was synthesized from 100 ηg of SCN RNA using the Invitrogen SuperScript III First Strand cDNA Synthesis SuperMix (Life Technologies, Grand Island, NY), and qPCR was conducted with Sybr green I using an SDS7700 thermal cycler (Applied Biosystems, Foster City, CA). Relative quantification of transcript levels was carried out as described previously (Jagannath et al., 2013). The geometric mean of three housekeeping genes was used for normalization (*Gapdh*, *B2m, Psmb2)*. Primer sequences are provided in **Table S5**.

Luciferase Reporter Gene Assays

We used Jetprime (Polyplus Transfection, Illkirch, France) to co-transfect 100 ng of the pGL3 vectors, 400 ng of the Zfhx3 pcDNA™3.1/V5-His vector and 5 ng of the pRL-TK Vector (renilla) into HEK293 cells. We used the Dual-Luciferase Reporter Assay (Promega, Madison, WI) to quantify the luciferase activity, normalizing to renilla activity (Varioskan, Thermo Fisher Scientific Inc., Waltham, MA, USA). All experiments were conducted in triplicate.

RNA Sequencing

We sent 500 ηg of SCN RNA samples to the Oxford Genomics Centre (Wellcome Trust Centre for Human Genetics, University of Oxford). The samples underwent poly-A selection, after which two multiplexed DSN Library Preparations (6 samples/multiplex) were generated. Each multiplex was run on one 50bp PE lane of a HiSeq2000.

RNA Sequencing Analysis

Sequence data were aligned to the genome using TopHat (Trapnell et al., 2009) resulting in counts for genomic regions where reads accumulate. Read counts were calculated using HTSeq (Anders et al., 2014) and statistical analysis of differentially expressed genes was determined using three softwares; EdgeR (Robinson et al, 2010), DESeq (Anders and Huber, 2010) and Cufflinks (Trapnell et al., 2010), default parameters were used in all cases. A filtering strategy was employed on all three datasets to reduce false positives. Genes with $q>0.05$ and log2 fold change < 1 were removed from further analysis. Remaining genes common to at least two datasets were used for validation and further analysis. The RNA sequence data has been deposited in the European Nucleotide Archive (accession ID: PRJEB9284).

Network Analysis for RNA Sequencing Results

Protein-protein interactions for significant differentially expressed genes were obtained from STRING (Franceschini et al., 2013), visualization was performed with Cytoscape (Shannon et al., 2003). Clusters of densely connected nodes were found using the default parameters in the Molecular Complex Detection (MCODE) (Bader and Hogue, 2003) plugin for Cytoscape. Functional enrichment of Gene Ontology terms was performed using g:Profiler. Significant terms were determined using the default settings and corrected for multiple testing using g:Profiler's native method g:SCS (Reimand et al., 2007). The threshold for significance was $p<0.05$.

Determination of Zfhx3 Specific AT Motif

To construct a motif-binding model for ATBF1, we searched the literature for previously identified binding sites. We found four cases (*Afp*, *Mrf4*, *Muc5ac*, *Pit1*) (Berry et al., 2001; Mori et al., 2007; Qi et al., 2008; Yasuda et al., 1994) where ATBF1 was shown to directly regulate a downstream gene through a characterized binding motif, as primarily demonstrated by luciferase assays. Examining a mammalian multiple sequence alignment on the UCSC genome browser (Blanchette et al., 2004), we observed that ATBF1 binding sites were conserved across both primates and placental mammals (**Figure S6**). We therefore constructed a preliminary binding motif for ATBF1 on the sequences in **Figure 3A** using a mixture model by expectation maximization (MEME) (Bailey and Elkan, 1994).

***Ex vivo* SCN Slice Experiments**

For Zfhx3$^{flox/+}$ ex vivo experiments, SCN slices from Zfhx3$^{+/+}$ and Zfhx3$^{flox/+}$ mice were transduced by LV encoding for AT-luc. One week after LV transduction, PMT recording of AT-luc was started and performed for >7days. Subsequently, Zfhx3$^{flox/+}$ and Zfhx3$^{+/+}$ matched samples were transduced with AAV expressing Syn-mCherry:Cre (Penn Vector Core) to delete the Zfhx3 floxed allele, and immediately

put back for PMT luciferase recording. Period was assessed before AAV treatment and after >7 days to allow for effective Cre recombination. Following recordings, Zfhx3 expression levels in slices were determined by qPCR.

Vector Construction

Mouse *Zfhx3* cDNA was cloned into the pcDNA™3.1/V5-His vector (amino acid positions 1787 to 3723 from transcript ENSMUSG00000038872). The Zfhx3^{Sci} mutation was introduced into this construct using site-directed mutagenesis using the QuikChange® II XL Site-Directed Mutagenesis Kit (Agilent Technologies, Santa Clara, CA, USA). The CLOCK and BMAL expression vectors were generously provide by Dr. Filippo Tamanini. The DBP expression vector was generously provide by Dr. Jürgen Ripperger. To generate the AT motif driven luciferase reporter vectors, we generated forward and reverse oligonucleotides containing x7 consensus motif sequence ATTTAATTAT with an AACT linker sequence and *XhoI* sites on either side, hybridized these oligos and cloned them into both the pGL3-Enhancer and lentivirus Luciferase reporter vectors. We cloned approximately 1 kb of the putative promoters for *Avp*, *Drd1a*, *Grp*, *Prokr2*, *Vip and Vipr2* upstream of the pGL3-Enhancer Luciferase Reporter vector (see **Table S6**). The pGL3-Enhancer Luciferase Reporter vector containing the Cry1 and Cry2 promoters were generous provided by Dr. Saito. The pGL3-Enhancer Luciferase Reporter vector containing the Per1 and Per2 promoters were provided by Dr. Hastings. To test the specificity of the AT motif, we mutated its 3 most conserved residues (residues 6, 8 and 10) in the pGL3-Enhancer Luciferase Reporter vector containing the *Avp* and *Vip* promoters.

Antisera

Two peptides corresponding to amino acid residues 21-49 (1A) and 2114-2154 (1C) of mouse ZFHX3 were synthesized and used to immunize rabbits (Epitomics). Antisera were affinity purified on Sulfolink coupling gel (Pierce) before antibody characterization. Western blot analysis using the affinity purified antibodies identified immunoreactive products of ~460kDa in mouse SCN, hypothalamus and E13.5 embryonic brain lysates (**Figure S7A**). In order to verify the identity of this immunoreactive product, 4.5mg of E13.5 embryonic brain lysate was immunoprecipitated using anti-ZFHX3 antibody overnight at 4°C, followed by incubation with protein G sepharose beads for 2h at 4ºC. Bound proteins were eluted from the beads by adding 40µl LDS sample buffer (Invitrogen) and heating at 95ºC for 5min. Eluted proteins were separated on 3-8% Tris-Acetate gel and stained with AquaStain (**Figure S7B**). Analysis by mass spectrometry identified ZFHX3 as the major protein in the ~460 kDa gel band thereby confirming the specificity of the anti-ZFHX3 antibody.

Immunofluorescence

Confocal microscopy was used to image immunoflourescent staining from free-floating brain sections (40um) (using rabbit anti-ZFHX3 (1:2000 see supplemental methods), rabbit anti-AVP (1:1000 Bachem, USA), guinea-pig anti-VIP (1:1000 Bachem, USA) and rabbit anti-GRP (1:1000 Immunostar, USA) primary antibodies. Alexa-Fluor secondary antibodies (Invitrogen, USA) were used at a dilution of 1:1000 (594 goat anti-rabbit or 488 goat anti- guinea-pig). Sections were slide-mounted using Vectashield mounting medium plus DAPI (Vector Laboratories, USA). To quantify the immunostaining, confocal images were analyzed using ImageJ software by outlining the SCN from the DAPI stained image and using this template to measure the relative intensity of the immunostaining for each neuropeptide. Where more than one section was analyzed from an animal, the mean of the consecutive measures were used for that individual.

In Vivo RNAi

In vivo RNAi experiments were conducted as previously reported (Jagannath et al., 2013). We used the *in vivo* formulation of siRNAs for *Zfhx3* (assay ID: ss62675; Life Technologies, United Kingdom). Briefly, the siRNA was complexed with Invivofectamine 2.0 (Invitrogen) prepared according to manufacturer's instructions and concentrated using Amicon Ultracel-100 to 5ug/ul. 1ul of this mix was delivered using microinjection into the third ventricle of anaesthetised mice, with sterotaxic equipment with coordinates as described previously (Butcher et al., 2002; Cheng et al., 2007). Mice were allowed to recover and returned to the light-tight chambers for further experiments. To measure silencing of *Zfhx3*, mice were injected with either siNT or siZfhx3 and 96 hours later were sacrificed. SCN was collected, RNA extracted and qPCR conducted on the relevant mRNAs. For studies on circadian period length, C57Bl/6 mice (8 weeks of age) were maintained on running wheels in light tight chambers on a 12:12 LD cycle (400 lux from white LED lamps) and injected with siRNA as above. 1 day after the injection, the mice were placed in DD and running wheel activity data were collected and analysed on Clocklab (Actimetrics, Wilmette, IL). Data on onset of activity for 10 days following treatment were used to calculate period length.

Chromatin Immunoprecipitation (ChIP)

Assays were performed using the ChIP-IT enzymatic kit according to the manufacturer's instructions (Active Motif). Briefly, SCN hypothalamic punches were pooled from four sex-matched mice at ZT3 and immediately fixed for 10 min at room temperature with gentle shaking in 1% formaldehyde. The cross-linking reaction was stopped by addition of glycine to a final concentration of 0.25 M. Cross-linked chromatin was sheared by enzymatic digestion following the manufacturer's recommendations. Samples were incubated with 3µg of antiserum against ZFHX3, together with magnetic beads, on a rotator at 4°C overnight. This was followed by crosslinking reversal and proteinase-K digestion as per the

manufacturer's protocol. The recovered DNA was purified using the QIAquick PCR purification kit (Qiagen) and subjected to quantitative PCR using Fast SYBR Green (Life Technologies). Primer pairs were designed upstream of the transcription start site and within the coding region of Avp and Vip with at least one pair covering a predicted AT motif, in addition to the promoter of the negative control gene, Gapdh. The binding of ZFHX3 to adjacent promoter regions (primers spanning approximately 160 bp) was calculated using the ΔΔCt method; non-immunoprecipitated input (diluted 1:10) and primers in the coding region of each gene were used to normalise each reaction. Three independent cohorts of four animals were used as biological replicates and each individual PCR reaction was carried out in triplicate. Primer sequences are available upon request.

Supplemental References

Anders, S., Huber., W. (2010). Differential expression analysis for sequence count data. Genome Biol. *11*,R106.

Anders, S., Pyl, P.T., Huber, W. (2014). A Python framework to work with high-throughput sequencing data. Bioinformatics. 638.

Bacon, Y., Ooi, A., Kerr, S., Shaw-Andrews, L., Winchester, L., Breeds, S., Tymoska-Lalanne, Z., Clay, J., Greenfield, A. G., Nolan, P. M. (2004). Screening for novel ENU-induced rhythm, entrainment and activity mutants. Genes, Brain and Behavior, *3*,196–205.

Bader, G.D., Hogue, C.W. (2003). An automated method for finding molecular complexes in large protein interaction networks. BMC Bioinformatics. *13*,4:2.

Bailey, T.L., Elkan, C. (1994). Fitting a mixture model by expectation maximization to discover motifs in biopolymers. Proceedings of the Second International Conference on Intelligent Systems for Molecular Biology, pp. 28-36, AAAI Press, Menlo Park, California.

Banks, G.T., Nolan, P.M. (2011). Assessment of circadian and light-entrainable parameters in mice using wheel-running activity. Curr. Prot. Mouse Biol. *1*,369-381.

Berry, F.B., Miura, Y., Mihara, K., Kaspar, P., Sakata, N., Hashimoto-Tamaoki, T., Tamaoki, T. (2001). Positive and negative regulation of myogenic differentiation of C2C12 cells by isoforms of the multiple homeodomain zinc finger transcription factor ATBF1. J Biol Chem. *276*,25057-65.

Blanchette, M., Kent, W.J., Riemer, C., Elnitski, L., Smit, A.F., Roskin, K.M., Baertsch, R., Rosenbloom, K., Clawson, H., Green, E.D., et al. (2004). Aligning multiple genomic sequences with the threaded blockset aligner. Genome Res. *14* ,708-15.

Franceschini, A., Szklarczyk, D., Frankild, S., Kuhn, M., Simonovic, M., Roth, A., Lin, J., Minguez, P., Bork, P., von Mering, C., Jensen, L.J. (2012). STRING v9.1: protein-protein interaction networks, with increased coverage and integration. Nucleic Acids Res. *41*,D808-15.

Jagannath, A., Butler, R., Godinho, S.I., Couch, Y., Brown, L.A., Vasudevan, S.R., Flanagan, K.C., Anthony, D., Churchill, G.C., Wood, M.J., et al. (2013). The CRTC1-SIK1 pathway regulates entrainment of the circadian clock. Cell. *154*,1100-11.

Mori, Y., Kataoka, H., Miura, Y., Kawaguchi, M., Kubota, E., Ogasawara, N., Oshima, T., Tanida, S., Sasaki, M., Ohara, H., et al. (2007). Subcellular localization of ATBF1 regulates MUC5AC transcription in gastric cancer. Int J Cancer. *121*,241-7.

Qi, Y., Ranish, J.A., Zhu, X., Krones, A., Zhang, J., Aebersold, R., Rose, D.W., Rosenfeld, M.G., Carrière, C. (2008). Atbf1 is required for the Pit1 gene early activation. Proc Natl Acad Sci U S A. *105*,2481-6.

Reimand J., Kull M., Peterson. H., Hansen, J., Vilo, J. (2007). G:Profiler - a web-based toolset for functional profiling of gene lists from large-scale experiments. Nucleic Acids Res. 35,W193-W200.

Robinson, M.D., McCarthy, D.J. Smyth, G.K. (2010). EdgeR: a Bioconductor package for differential expression analysis of digital gene expression data. Bioinformatics. *26*,139-40.

Shannon, P., Markiel, A., Ozier, O., Baliga, N.S., Wang, J.T., Ramage, D., Amin, N., Schwikowski, B., Ideker, T. (2003). Cytoscape: a software environment for integrated models of biomolecular interaction networks. Genome Res. *13*,2498-504.

Sun X, Fu X, Li J, Xing C, Martin DW, Zhang HH, Chen Z, Dong JT. (2012) Heterozygous deletion of Atbf1 by the Cre-loxP system in mice causes preweaning mortality. Genesis. *50*, 819-27.

Trapnell, C., Pachter, L., Salzberg, S.L. (2009). TopHat: discovering splice junctions with RNA-Seq. Bioinformatics *25*,1105-11.

Trapnell, C., Williams, B.A., Pertea, G., Mortazavi, A., Kwan, G., van Baren, M.J., Salzberg, S.L., Wold, B.J., Pachter, L. (2010). Transcript assembly and quantification by RNA-Seq reveals unannotated transcripts and isoform switching during cell differentiation. Nat Biotechnol *28*,511-5.

Yasuda, H., Mizuno, A., Tamaoki, T., Morinaga, T. (1994). ATBF1, a multiple-homeodomain zinc finger protein, selectively down-regulates AT-rich elements of the human alpha-fetoprotein gene. Mol Cell Biol. *14*,1395-401.

Table S2. Gene Ontology Enrichment of Differentially Expressed Genes, Related to Figure 2

Profiler was used to find the enriched molecular function and biological process terms for the differential gene list predicted from RNA-sequencing data at time interval ZT3 and ZT15 ($q<0.05$). BP is biological process.

GO ID	p-value	X	GO Category	Term Name
**GO:0044057**	5.28E-03	12	BP	regulation of system process
**GO:0006139**	6.79E-03	54	BP	nucleobase-containing compound metabolic process
**GO:0031326**	6.87E-03	41	BP	regulation of cellular biosynthetic process
**GO:0021953**	2.32E-02	8	BP	central nervous system neuron differentiation
**GO:0051239**	5.78E-03	31	BP	regulation of multicellular organismal process
**GO:1901360**	8.04E-04	59	BP	organic cyclic compound metabolic process

**GO:1901576**	2.59E-03	53	BP	organic substance biosynthetic process
**GO:0009889**	1.18E-02	41	BP	regulation of biosynthetic process
**GO:0051171**	3.83E-02	41	BP	regulation of nitrogen compound metabolic process
**GO:0034641**	4.92E-04	59	BP	cellular nitrogen compound metabolic process
**GO:0046483**	2.79E-03	56	BP	heterocycle metabolic process
**GO:0006725**	3.52E-03	56	BP	cellular aromatic compound metabolic process
**GO:0044249**	1.21E-03	53	BP	cellular biosynthetic process
**GO:0042127**	1.07E-02	22	BP	regulation of cell proliferation
**GO:0031323**	5.38E-04	54	BP	regulation of cellular metabolic process
**GO:0044708**	8.36E-03	12	BP	single-organism behavior
**GO:0030804**	9.24E-03	6	BP	positive regulation of cyclic nucleotide biosynthetic process
**GO:1900373**	1.17E-02	6	BP	positive regulation of purine nucleotide biosynthetic process
**GO:0030182**	8.43E-03	20	BP	neuron differentiation

Table S3. Gene Ontology Enrichment of Differentially Expressed Genes in Module 1, Related to Figure 3

Profiler was used to find the enriched molecular function - and biological process terms - for the genes in module 1. BP is biological process, MF is molecular function and CC is cellular component.

GO ID	p-value	X	GO Category	Term Name
**GO:0007186**	6.49E-12	17	BP	G-protein coupled receptor signaling pathway
**GO:0007218**	4.17E-09	7	BP	neuropeptide signaling pathway
**GO:0005179**	1.32E-08	7	MF	hormone activity
**GO:0007154**	1.82E-08	20	BP	cell communication
**GO:0001664**	2.24E-08	8	MF	G-protein coupled receptor binding
**GO:0007166**	8.09E-08	17	BP	cell surface receptor signaling pathway
**GO:0007165**	1.09E-07	19	BP	signal transduction
**GO:0005184**	2.76E-06	4	MF	neuropeptide hormone activity
**GO:0007204**	2.29E-05	6	BP	positive regulation of cytosolic calcium ion concentration
**GO:0051241**	2.45E-05	7	BP	negative regulation of multicellular organismal process
**GO:0008015**	2.50E-05	7	BP	blood circulation
**GO:0051480**	4.21E-05	6	BP	cytosolic calcium ion homeostasis
**GO:0044708**	4.36E-05	7	BP	single-organism behavior

**GO:0050880**	1.85E-04	5	BP	regulation of blood vessel size
**GO:0035150**	1.92E-04	5	BP	regulation of tube size
**GO:0006874**	2.25E-04	6	BP	cellular calcium ion homeostasis
**GO:0007200**	2.56E-04	4	BP	phospholipase C-activating G-protein coupled receptor signaling pathway
**GO:0055074**	2.86E-04	6	BP	calcium ion homeostasis
**GO:0072503**	3.09E-04	6	BP	cellular divalent inorganic cation homeostasis
**GO:0003018**	3.21E-04	5	BP	vascular process in circulatory system
**GO:0030819**	3.87E-04	4	BP	positive regulation of cAMP biosynthetic process
**GO:0072507**	4.18E-04	6	BP	divalent inorganic cation homeostasis
**GO:0042311**	4.69E-04	4	BP	vasodilation
**GO:0051239**	6.27E-04	11	BP	regulation of multicellular organismal process
**GO:0030804**	7.10E-04	4	BP	positive regulation of cyclic nucleotide biosynthetic process
**GO:0006875**	7.94E-04	6	BP	cellular metal ion homeostasis
**GO:0007626**	1.09E-03	5	BP	locomotory behavior
**GO:0051050**	1.15E-03	7	BP	positive regulation of transport
**GO:0030003**	1.27E-03	6	BP	cellular cation homeostasis
**GO:0030817**	1.39E-03	4	BP	regulation of cAMP biosynthetic process
**GO:0006873**	1.52E-03	6	BP	cellular ion homeostasis
**GO:0006171**	1.74E-03	4	BP	cAMP biosynthetic process
**GO:0023051**	1.90E-03	11	BP	regulation of signaling
**GO:0010646**	1.92E-03	11	BP	regulation of cell communication
**GO:0055065**	1.99E-03	6	BP	metal ion homeostasis
**GO:0071855**	2.14E-03	3	MF	neuropeptide receptor binding
**GO:0045762**	2.72E-03	3	BP	positive regulation of adenylate cyclase activity
**GO:0050878**	3.37E-03	5	BP	regulation of body fluid levels
**GO:0055080**	3.65E-03	6	BP	cation homeostasis
**GO:0007622**	3.77E-03	3	BP	rhythmic behavior
**GO:0007267**	7.61E-03	7	BP	cell-cell signaling
**GO:0045761**	9.83E-03	3	BP	regulation of adenylate cyclase activity
**GO:0008217**	1.48E-02	4	BP	regulation of blood pressure
**GO:0043270**	1.77E-02	4	BP	positive regulation of ion transport
**GO:0090066**	1.88E-02	5	BP	regulation of anatomical structure size
**GO:0007611**	4.31E-02	4	BP	learning or memory

Table S4. Gene Ontology Enrichment of Differentially Expressed Genes in Module 2, Related to Figure 3

Profiler was used to find the enriched molecular function - and biological process terms - for the genes in module 2. BP is biological process, MF is molecular function and CC is cellular component.

term ID	p-value	X	GO Category	Term Name
**GO:0006412**	4.06E-04	5	BP	translation
**GO:0032991**	7.98E-03	8	CC	macromolecular complex
**GO:0030529**	2.11E-03	5	CC	ribonucleoprotein complex
**GO:0005829**	3.27E-02	5	CC	cytosol
**GO:0044445**	3.58E-06	5	CC	cytosolic part
**GO:0005840**	7.51E-06	5	CC	ribosome
**GO:0044391**	1.23E-06	5	CC	ribosomal subunit
**GO:0015934**	4.12E-03	3	CC	large ribosomal subunit
**GO:0022626**	1.87E-07	5	CC	cytosolic ribosome
**GO:0022625**	1.29E-03	3	CC	cytosolic large ribosomal subunit
**GO:0005198**	2.59E-04	5	MF	structural molecule activity
**GO:0003735**	1.23E-04	4	MF	structural constituent of ribosome

Table S5. Quantitative PCR Primer Sequences, Related to Experimental Procedures

Gene	Forward Primer	Reverse Primer
Avp	GCTGCCAGGAGGAGAACTAC	AAAAACCGTCGTGGCACTC
B2m	GCTATCCAGAAAACCCCTCAA	CATGTCTCGATCCCAGTAGACGGT
Bmal1	CCGTGCTAAGGATGGCTGTT	TTGGCTTGTAGTTTGCTTCTG
Chat	GTGAGACCCTGCAGGAAAAG	GCCAGGCGGTTGTTTAGATA
Clock	TGTCTCAAGCTGCAAATTTACCA	TTTAGATGCTGCATGGCTCCTA
Cry1	GCTATGCTCCTGGAGAGAACGT	TGTCCCCGTGAGCATAGTGTAA
Cry2	TGACCTAGACAGAATCATCGAACTG	GGCTGATGAGGGCCTGAA
Dbp	GAGCCTTCTGCAGGGAAACA	GCCTTGCGCTCCTTTTCC
Drd1a	AAAGATCCAACCCGTTACCC	ACAGCAAGCCCTAGGGAACT
Gal	GTGACCCTGTCAGCCACTCT	GGTCTCCTTTCCTCCACCTC
Gapdh	ACGGGAAGCTCACTGGCATGGCCTT	CATGAGGTCCACCACCCTGTTGCTG
Grp	GCCTCTCAGTCTCCAGCCTA	GCAGTTCCTCCCTTTTCCTT
Nms	GCCAGCAGAAGGATGAAAAG	GGCCTGAAAAGGAAAAATGG
Per1	CCCCTGCCTCCCAGTGA	CTGAAAGTGCATCCTGATTGGA
Per2	AGCTACACCACCCCTTACAAGCT	GACACGGCAGAAAAAAGATTTCTC
Prok2	TGCGACAAGGACTCTCAGTG	TTCTTCTTTCCTGCCTTCCA
Prokr2	GGTCTCCCTGTACGTCTCCA	CAAAGCGATCAGGAAGGAAG
Psmb2	AAATGCGGAATGGATATGAATTG	GAAGACAGTCAGCCAGGTT
Reverba	CGTTCGCATCAATCGCAACC	GATGTGGAGTAGGTGAGGTC
Six6	GTGGGCAACTGGTTCAAAAA	AGATGTCGCACTCACTGTCG

Vip	CAGTTCCTGGCATTCCTGAT	GGTCACCTGCTCCTTCAAAC
Vipr2	GACGTTGGGGAAACTGTCAC	AGCCACACGCATCTATGAAA
Zfhx3	CCAATAGCCTGGAGAAGCTG	AGTTGCACAGGACACAGTGG

Table S6. Design of the Luciferase Reporter Gene Vectors Containing the Differentially Expressed Gene Promoters, Related to Experimental Procedures

Regions of the promoters for a subset of the genes that were differentially expressed in the SCN of *Zfhx3*$^{Sci/+}$ and *Zfhx3*$^{+/+}$ mice were cloned into pGL3-Enhancer Luciferase Reporter vector. These regions included the predicted AT motif. The region of the promoter included and the AT motif location is relative to the start of exon 1 for the given transcript.

**Promoter cloning**					**AT motif**		
**Gene**	**Transcript**	**Forward Primer**	**Reverse Primer**	**Region included**	**Pscan Score**	**Sequence**	**Location**
Avp	ENSMUST00000046001	CTCGAGAGGCATAGAAGCCAGCATGT	CTCGAGGCTGGGCTGCCTATTTATGT	-1673 to -29	0.89	TATTCAACTAT	-325
Drd1a	ENSMUST00000021932	CTCGAGCTCAGGTCACCTCCAGCTTC	CTCGAGGCTTCTGCGGTCAACTCAC	-1818 to +8	0.80	TATTCTCTAAG	-320
Grp	ENSMUST00000025395	CTCGAGGGAACTCAGACCGGAGATTTT	CTCGAGGGAGGGAAACCCTCAGAGC	-781 to +152	0.84	CATCCATCTAC	-345
Prokr2	ENSMUST00000049997	CAGGCAGAACCACAGAATGA	TGGTTGAGGCTTGCCTACTT	-761 to +60	0.82	TACTGAATTAG	-129
Vip	ENSMUST00000019906	CTCGAGCCTGGAATTAAGCC	CTCGAGCTAGGGAAGGCTCC	-1301 to +80	0.91	AATTTAATAAG	-127

		ACAGGA	ACCAGT				
Vipr2	ENSMUST000 00011315	CTCGAGACAA ATGTGCAGGT GGATCA	CTCGAGGCGG ATTTCCTCAGT CTCG	-1115 to +48	0.77	CAAGTAAGCAG	-323
