# Supplementary material for: The Regulatory Factor ZFHX3 Modifies Circadian Function in SCN via an AT Motif-Driven Axis
Source: Cell. 2015 Jul 30;162(3):607–21. doi: 10.1016/j.cell.2015.06.060 (PMC4537516; doi:10.1016/j.cell.2015.06.060)
Supplement: Document S2. Article plus Supplemental Information [file mmc3.pdf]

Cell

Article

The Regulatory Factor ZFHX3 Modifies Circadian Function in SCN via an AT Motif-Driven Axis

Graphical Abstract

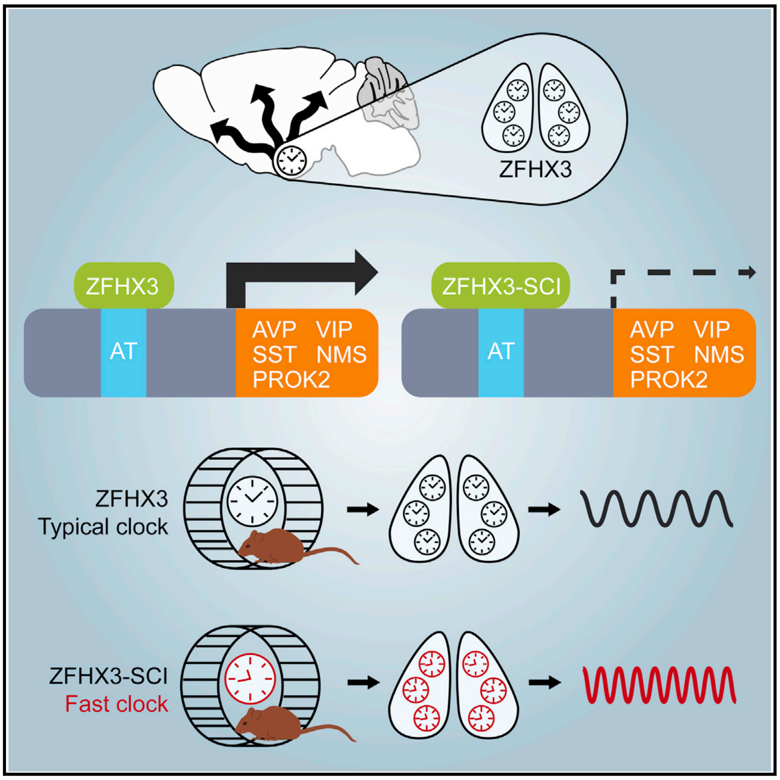

Authors

Michael J. Parsons, Marco Brancaccio, Siddharth Sethi, ..., Ann-Marie Mallon, Michael H. Hastings, Patrick M. Nolan

Correspondence

p.nolan@har.mrc.ac.uk

In Brief

A transcription factor expressed in discrete adult hypothalamic nuclei, including the suprachiasmatic nucleus, regulates circadian locomotor rhythms in vivo through the expression of distinct neuropeptidergic genes to ensure robust synchronous oscillations and circadian rhythms.

Highlights

- *Zfhx3* missense mutation underlies the short circuit (*Zfhx3*^{Sci}) circadian phenotype
- *Zfhx3*^{Sci} reduces the ability of ZFHX3 to activate transcription via AT motifs
- *Zfhx3*^{Sci} phenotype is associated with decreased activation of AT motif in neuropeptide promoters
- Circadian activation in SCN reveals AT motif as a new clock-regulated transcriptional axis

Parsons et al., 2015, Cell *162*, 607–621
July 30, 2015 ©2015 The Authors
http://dx.doi.org/10.1016/j.cell.2015.06.060

Cell

The Regulatory Factor ZFHX3 Modifies Circadian Function in SCN via an AT Motif-Driven Axis

Michael J. Parsons,[1] Marco Brancaccio,[2] Siddharth Sethi,[1] Elizabeth S. Maywood,[2] Rahul Satija,[3,4] Jessica K. Edwards,[1] Aarti Jagannath,[5] Yvonne Couch,[6] Mattéa J. Finelli,[7] Nicola J. Smyllie,[2] Christopher Esapa,[1] Rachel Butler,[5] Alun R. Barnard,[5] Johanna E. Chesham,[2] Shoko Saito,[8,9] Greg Joynson,[1] Sara Wells,[1] Russell G. Foster,[5] Peter L. Oliver,[7] Michelle M. Simon,[1] Ann-Marie Mallon,[1] Michael H. Hastings,[2] and Patrick M. Nolan[1,]*
[1]MRC Harwell, Harwell Science and Innovation Campus, Oxfordshire OX11 0RD, UK
[2]MRC Laboratory of Molecular Biology, Cambridge Biomedical Campus, Cambridge CB2 0QH, UK
[3]New York Genome Center, 101 Avenue of the Americas, New York, NY 10013, USA
[4]Department of Biology, New York University, New York, NY 10012, USA
[5]Nuffield Department of Clinical Neurosciences, University of Oxford, Oxford OX3 9DU, UK
[6]Acute Stroke Program, Radcliffe Department of Clinical Medicine, University of Oxford, Oxford OX3 9DU, UK
[7]MRC Functional Genomics Unit, Department of Physiology, Anatomy and Genetics, University of Oxford, Parks Road, Oxford OX1 3PT, UK
[8]Department of Genetics, Erasmus University Medical Center, 3000 CA Rotterdam, the Netherlands
[9]Faculty of Medicine, University of Tsukuba, 1-1-1 Tennodai, Tsukuba 305-8575, Japan
*Correspondence: p.nolan@har.mrc.ac.uk
http://dx.doi.org/10.1016/j.cell.2015.06.060
This is an open access article under the CC BY license (http://creativecommons.org/licenses/by/4.0/).

SUMMARY

We identified a dominant missense mutation in the SCN transcription factor *Zfhx3*, termed short circuit (*Zfhx3*^{Sci}), which accelerates circadian locomotor rhythms in mice. ZFHX3 regulates transcription via direct interaction with predicted AT motifs in target genes. The mutant protein has a decreased ability to activate consensus AT motifs in vitro. Using RNA sequencing, we found minimal effects on core clock genes in *Zfhx3*$^{Sci/+}$ SCN, whereas the expression of neuropeptides critical for SCN intercellular signaling was significantly disturbed. Moreover, mutant ZFHX3 had a decreased ability to activate AT motifs in the promoters of these neuropeptide genes. Lentiviral transduction of SCN slices showed that the ZFHX3-mediated activation of AT motifs is circadian, with decreased amplitude and robustness of these oscillations in *Zfhx3*$^{Sci/+}$ SCN slices. In conclusion, by cloning *Zfhx3*^{Sci}, we have uncovered a circadian transcriptional axis that determines the period and robustness of behavioral and SCN molecular rhythms.

INTRODUCTION

Circadian rhythms are daily cycles with periods of ~24 hr that affect multiple distinct physiological and behavioral outputs. Temporal regulation of these biological processes is managed, in part, by changes to the 24-hr transcriptional profile within a tissue (Koike et al., 2012). This transcriptional landscape is governed by a number of processes, including modification of DNA structure (Feng et al., 2011), and by the interaction of transcription factors with DNA sequence motifs in gene promoters (Bozek et al., 2009). Some motifs involved in circadian regulation, including E-box, D-box, and Rev-responsive elements (RREs) (Bozek et al., 2009, 2010; Ukai and Ueda, 2010), have been well characterized but have only a limited ability to explain the extensive influence of the core clockwork on circadian patterns of gene expression. Therefore, the discovery and characterization of additional motifs promises to reveal further levels of circadian regulatory complexity and facilitate a system-wide appreciation of the principles that underpin the temporal orchestration of gene expression.

In mammals, the current canonical model of the core molecular clock consists of a well-characterized transcriptional-translational feedback loop (TTFL), where CLOCK and BMAL1 drive the expression of E-box-regulated genes, including *Per* and *Cry*, whose protein products, in turn, negatively regulate these same genes by interacting with the CLOCK:BMAL1 complex (Zhang and Kay, 2010). Most cells contain such molecular clocks (Albrecht, 2012) synchronized to each other and to the external environment by signals derived from the principal circadian pacemaker, the suprachiasmatic nucleus (SCN) (Ralph et al., 1990). Within the SCN, the TTFL drives the molecular clock in individual cells, while at the tissue level, these individual SCN cells are synchronized through intercellular coupling to form a coherent oscillator (Aton and Herzog, 2005, Maywood et al., 2006). This coupling is driven by several neuropeptides, including vasoactive intestinal peptide (VIP) (Aton et al., 2005; Maywood et al., 2011; Brown et al., 2005) and gastrin-releasing peptide (GRP) (Albers et al., 1995; Brown et al., 2005). Together, they synchronize the intracellular molecular clockworks of individual neurons while also conferring robustness against perturbations (Liu et al., 2007). Moreover, these and additional peptides, including neuromedin S (NMS) and prokineticin 2 (PROK2),

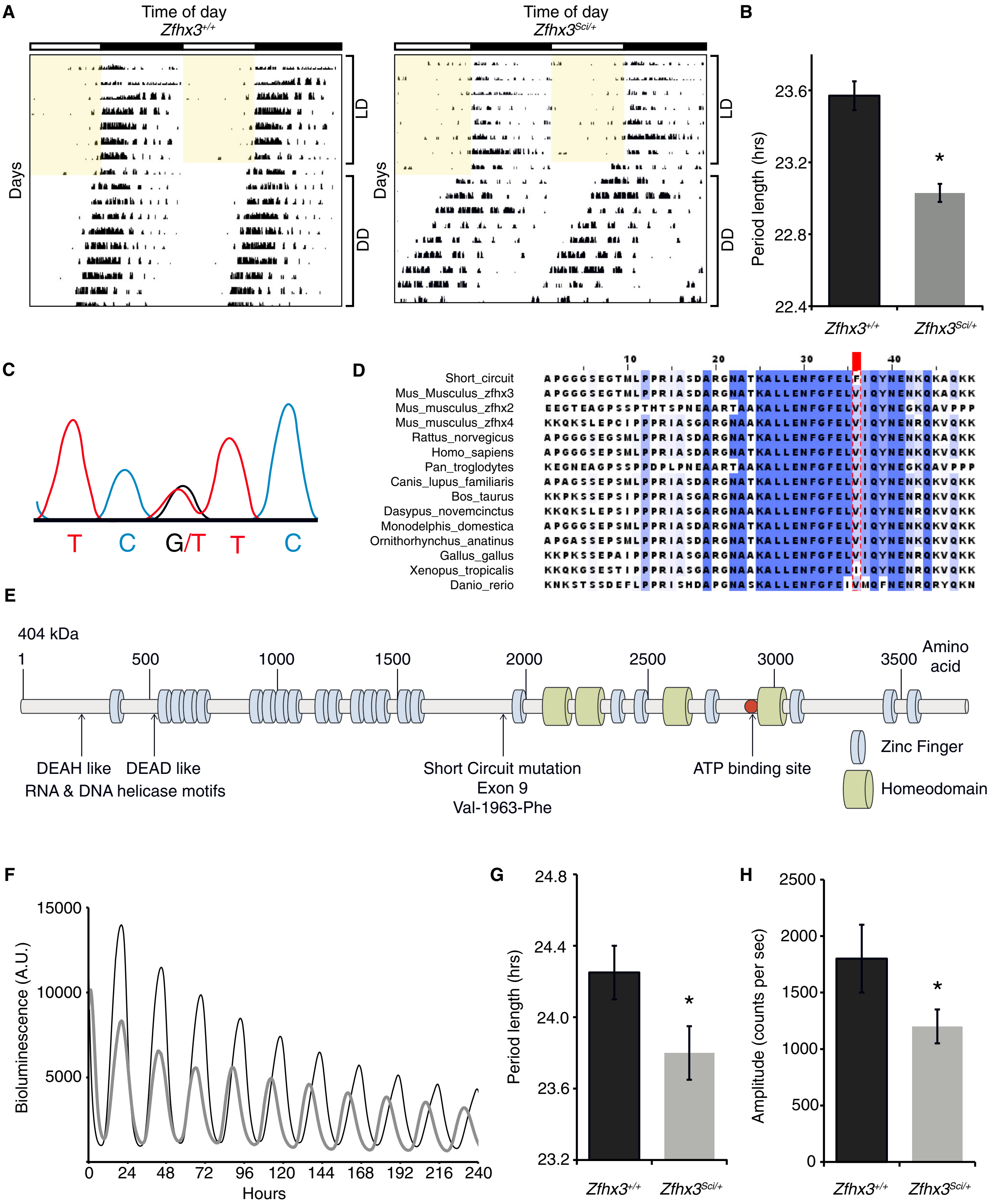

(legend on next page)

are important in conveying circadian signals from the SCN to the rest of the brain and periphery (Li et al., 2006; Prosser et al., 2007; Lee et al., 2015).

Insight into the molecular basis of circadian rhythms in mammals has advanced through gene discovery with the use of numerous approaches, including *N*-ethyl-*N*-nitrosourea (ENU) (Bacon et al., 2004). Discoveries of mammalian circadian genes, including *Clock* (Vitaterna et al., 1994) and *Fbxl3* (Godinho et al., 2007), attest to its utility. Uniquely, ENU-induced mutations can generate not only null alleles but also partial loss-of-function (hypomorphic) and dominant-negative alleles (Acevedo-Arozena et al., 2008). Consequently, ENU mutants have often revealed phenotypes that are novel, different, or more robust than the corresponding knockout mutant, including the dominant-negative $Clock^{\Delta 19}$ mutant (Vitaterna et al., 1994; DeBruyne et al., 2006). Moreover, null mutations in genes with fundamental developmental roles are often embryonic lethal, while corresponding ENU mutants often survive to a later stage than that of the earliest essential function of the gene, thereby enabling the investigation of relevant phenotypes in adults (Acevedo-Arozena et al., 2008).

Here, we investigate the molecular mechanisms underlying the dominant short-circadian-period phenotype in the ENU mutant, short circuit, *Sci* (MGI: 2679554) (Bacon et al., 2004). Through positional candidate analysis, we establish that the *Sci* phenotype is caused by a mutation in the SCN transcription factor *Zfhx3*. This gene acts through transcriptional regulation of genes containing AT motifs in their promoter regions (Yasuda et al., 1994). We found that $ZFHX3^{Sci}$ has a decreased ability to activate a consensus AT motif in vitro. Moreover, using lentiviral transduction of SCN slice cultures, we discovered that activation of AT motifs is under circadian control in SCN and is compromised by the $Zfhx3^{Sci}$ mutation. We further found that, among the transcriptional consequences in adult $Zfhx3^{Sci/+}$ SCN, expression of a number of neuropeptides critical for intercellular signaling was decreased. Using chromatin immunoprecipitation (ChIP), we found that ZFHX3 directly interacts with the AT motif in certain neuropeptide promoters. Importantly, we determined that the effect of the mutation on circadian period was associated with its diminished ability to regulate the transcription of these genes in adult animals. Pharmacological slowing of the TTFL caused a corresponding lengthening of the period of AT activation, suggesting that the ZFHX3/AT axis is sensitive to the core circadian loop. Furthermore, we confirmed that *Sci* is a gain-of-function mutation, as knockdown of *Zfhx3* in vivo and ex vivo can significantly lengthen behavioral and SCN molecular rhythms. In summary, circadian transcription driven by AT motifs is evident in adult SCN and represents a circadian gene-regulatory axis, extending beyond the well-characterized TTFL.

RESULTS

Short Circuit, a Dominant Circadian Mutation in *Zfhx3*

We conducted an ENU screen to uncover genetic factors affecting mammalian circadian behavior (Bacon et al., 2004). Among G1 animals in a dominant screen, we identified a mouse with a circadian period (τ_{DD}; behavioral circadian period of the animals in constant darkness) shorter than the population mean (23.6 ± 0.08 hr; mean ± SEM). This phenotype was inherited in a dominant fashion, with a τ_{DD} (23.0 ± 0.05 hr) ranging from 21.4 to 23 hr (Figures 1A and 1B). We named the mutation short circuit (*Sci*) and mapped the dominant phenotype to mouse chromosome 8 between D8Mit138 (107.67 Mb) and D8Mit213 (110.57 Mb), containing 25 annotated genes. Among the candidates, zinc-finger homeobox 3 (*Zhfx3*) was highly and almost exclusively expressed in adult SCN (Lein et al., 2007). We identified a point mutation in exon 9, resulting in a G → T transversion at position 6620 (*Zfhx3* transcript, ENSMUST00000043896) (Figure 1C). The *Sci* mutation substitutes a phenylalanine for a valine at residue 1963 (V1963F) in a highly conserved region just upstream of the 17th zinc-finger motif (Figures 1D and 1E). Following the identification of the causative mutation, genotype and phenotype correlation demonstrated that the mutation causes homozygous lethality during embryonic development; therefore, only $Zfhx3^{Sci/+}$ adult animals could be assessed phenotypically.

Using ex vivo organotypic slices from $Zfhx3^{Sci/+}$ or $Zfhx3^{+/+}$ animals on a PER2::LUC background, we found that $Zfhx3^{Sci/+}$ SCN had a shorter circadian period and a decreased amplitude of fusion protein expression relative to wild-type ($p < 0.05$, t test) (Figures 1F–1H). Similar decreases were found in individual neurons from $Zfhx3^{Sci/+}$ SCN slices compared to wild-type neurons (Figures S1A and S1B). Furthermore, the period distribution was broader, and RAE increased in individual $Zfhx3^{Sci/+}$ neurons imaged across the SCN circuit (Figures S1C and S1D) ($p < 0.05$, t test). There were no period differences in organotypic lung slices (Figure S1E), suggesting a central oscillator specific effect. These ex vivo SCN findings mirror the differences seen in locomotor behavior and suggest that photic inputs are not necessary for the expression of the short-period phenotype. Conversely, there were no significant differences in mRNA

Figure 1. The Short Circuit (*Sci*) Phenotype Results from a Mutation in the Transcription Factor *Zfhx3*

(A) Representative double-plotted actograms of wheel-running activity in $Zfhx3^{Sci/+}$ and $Zfhx3^{+/+}$ mice (7 days on a 12-hr light:dark (LD) schedule, followed by 2 weeks in constant darkness). Yellow shading represents periods when lights are on. Vertical black bars represent wheel running activity.

(B) $Zfhx3^{Sci/+}$ mice have a shorter free-running period than littermate controls in constant darkness (n = 6). *p = 0.0009.

(C) The $Zfhx3^{Sci}$ mutation mapped to the zinc-finger homeobox 3 (*Zfhx3*) locus and results in a G → T transversion at position 6620.

(D) Multiple protein sequence alignment of ZFHX3 protein and its paralogues. The $Zfhx3^{Sci}$ mutation, a V1963F substitution, is in a highly conserved region.

(E) A schematic of the functional domains of ZFHX3; the $Zfhx3^{Sci}$ mutation lies upstream of a zinc-finger domain.

(F) Representative plots showing circadian activation of PER2::LUC expression in ex vivo SCN organotypic slices from $Zfhx3^{Sci/+}$ (gray line) or $Zfhx3^{+/+}$ (black line) animals.

(G and H) The mean (G) period and (H) amplitude of PER2::LUC expression were decreased in $Zfhx3^{Sci/+}$ (gray bars, n = 29) compared to $Zfhx3^{+/+}$ (black bars, n = 21). *$p < 0.05$, t test.

Error bars indicate SEM.

See also Figures S1 and S2.

expression patterns of core circadian genes in the SCN of *Zfhx3*$^{Sci/+}$ and *Zfhx3*$^{+/+}$ animals sampled at six time points across the light:dark cycle (Figure S2), suggesting that any differences in clock gene expression are masked by the light:dark cycle. Collectively, these data predict that the mutation disrupts a *Zfhx3*-dependent and canonical TTFL-independent effect on SCN circadian period.

Transcriptional Consequences in *Zfhx3*$^{Sci/+}$ SCN

We used RNA sequencing to identify transcriptional targets of ZFHX3. RNA was extracted from SCN tissue punches from *Zfhx3*$^{Sci/+}$ and *Zfhx3*$^{+/+}$ animals at zeitgeber time (ZT)3 and ZT15 (n = 3 for each time by genotype combination). RNA sequencing revealed that 242 genes were differentially expressed at either one or both time points ($\log_2$ fold change > 1, $p < 0.05$) (Table S1), with 28 of those surviving multiple testing correction ($q < 0.05$ in at least two quantification methods). At this more stringent level, 19 genes were differentially expressed at ZT3 and 13 at ZT15, while 4 genes were affected at both time points (Figure 2A). The majority of genes (17 of 28) showed a decrease in expression in *Zfhx3*$^{Sci/+}$ (Figure 2B). Interestingly, the expression of a number of circadian-related neuropeptides was decreased in *Zfhx3*$^{Sci/+}$ SCN, including *Vip*, its receptor (*Vipr2*), and prokineticin receptor 2 (*Prokr2*) (Figures 2C–2E). We conducted gene ontology (GO) enrichment analysis using all of the differentially expressed genes ($q < 0.05$ in at least one quantification method, n = 169) and found significant enrichment for a number of GO terms including neuron differentiation, regulation of cellular metabolic process, and regulation of cell proliferation (Table S2).

The limited work investigating this transcription factor to date has revealed a number of its gene targets (Berry et al., 2001; Mori et al., 2007; Qi et al., 2008; Yasuda et al., 1994). Using a phylogenetic shadowing-based approach (Bailey and Elkan, 1994), we constructed a motif-binding model for ZFHX3 based on the characterized binding motifs in these promoters (see Supplemental Experimental Procedures for more details) (Figure 3A). We then investigated whether this consensus AT motif, as well as the canonical circadian E-box, D-box, and RRE, were present in sequences upstream of the genes differentially regulated by ZFHX3 (Pscan score > 0.88). We found that the AT motif was present in promoter sequences from 39% of the differentially expressed genes, while the circadian motifs E-box, D-box, and RRE were absent (Figure 3B).

Network Analysis of *Zfhx3*$^{Sci/+}$ *SCN* Gene Expression Reveals Functionally Distinct Modules

To investigate whether any biological pathways were specifically altered in *Zfhx3*$^{Sci/+}$ SCN, we conducted further network analysis using the genes that were differentially expressed ($q < 0.05$ in at least one quantification method). More specifically, we used the Search Tool for the Retrieval of Interacting Genes/Proteins (STRING) to construct a protein interaction network. In order to characterize this network, we used a molecular complex detection algorithm (MCODE; Bader and Hogue, 2003). MCODE identified four modules, where module 1 achieved the highest ranked score (10.1, i.e., greatest connectivity), compared to module 2, which received a score of 5.5. Module 1 contained genes associated with neuropeptide function, whereas module 2 contained genes related to ribosome binding (Figure 3B), confirmed by GO analysis for both module 1 (Figure 3C; Table S3) and module 2 (Table S4).

Downregulation of the Neuropeptide-Rich Module in *Zfhx3*$^{Sci/+}$ SCN

A comparison of module 1 elements in *Zfhx3*$^{Sci/+}$ and *Zfhx3*$^{+/+}$ SCN revealed that 15 of 21 genes showed decreased expression in mutants. This included a number of neuropeptides and their receptors, such as: arginine vasopressin (*Avp*), gastrin-releasing peptide (*Grp*), *Nms*, prokineticin 2 (*Prok2*), *Prokr2*, *Vip*, and *Vipr2*. We measured their SCN mRNA expression at six time points across the light:dark cycle, using qPCR. *Grp*, *Vip*, and *Vipr2* had damped mRNA expression in *Zfhx3*$^{Sci/+}$ SCN (two-way ANOVA, genotype main effect, $p < 0.05$). Expression of *Grp* and *Vip*, two neuropeptides previously shown to be important for regulating the firing patterns of SCN neurons (Aton et al., 2005; Brown et al., 2005; Maywood et al., 2006;), was decreased but not absent at most time points (Figures 4A and 4B). It should be noted that *Zfhx3* expression is not cyclic in the SCN, with no significant differences in mRNA expression (two-way ANOVA, time main effect, $p > 0.2$) and no gross differences in protein localization within the SCN between *Zfhx3*$^{Sci/+}$ and *Zfhx3*$^{+/+}$ animals (Figures 4C and 4D).

Using immunofluorescence, we compared AVP, GRP, VIP, and VIPR2 levels in *Zfhx3*$^{Sci/+}$ and *Zfhx3*$^{+/+}$ SCN. We found that GRP and VIP levels, but not AVP and VIPR2 levels, were significantly decreased in *Zfhx3*$^{Sci/+}$ at ZT6 (Figures 4E–4I). There was a small but significant decrease in *Zfhx3*$^{Sci/+}$ SCN size compared to *Zfhx3*$^{+/+}$ (*Zfhx3*$^{Sci/+}$ SCN size: 53,600 ± 2,400 μm^2, n = 4; *Zfhx3*$^{+/+}$ SCN size: 66,900 ± 1,600 μm^2, n = 4; $p < 0.05$, t test, area of DAPI signal fluorescence). The data suggest that ZFHX3 actively influences the expression of these clock-relevant neuropeptides in the adult SCN and that this activation is disrupted in *Zfhx3*$^{Sci/+}$ mice. These differences were not primarily due to aberrant terminal differentiation, as there was no significant reduction in the number of AVP or VIP immuno-positive neurons (Figures 4J and 4K). Interestingly, ZFHX3 immuno-positive neurons almost completely overlapped with those for both AVP and VIP (Figure S3).

ZFHX3^{Sci} Has a Diminished Ability to Activate Transcription via the Circadian AT Motif

In order to investigate whether ZFHX3 might actively regulate module 1 genes in the adult SCN, we searched for strongly conserved consensus AT-motif, E-box, D-box, and RRE sequences within this module (Figure 3B). While none of the genes in the module had strongly conserved E-box, D-box, or RRE sequences, 7 of 15 genes showing damped oscillations in adult *Zfhx3*$^{Sci/+}$ SCN had a predicted AT motif (motif conservation score ≥ 0.88) within 0.5 kb of the transcription start site (TSS). This finding suggests that the motif may play a specific role in regulating the neuropeptide network and reveals a mechanism in regulating neural rhythms within the SCN.

To test whether the *Sci* mutation affects the ability of ZFHX3 to regulate transcription via the AT consensus motif, we cloned this motif (×7) into the pGL3-Enhancer Luciferase Reporter Vector.

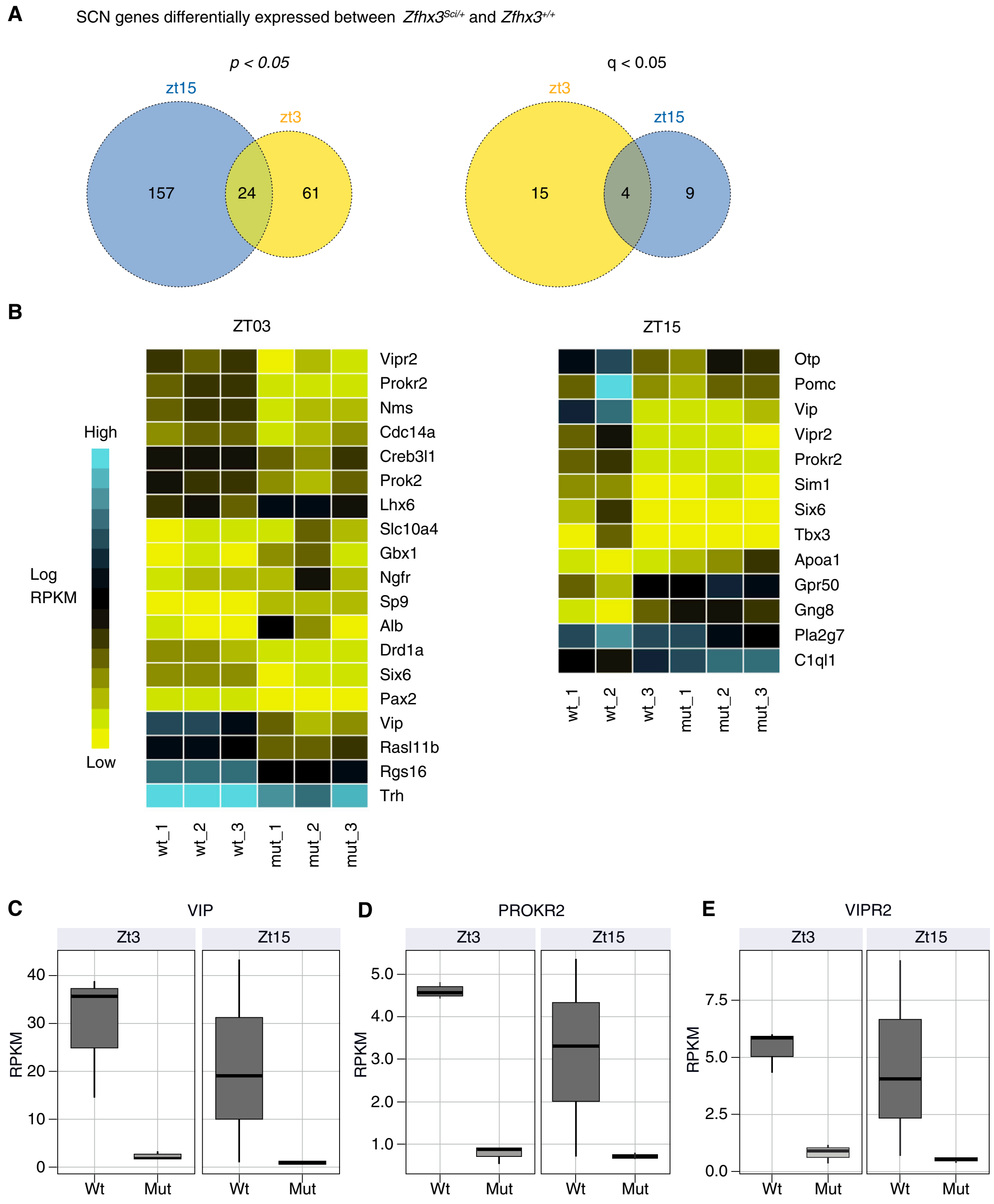

Figure 2. RNA Sequencing Reveals Transcripts Differentially Expressed in *Zfhx3*$^{Sci/+}$ SCN

(A) Venn diagrams depicting the number of differentially regulated transcripts, at ZT3 and ZT15, with a $\log_2$ fold change of >1 between *Zfhx3*$^{Sci/+}$ and *Zfhx3*$^{Sci/+}$ SCN. Genes were considered differentially expressed if they passed significance (left, $p < 0.05$; right, $q < 0.05$) for at least one of the three analysis methods (EdgeR, DESeq, or Cufflinks).

(B) Transcripts with significant q values at each time point were used to populate heatmaps. For each time point, a heatmap shows the average expression in reads per kilobase per million (RPKM) for each transcript. wt, wild-type; mut, mutant.

(C–E) Representative box plots of gene expression for (C) *Vip*, (D) *Prokr2*, and (E) *Vipr2* ($q < 0.05$).

See also Tables S1 and S2.

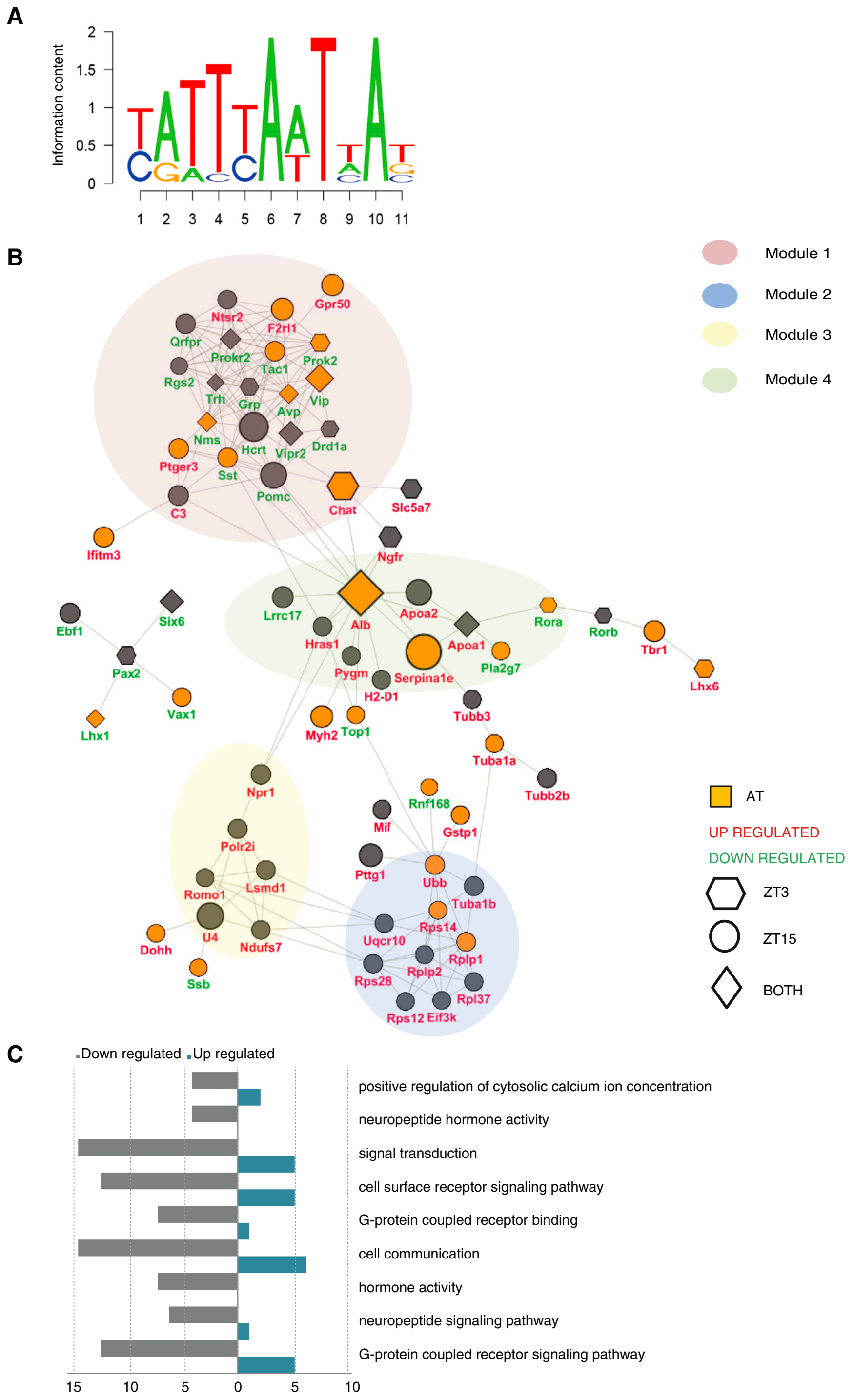

Figure 3. Functional Modules Determined for *Zfhx3*$^{Sci/+}$ SCN Expression

(A) The consensus AT motif derived from published AT sequences of genes regulated by *Zfhx3* (see Experimental Procedures).

(B) A protein-protein interaction network representing the differentially expressed transcripts in *Zfhx3*$^{Sci/+}$ SCN for all modules. Clustering analysis compartmentalized the network into modules of densely connected nodes. Positive identification of the consensus AT motif within the promoter regions of these transcripts is denoted by yellow shading. Node shape denotes the significance at different time points. Module 1 contains the greatest number of downregulated genes, AT motifs, and connectivity compared to other modules.

(C) The top nine overrepresented GO terms for genes in module 1. The number downregulated (gray) and number upregulated (blue) are listed for each term. Neuropeptide and signaling activities are among the top functional roles of the genes in this module.

See also Tables S3 and S4.

This reporter was then co-transfected with an expression vector containing recombinant *Zfhx3*, with or without the *Sci* mutation (*Zfhx3*^{Sci} and *Zfhx3*$^{+}$, respectively), into HEK293 cells. We assayed transcriptional activity in cell lysates using the dual luciferase assay and found that *Zfhx3*$^{+}$ showed an increased activation relative to the empty vector. In contrast, activation by *Zfhx3*^{Sci} was no different from that of the empty vector, supporting our hypothesis that the *Sci* mutation results in a decreased ability of ZFHX3 to activate transcription via the AT motif ($p < 0.05$, t test) (Figure 5A). We confirmed that the AT reporter response was specific to ZFHX3, as activation was at least three times higher than that of DBP or CLOCK/BMAL ($p < 0.05$, t test) (Figure S4A).

AT Motif Reporter Oscillation Is Circadian and Is Damped in *Zfhx3*$^{Sci/+}$ SCN

We explored whether similar deficits in AT-motif activation might underlie the *Zfhx3*$^{Sci/+}$ phenotype in ex vivo SCN. To investigate this, we generated lentiviral vectors (LVs) encoding a luciferase reporter driven by the AT motif (×7) to transduce slices. Transcription from the AT motif was active and showed a distinct circadian oscillation in the SCN (period = 24.5 ± 0.1 hr; n = 5) (Figure 5B). To test the specificity of the AT motif, we mutated its three most conserved residues (residues 6, 8, and 10), and generated LV-driving luciferase expression from these mutated sequences. Amplitude and robustness (as assessed by relative amplitude error and period error) of the oscillations driven by these "defective" sequences in the SCN were strongly reduced ($p < 0.05$, t test) (Figures 5B and 5C; Figures S4B–S4D), consistent with the specificity of AT-motif-mediated transcription.

We then compared AT-motif-driven luciferase expression in SCN slices from *Zfhx3*$^{+/+}$ and *Zfhx3*$^{Sci/+}$ animals. We found a

65% decrease in amplitude of the circadian oscillation of the AT motif in *Zfhx3*$^{Sci/+}$ SCN ($p < 0.05$, t test) (Figure 5D). Moreover, consistent with the amplitude and period shortening of PER2::LUC oscillations in *Zfhx3*$^{Sci/+}$ SCN slices, both the amplitude and period of AT-motif-driven luciferase oscillations were reduced when compared to *Zfhx3*$^{+/+}$ (period for *Zfhx3*$^{Sci/+}$: 24.1 ± 0.1 hr; n = 8; period for *Zfhx3*$^{+/+}$: 24.5 ± 0.1 hr; n = 8; $p < 0.05$, t test) (Figures 5E and 5F).

To determine whether the circadian oscillation of AT activation may be sensitive to the TTFL, we treated SCN with PF670462, an inhibitor of casein kinase 1 delta/epsilon that is known to slow down the TTFL by delaying degradation of PER proteins in the SCN (Meng et al., 2010). Before treatment, transduced SCN showed clear circadian oscillations of AT-driven bioluminescence (Figure S4E). On treatment with vehicle, period did not change, whereas on addition of PF670462, there was a significant period lengthening, which was completely reversible on washout (Figure S4F). Thus, the circadian oscillation of the ZFHX3/AT axis may be sensitive to the core TTFL and, in turn, acts as an output transducer of the circadian signal to its respective target genes.

Ex Vivo or In Vivo Knockdown of *Zfhx3* SCN Expression Lengthens Circadian Period

To investigate the nature of the *Sci* mutation further and to establish a sustained regulatory role for *Zfhx3* in postnatal and adult SCN, we used two complementary approaches to knock down *Zfhx3*. In the first, we used in vivo RNAi to knock down in the adult SCN of C57Bl/6 mice. SiRNA was administered via stereotaxic injection into the third ventricle adjacent to the SCN to mice on a 12:12 light:dark cycle. One day after injection, mice were placed in constant darkness (DD) for 8 days to measure τ_{DD}. Surprisingly, animals injected with siZfhx3 had a significantly lengthened τ_{DD} (23.74 ± 0.05 hr), compared to control siRNA-injected animals (23.46 ± 0.05 hr), $p < 0.05$, t test (Figures 5G–5I). Injection of siZfhx3 resulted in 43% downregulation of *Zfhx3* mRNA levels ($p < 0.05$, t test) (Figure 5J).

In a second experiment, we compared periods of AT-motif-driven molecular oscillations in ex vivo SCN slices using a previously described *Zfhx3*^{Flox} line (Sun et al., 2012). We compared AT-motif oscillations in *Zfhx3*$^{+/+}$ and the *Zfhx3*$^{Flox/+}$ SCN before and after transduction with Syn-CRE viral vectors. Treatment with Syn-CRE caused a 49% decrease in expression of the full-length *Zfhx3* transcript in the SCN *Zfhx3*$^{Flox/+}$ mice ($p < 0.05$) and resulted in an ~1.5-hr period lengthening (25.85 ± 0.37 hr versus 23.86 ± 0.20 hr, $p < 0.05$), while no lengthening was evident in controls (25.19 ± 0.47 hr versus 24.67 ± 0.51 hr) (Figures 5K and 5L), consistent with the in vivo siRNA experiment. Both in vivo and ex vivo findings confirm a sustained role for ZFHX3 in regulating circadian period in adult and postnatal SCN, and they additionally suggest that the *Sci* mutation has a dominant-negative, rather than a hypomorphic, effect on circadian period.

ZFHX3 Directly Interacts with the AVP and VIP Promoters

To confirm that ZFHX3 binds to the promoter of the key predicted target genes *Avp* and *Vip* in vivo, we performed quantitative ChIP on SCN tissue from *Zfhx3*$^{+/+}$ animals (ZT3) using ZFHX3 antiserum. We found a significant increase in immunoprecipitated DNA upstream of the transcriptional start site (TSS) for both *Avp* and *Vip* compared to the *Gapdh* control promoter region, suggesting that ZFHX3 binds to both target gene promoters (Figures 6A and 6B). At the *Vip* promoter, this binding occurred in the vicinity of the AT motif to a far greater extent than in an adjacent upstream region. These data strongly suggest that ZFHX3 binds to *Avp* and *Vip* promoters in close proximity to AT consensus motifs.

Failure to Activate Neuropeptide Promoter AT Motifs Underlies the *Zfhx3*^{Sci} Phenotype

As several genes downregulated in module 1 had predicted AT motifs upstream of their TSSs (Figure 3B), we hypothesized that their diminished expression may be due to a decreased ability of ZFHX3^{Sci} to activate transcription via the AT motif. To test this, we cloned the module 1 gene putative promoters with strong predicted AT motifs (*Avp*, *Vip*; Pscan threshold > 0.85), with moderate predicted motifs (*Grp*, *Prokr2*; Pscan threshold > 0.80), or without one (*Drd1a*, *Vipr2*; Pscan threshold ≤0.8) into the pGL3-Enhancer Luciferase Reporter Vector. We co-transfected these reporters together with the *Zfhx3*^{Sci} or *Zfhx3*$^{+}$ expression vectors into HEK293 cells. In both cases, there was increased transcriptional activation of reporters containing strong predicted AT motifs when we overexpressed ZFHX3^{+}, while this activation was dramatically less when we overexpressed ZFHX3^{Sci} (Figures 6C and 6D). Furthermore, ZFHX3^{+}-driven expression was significantly higher than that driven by DBP or by CLOCK/BMAL (Figures 6E and 6F). Co-transfection of reporters containing moderate predicted AT motifs with ZFHX3^{+} led to increased activation of the *Prokr2* reporter (Figure 6G; Figure S5A) but not the *Grp* reporter. Finally, we found no significant activation of those reporters without predicted AT motifs (Figures S5B and S5C).

To further investigate the role of the AT motif in ZFHX3-dependent transcription, we mutated the three most conserved residues (residues 6, 8, and 10) within the *Avp* and *Vip* promoter constructs. These constructs without AT motifs had significantly decreased levels of transcriptional activation (Figures 6H and 6I) ($p < 0.05$, t test). Our findings strongly support a role for ZFHX3-driven, AT-motif-mediated transcriptional activation in the maintenance and regulation of SCN neuropeptide expression. Furthermore, the short-period circadian phenotype evident both in vivo and ex vivo *Zfhx3*$^{Sci/+}$ is likely mediated by a failure of ZFHX3^{Sci} to drive these oscillations effectively.

In addition to the neuropeptide genes identified in the RNA-sequencing analysis, circadian genes *Cry2*, *Per1*, and *Per2* have a predicted AT motif in their promoters, whereas *Cry1* does not (Figure S5D). Using a similar co-transfection strategy, we found a small but significant activation of the all three promoters with the predicted AT motif by ZFHX3^{+} but not for the *Cry1* promoter (Figures S5E–S5H). As all four of these promoters contain E-box sequences, these data suggest that the AT motif, not the E-box, mediates this ZFHX3^{+} activation.

DISCUSSION

The identification of circadian genes has advanced through the use of a number of approaches, including phylogenetic

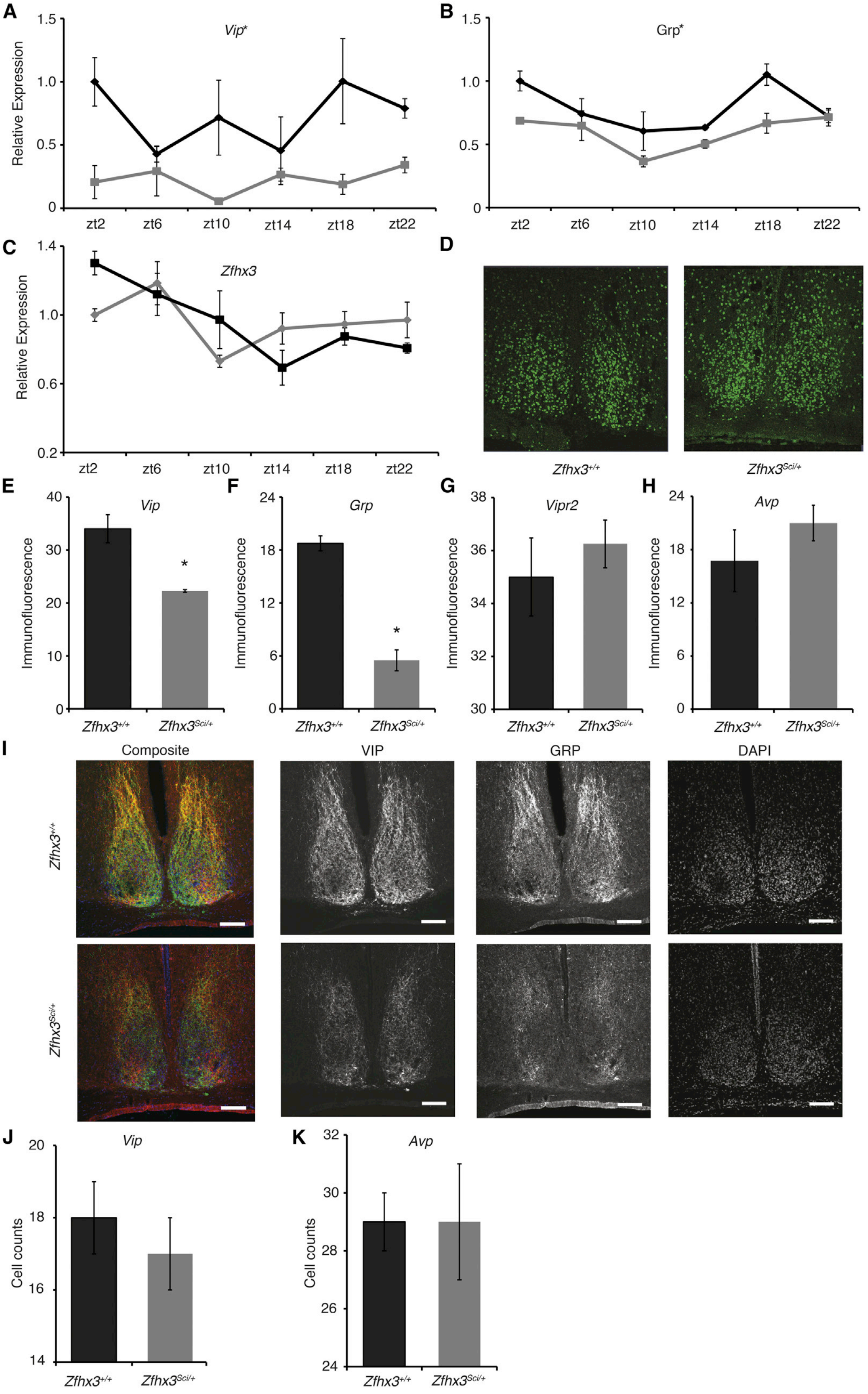

(legend on next page)

approaches, ENU mutagenesis screens, in silico analysis, and cell reporter screening methods. Identification of the *Zfhx3*$^{Sci/+}$ mutant underlines the utility of mutagenesis approaches in discovering circadian phenotype/genotype associations in mammals (Vitaterna et al., 1994; Godinho et al., 2007). As with cell reporter screening methods, ENU mutagenesis has yielded molecular components that can modulate the cell-autonomous TTFL. However, as ENU mutagenesis screens assay in vivo phenotypes, they can uniquely detect mutants, such as *Sci*, that affect the SCN intercellular network. Furthermore, the ability of ENU to generate series of mutant alleles enables the discovery of different phenotypes from those detected by loss-of-function studies. This is evident in the opposing effects of *Zfhx3* siRNA knockdown and the *Zfhx3*$^{Sci/+}$ mutant on $\tau_{DD,}$ suggesting that the *Zfhx3*$^{Sci/+}$ mutant is not a hypomorphic allele and, instead, is a gain-of-function, possibly dominant-negative, allele. This highlights the continued value of using ENU mutagenesis, in conjunction with other methods, in circadian gene discovery.

We report that the short-period phenotype of *Sci* is caused by a mutation in *Zfhx3*, a gene highly expressed in adult SCN. We also found that the mutant protein ZFHX3^{Sci} has a decreased ability to transcriptionally activate an AT motif, a circadian motif present in the promoters of a class of neuropeptides, among other genes, that are critical for synchronous cellular rhythms of electrical firing and molecular cycling in the SCN (Maywood et al., 2006, 2011; Aton and Herzog, 2005). Furthermore, we show that activation of AT-dependent transcription follows a clear circadian cycle in the SCN, that the rhythm is sensitive to ZFHX3 function, and that it may also be downstream of the core TTFL. These results suggest a role for ZFHX3 beyond the core TTFL in modulating SCN free-running period in a cell-nonautonomous fashion, possibly by regulation of a neuropeptide network that governs the intercellular synchrony of the individual neuronal clocks in the adult SCN (Liu et al., 2007). We have provided evidence that ZFHX3 activates a clock-regulated transcriptional axis, thereby expanding the known chorus of SCN regulatory proteins responsible for the maintenance of mammalian circadian rhythms.

We provide evidence that a consensus AT motif can be transcriptionally activated in a circadian fashion in the SCN. Previous studies found that ZFHX3 can either positively (Qi et al., 2008) or negatively (Mori et al., 2007; Yasuda et al., 1994) regulate gene expression via AT motifs, depending on the functional domains and binding partners involved (Berry et al., 2001; Sakata et al., 2014). Our data show that ZFHX3 can positively regulate the expression of certain neuropeptides via a direct and AT-motif-dependent interaction with their promoters, and that this regulation is disrupted in *Zfhx3*$^{Sci/+}$ mice. The ZFHX3-regulated AT motif now extends the list of DNA motifs managing the SCN circadian transcriptional landscape, thereby controlling circadian behavior (Bozek et al., 2009, 2010; Koike et al., 2012).

We have shown that the sequence specificity of the AT motif is critical for regulating the amplitude and robustness of its rhythmic activation. Furthermore, all measures were deficient in SCN slices from *Zfhx3*$^{Sci/+}$ animals, supporting the case that ZFHX3 is a transregulator of this motif. It should be noted that, because *Zfhx3* mRNA expression does not strongly cycle within the SCN, there may be other circadian-regulated genes that act with ZFHX3 to activate the AT motif. This is not unprecedented, particularly within the SCN TTFL, as neither *Clock* mRNA nor its protein expression oscillate, but it still plays a key circadian role (Lee et al., 2001). Further exploration of ZFHX3 binding partners, particularly those that are circadian regulated, will contribute to our understanding of its role in adult SCN rhythmic oscillations.

It is clear from our results that the period of AT-driven transcription is sensitive to the period of the TTFL, as evidenced by the effect of CK1 inhibition. This argues that the ZFHX3/AT axis is downstream of the TTFL. However, it is also affected by the *Zfhx3*^{Sci} mutation, as is the period of the TTFL, as reported by PER expression. The TTFL and AT axes are, therefore, reciprocally dependent, each able to influence the other. We propose that this arises from the circuit-level effects of AT-driven transcription, specifically of neuropeptide-encoding genes, leading to a logical module in which the TTFL affects AT function. In turn, AT motif activation affects neuropeptidergic expression, leading to cell-nonautonomous effects on circuit-level signaling in the SCN, and ultimately influences TTFL activity (Figure 7).

The S*ci* mutation appears to predominantly influence circadian rhythmicity in a cell-nonautonomous manner by directly regulating the expression of specific neuropeptides that, in turn, synchronize local networks within the SCN. Nevertheless, we cannot rule out the possibility that this obscures a smaller cell-autonomous effect of *Sci*. Unlike many core clock genes, *Zfhx3* is not ubiquitously expressed, so any cell-autonomous influence would be limited to regions where it is expressed, such as the SCN. In keeping with this, we found a significant, albeit small, effect on the period of *Per2*-promoter-driven oscillation in both ex vivo SCN slices and in vitro overexpression assays. Although a previous large-scale siRNA screen failed to find significant effects of two Zfhx3 siRNAs on period length in U20S

Figure 4. Significant Decreases in Neuropeptide Expression Detected in *Zfhx3*$^{Sci/+}$ SCN

(A and B) mRNA expression of both (A) *Vip* and (B) *Grp* was significantly decreased in SCN of *Zfhx3*$^{Sci/+}$ (gray lines) compared to *Zfhx3*$^{+/+}$ (black lines) at multiple time points (n = 4). $p < 0.05$, ANOVA.

(C) *Zfhx3* mRNA expression in SCN is stable throughout the day and does not significantly differ by genotype (n = 4, gray and black lines represent *Zfhx3*$^{Sci/+}$ and *Zfhx3*$^{+/+}$ respectively).

(D) ZFHX3 protein localization does not grossly differ across genotype at ZT6.

(E and F) As shown here, (E) VIP and (F) GRP immunofluorescence in SCN was decreased in *Zfhx3*$^{Sci/+}$ animals ($p < 0.05$).

(G and H) As shown here, (G) VIPR2 and (H) AVP SCN protein expression was not different between genotypes ($p > 0.1$).

(I) Confocal images (20× magnification) of immunostaining for VIP, GRP, and DAPI and the composite image in the SCN of *Zfhx3*$^{+/+}$ (top panel) and *Zfhx3*$^{Sci/+}$ (bottom panel). Scale bars, 100 μm.

(J and K) The number of (J) VIP- and (K) AVP-immunopositive cells was quantified, with no significant differences detected across genotype.

Error bars indicate SEM. *$p < 0.05$, t test.

See also Figure S3.

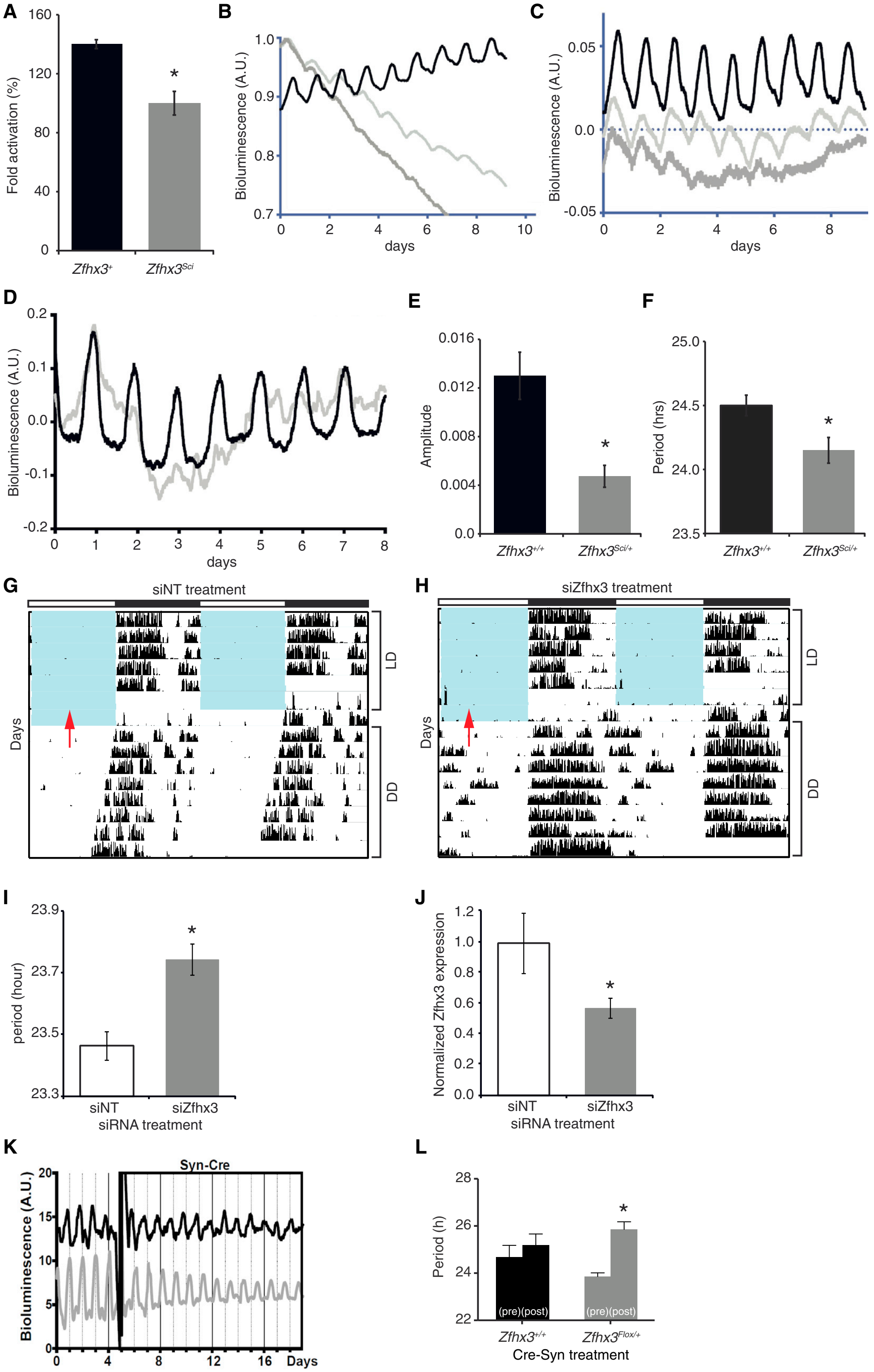

(legend on next page)

cells, both siRNAs did lengthen the period (test siRNAs: 25.99 ± 0.43 hr and 25.83 ± 0.21 hr; control: 25.20 ± 0.55 hr, mean ± SD) (Zhang et al., 2009), a trend consistent with the significant effects reported here. Further studies investigating the effects of *Sci* on circadian reporter oscillations in individual ZFHX3-positive cells would further elucidate the nature of any small, cell-autonomous role for *Sci*.

Our results indicating that mutant ZFHX3 directly influences the expression of neuropeptides in adult SCN are consistent with this model. As the intercellular coupling driven by neuropeptides synchronizes intracellular molecular clockworks of individual neurons (Maywood et al., 2006, 2011; Liu et al., 2007), misregulation of these neuropeptides will provoke disruptions in circadian parameters. This disruption in *Zfhx3*$^{Sci/+}$ mice may underlie the decreased free-running period and amplitude seen for locomotor activity in vivo and PER2::LUC oscillations in ex vivo SCN. The larger distributions of cellular period length and phase seen in individual neurons in Zfhx3$^{Sci/+}$ SCN cultures support this disruption of critical neuropeptides. Similarly, features of the Zfhx3$^{Sci/+}$ phenotype have been reported in neuropeptidergic mutants. Mice lacking VIP either are arrhythmic or have a shorter period and decreased amplitude compared to controls (Aton et al., 2005; Brown et al., 2007). There is also a decrease in the amplitude of locomotor activity in both *Prok2*- and *Prokr2*-deficient mice (Prosser et al., 2007; Li et al., 2006). Ultimately, these findings suggest that decreased transcriptional activation of the neuropeptide complex seen in *Zfhx3*$^{Sci/+}$ mice may account for its circadian phenotype. The circadian activation seen for the AT motif in SCN slices was mirrored by the rhythmic expression of *Grp*, but not *Vip*, mRNA in SCN. As *Vip* expression is likely regulated by light stimulation (Shinohara et al., 1993), this discrepancy may be due to differences in light input between intact SCN in vivo and SCN slices. Recent studies have additionally suggested that both AVP and VIP signaling play a role in entrainment (An et al., 2013: Yamaguchi et al., 2013). The *Zfhx3*$^{Sci/+}$ mutant also shows deficits in entrainment under various conditions (Bacon et al., 2004). As pre-treatment with VIP facilitates re-entrainment (An et al., 2013), the decreases in VIP expression in *Zfhx3*$^{Sci/+}$ SCN may partially underlie these deficits in entrainment in *Zfhx3*$^{Sci/+}$ mice.

We found that knockdown of *Zfhx3* in the SCN of wild-type mice results in an altered period, which suggests that *Zfhx3* has a function in adult brain, aside from its developmental role (Ishii et al., 2003). Nevertheless, we considered whether the *Zfhx3*$^{Sci/+}$ circadian phenotype is due partially to differences in terminal differentiation of SCN peptidergic cells, as reported recently for *Lhx1* mutants (Bedont et al., 2014; Hatori et al., 2014). We found no difference in the number of neuropeptide immunopositive cells in adult SCN; thus, there is no direct evidence that a deficit in terminal differentiation explains the circadian deficit in *Zfhx3*$^{Sci/+}$. These observations imply that ZFHX3 regulates neuropeptide levels in adult SCN independently of its roles in developing tissue. The sustained expression of ZFHX3 in adult SCN (Lein et al., 2007; VanDunk et al., 2011) is further compatible with its playing such a role. *Zfhx3* is not the first circadian gene to be shown to play this dual role. The *Rora* mutant *staggerer*, for example, has independent roles in both cerebellum development and circadian regulation in adults (Gold et al., 2007). Given that the SCN in adult *Zfhx3*$^{Sci/+}$ mice is slightly smaller, we cannot rule out the possibility that developmental changes may contribute to the adult circadian phenotype. Defining the adult versus embryonic roles for *Zfhx3* will be resolved by investigating various regional and stage-specific conditional *Zfhx3* knockout mice.

Our initial finding that *Zfhx3*^{Sci} fails to drive expression via the AT motif in vitro suggested to us that knockdown of *Zfhx3* would lead to a similar shortened period in vivo. The apparent lack of congruency between *Zfhx3*^{Sci} and *Zfhx3* knockdown effects in vivo could be related to a number of factors. In the first instance, the results of RNA sequencing confirm that many genes in SCN are either positively or negatively regulated by ZFHX3. The integrated effects of mutant and wild-type ZFHX3 in vivo need not necessarily solely reflect the outcome of in vitro studies at *Avp* and *Vip* promoters. Furthermore, previous literature confirms both positive and negative regulatory roles for ZFHX3 and that activation state is dependent on the expression of additional cofactors and the interactions of ZFHX3 with these cofactors (Qi et al., 2008; Mori et al., 2007; Yasuda et al., 1994). Although we have yet to explore this, mutant ZFHX3 may be interfering with these interactions. These observations would support our interpretation that *Sci* is a dominant-negative mutation. Allelic mutations with opposing effects on period length are not unprecedented in circadian biology as evidenced, for

Figure 5. ZFHX3^{SCI} Differentially Activates a Circadian Motif in SCN

(A) In vitro activation of the AT motif by overexpressing *Zfhx3* without and with the *Sci* mutation (*Zfhx3*$^{+}$ and *Zfhx3*^{Sci} respectively) using a luciferase reporter construct driven by the AT motif (×7) in HEK293 cells. *Zfhx3*$^{+}$ transcriptionally activated the AT motif, while *Zfhx3*^{Sci} did not ($p < 0.05$, t test).

(B and C) As shown, (B) raw data and (C) de-trended data show circadian activation of AT sequences in SCN slices transduced by LVs coding for the luciferase reporter driven by the AT motif. A.U., arbitrary units.

(D) AT-motif-driven luciferase expression in SCN slices from *Zfhx3*$^{Sci/+}$ (gray lines) and *Zfhx3*$^{+/+}$ (black lines) mice.

(E and F) There was a substantial decrease in (E) the amplitude of AT motif activation in *Zfhx3*$^{Sci/+}$ and a small, but significant decrease in (F) period compared to *Zfhx3*$^{+/+}$ ($p < 0.05$, t test).

(G and H) Representative double-plotted actograms of wheel-running activity in C57Bl/6 mice injected with (G) control siRNA (siNT) or (H) siZfhx3 (arrow denotes time of injection). Blue shading represents periods when lights are on. Vertical black bars represent wheel running activity. LD, light:dark cycle.

(I) Animals injected with siZfhx3 had a significantly lengthened τ_{DD} (23.74 ± 0.05 hr, mean ± SEM) compared to control siRNA (23.46 ± 0.05 hr). $p < 0.05$, t test.

(J) Injection of siZfhx3 led to a 43% downregulation of *Zfhx3* mRNA levels compared to control siRNA ($p < 0.05$, t test).

(K and L) In (K), representative plots are shown of AT activation before and after transduction with the Syn-CRE vector in ex vivo SCN of *Zfhx3*$^{+/+}$ (black lines) and *Zfhx3*$^{Flox/+}$ mice (gray lines). (L) *Zfhx3* deletion in *Zfhx3*$^{Flox/+}$ SCN significantly lengthened the period of AT activation relative to Zfhx3$^{+/+}$.

Error bars indicate SEM. *$p < 0.05$, t test.

See also Figure S4.

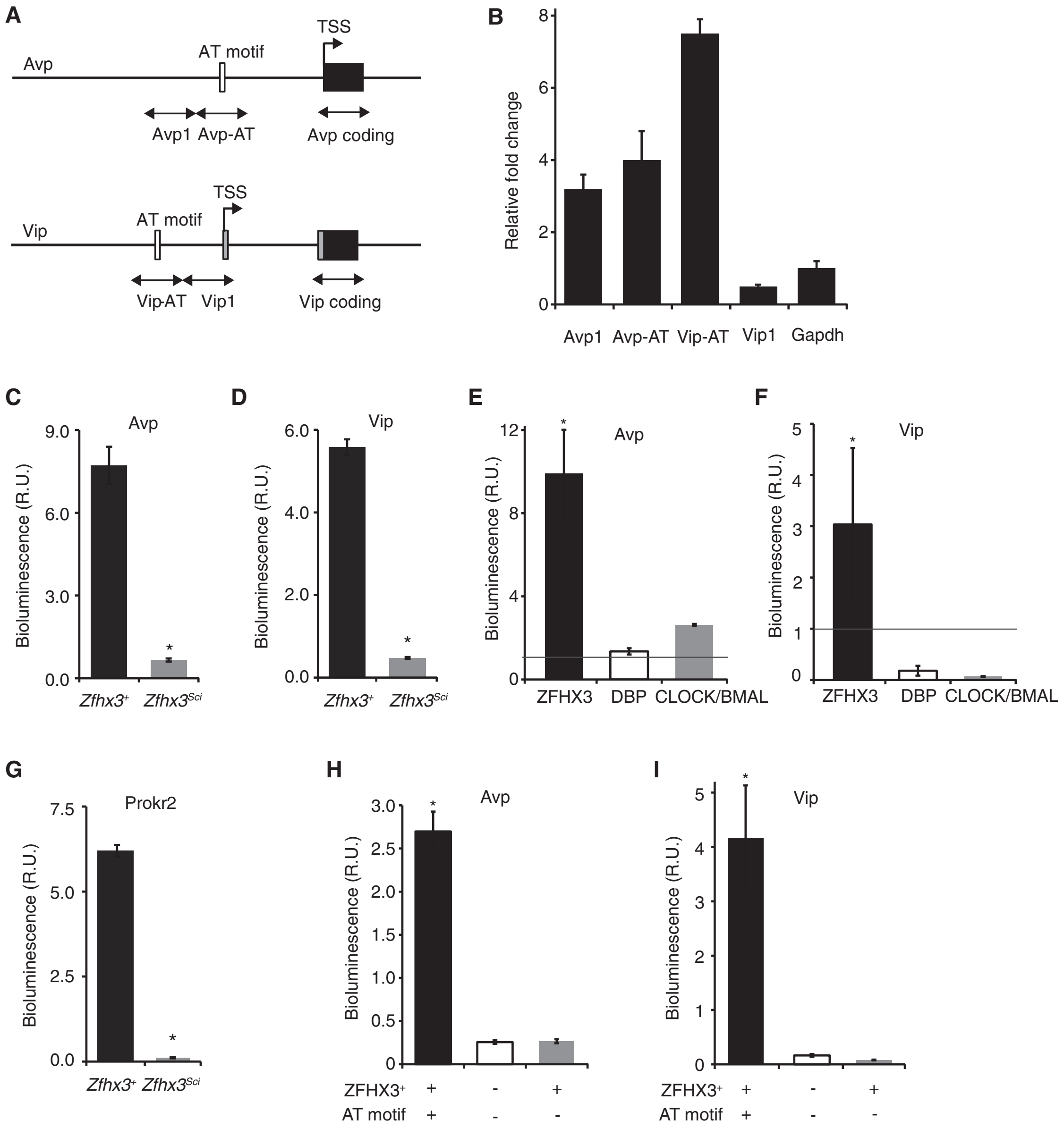

Figure 6. ZFHX3^{SCI} Interacts with and Differentially Activates the AT Motif in *Avp* and *Vip* Promoters

Quantitative ChIP of *Zfhx3*$^{+/+}$ SCN tissue samples using ZFHX3 antiserum.

(A) Primer pairs were designed to span the AT motif (Avp-AT/Vip-AT) and an adjacent region (Avp-1/Vip-1) upstream of the TSS (primers in the coding region of each gene were used to normalize each reaction).

(B) Zfhx3 binds to the promoter around the AT motif of both *Avp* and *Vip* compared to the control gene, *Gapdh*. Data are shown as fold change normalized for input relative to the corresponding coding region of each gene and are taken at ZT3 (n = 3).

(C and D) Overexpression of ZFHX3 with the *Sci* mutation (ZFHX3^{Sci}) was ineffective in activation of luciferase driven by AT-motif-containing promoters of (C) *Avp* and (D) *Vip* in HEK293 cells ($p < 0.05$, t test). R.U., relative units.

(E and F) *Zfhx3*$^{+}$ activation (black bars) of the (E) *Avp* and (F) *Vip* promoters was more than three times that seen for either DBP (white bars) or CLOCK/BMAL (gray bars) ($p < 0.05$, t test).

(G) Overexpression ZFHX3^{Sci} was also ineffective in activation of the *Prokr2* promoter *Prokr2* in HEK293 cells ($p < 0.05$, t test).

(H and I) Mutation of the three most conserved residues (positions 6, 8, and 10) in the AT motifs of both the (H) *Avp* and (I) *Vip* promoter constructs (gray bars) resulted in significantly decreased levels of ZFHX3+ activation compared to those with AT motifs intact (black bars) ($p < 0.05$, t test). White bars represent the activation of the mutated promoter constructs alone.

Error bars indicate SEM. *$p < 0.05$, t test.

See also Figure S5.

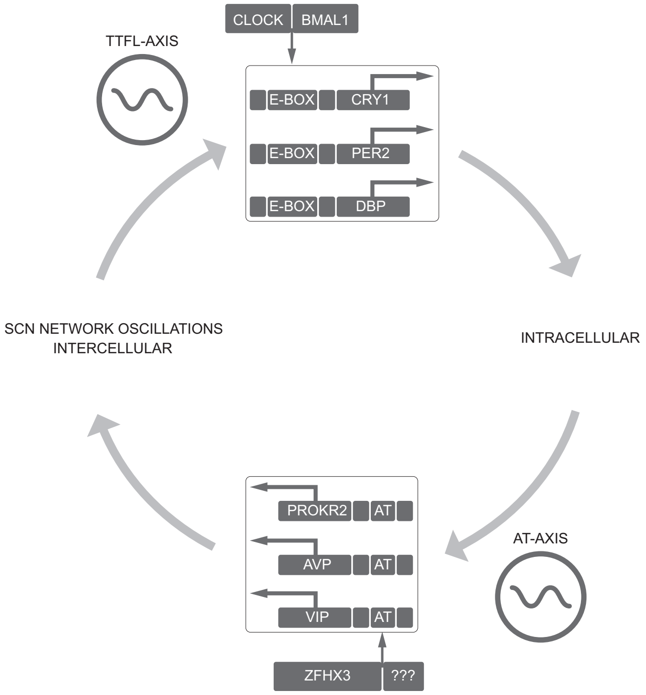

Figure 7. Model Depicting the Role of the ZFHX3/AT Axis in Maintaining SCN Oscillations

A diagram outlining the proposed mechanism for maintaining robust circadian oscillations in the SCN. The ZFHX3/AT axis is sensitive to the TTFL and, in turn, feeds back temporal information to the TTFL through its neuropeptidergic-dependent effects on intercellular, circuit-level signaling.

example, by *per* locus mutations in *Drosophila* (Konopka and Benzer, 1971).

Conclusions

We found that the circadian phenotype in *Sci* is caused by a mutation in *Zfhx3*, a transcription factor highly expressed in adult SCN. The mutant protein has a diminished ability to activate a consensus AT motif, leading to decreased expression of a class of neuropeptides critical for intercellular synchrony and rhythm amplitude in the SCN. Importantly, the absence of gross differences in peptidergic cell numbers in adult $Zfhx3^{Sci/+}$ SCN and the significant effects of gene knockdown postnatally suggest that this regulation occurs independently of any putative developmental effects of *Zfhx3*. Furthermore, we show that reporters driven by the consensus AT motif are activated in a circadian fashion in ex vivo SCN slices and that the rhythm of AT-driven transcription is sensitive to the $Zfhx3^{Sci/+}$ mutation and the TTFL of the core clockwork. Thus, the activation of regulatory AT motifs by direct interaction with ZFHX3 plays a significant role in maintaining the circadian transcriptional landscape of the SCN. Moreover, the ZFHX3/ AT axis sits within an unprecedented logical module within the SCN. Its function is sensitive to the TTFL and, in turn, because of its neuropeptidergic effects on circuit level signaling, it feeds back temporal information to the TTFL. This module, therefore, encompasses cell-autonomous and circuit-level circadian pacemaking, incorporating them into a coherent oscillation to drive circadian behavior.

EXPERIMENTAL PROCEDURES

Mice

All animal studies were performed under the guidance issued by the Medical Research Council in Responsibility in the Use of Animals for Medical Research (July 1993) and Home Office Project Licenses 30/2686 and 80/2310, with local ethical approval.

Determination of Zfhx3-Specific AT Motif

To construct a motif-binding model for ZFHX3, we searched the literature for previously identified binding sites (Figure S6). We used sequences from genes that are directly regulated through a characterized binding motif and generated the consensus sequence using a mixture model by multiple EM (expectation maximization) for motif elicitation (MEME) (Bailey and Elkan, 1994).

Ex Vivo SCN Slice Experiments

Brains were removed and sectioned as reported previously (Maywood et al., 2006). Bioluminescent emissions from PER2::LUC SCN and lung slices were recorded using photomultiplier tubes (PMT; Hamamatsu) and CCD cameras (Hamamatsu) as described previously (Maywood et al., 2013). Lentiviral transduction of SCN slices was performed as described previously (see Supplemental Experimental Procedures) (Brancaccio et al., 2013).

RNA Sequencing

RNA sequencing was conducted at the Oxford Genomics Centre (Wellcome Trust Centre for Human Genetics, University of Oxford). See Supplemental Experimental Procedures for more details, including SCN collection, RNA extraction, and qPCR validation.

RNA-Sequencing Analysis and Network Analysis

Whole transcriptome analysis was carried out using a custom-developed pipeline (see Supplemental Experimental Procedures).

Motif Analysis

The position frequency matrix of the ZFHX3 binding site, termed AT motif, was determined by MEME. Proximal enhancer regions (450 bp upstream and 50 bp downstream of the TSS) were searched for motif occurrence in the differentially expressed genes ($q < 0.05$) from the combined analysis (see RNA Sequencing section earlier). We identified the ZFHX3 binding site with the Pscan software (Zambelli et al., 2009), with a threshold of >0.88 defining positive hits.

Luciferase Reporter Gene Assays

We used the Dual-Luciferase Reporter Assay (Promega) to quantify the luciferase activity (see Supplemental Experimental Procedures for more details).

Immunofluorescence

Confocal microscopy was used to image immunofluorescence in free-floating brain sections (40 μm) using a range of antibodies and quantified using ImageJ software (Figure S7; Supplemental Experimental Procedures).

ACCESSION NUMBERS

The accession number for the Sci allele reported in this paper is MGI: 2679554.

SUPPLEMENTAL INFORMATION

Supplemental Information includes Supplemental Experimental Procedures, seven figures, and six tables and can be found with this article online at http://dx.doi.org/10.1016/j.cell.2015.06.060.

AUTHOR CONTRIBUTIONS

M.J.P. conducted the positional cloning, RNA extractions, the qPCR, and the in vitro reporter gene assays. R.S., S. Sethi, M.M.S., and A.-M.M. conducted the bioinformatic analysis for the RNA sequencing data. S. Saito provided

experimental materials. E.S.M. and M.H.H. conducted the ex vivo SCN slice work. M.B. performed lentiviral transductions of SCN slices and characterized the circadian oscillations of the AT motifs. E.S.M. and N.J.S. conducted the immunofluorescence. P.L.O. and M.J.F. conducted the ChIP experiments. A.J., Y.C., and R.G.F. planned and conducted the in vivo RNAi experiments. A.R.B., G.J., J.E.C., J.K.E., R.B., and S.W. all aided in the phenotyping, breeding, and management of the mouse colonies. C.E. validated the ZFHX3 antibody. P.M.N. developed the underlying scientific research project. M.H.H., M.J.P., M.B., E.S.M., and P.M.N. all aided in planning the experimental approach. All authors aided in writing the manuscript.

ACKNOWLEDGMENTS

P.M.N. and M.H.H. were supported by the MRC and by the 6th Framework Project EUCLOCK (No. 018741). R.S. was supported by an MRC Biomedical Informatics Fellowship. P.L.O. and M.J.F. were supported by a European Research Council Starting Grant "PAROSIN" awarded to P.L.O. A.J. was supported by a WT grant (090684/Z/09/Z). We thank Dr. Takahashi for generously providing the PER2::LUC knockin mice. We thank Dr. Filippo Tamanini for the CLOCK and BMAL expression vectors. We thank Dr. Jürgen Ripperger for generously providing the DBP expression vector. We thank the biomedical staff at the Mary Lyon Center, MRC Harwell, and the Ares Facility, Cambridge. Thanks to Steve Thomas for reformatting the figure images, and thanks to Helen Hilton for work validating the anti-ZFHX3 antibody.

Received: October 15, 2014
Revised: March 25, 2015
Accepted: June 1, 2015
Published: July 30, 2015

REFERENCES

Acevedo-Arozena, A., Wells, S., Potter, P., Kelly, M., Cox, R.D., and Brown, S.D. (2008). ENU mutagenesis, a way forward to understand gene function. Annu. Rev. Genomics Hum. Genet. *9*, 49–69.

Albers, H.E., Gillespie, C.F., Babagbemi, T.O., and Huhman, K.L. (1995). Analysis of the phase shifting effects of gastrin releasing peptide when microinjected into the suprachiasmatic region. Neurosci. Lett. *191*, 63–66.

Albrecht, U. (2012). Timing to perfection: the biology of central and peripheral circadian clocks. Neuron *74*, 246–260.

An, S., Harang, R., Meeker, K., Granados-Fuentes, D., Tsai, C.A., Mazuski, C., Kim, J., Doyle, F.J., 3rd, Petzold, L.R., and Herzog, E.D. (2013). A neuropeptide speeds circadian entrainment by reducing intercellular synchrony. Proc. Natl. Acad. Sci. USA *110*, E4355–E4361.

Aton, S.J., and Herzog, E.D. (2005). Come together, right . . . now: synchronization of rhythms in a mammalian circadian clock. Neuron *48*, 531–534.

Aton, S.J., Colwell, C.S., Harmar, A.J., Waschek, J., and Herzog, E.D. (2005). Vasoactive intestinal polypeptide mediates circadian rhythmicity and synchrony in mammalian clock neurons. Nat. Neurosci. *8*, 476–483.

Bacon, Y., Ooi, A., Kerr, S., Shaw-Andrews, L., Winchester, L., Breeds, S., Tymoska-Lalanne, Z., Clay, J., Greenfield, A.G., and Nolan, P.M. (2004). Screening for novel ENU-induced rhythm, entrainment and activity mutants. Genes Brain Behav. *3*, 196–205.

Bader, G.D., and Hogue, C.W. (2003). An automated method for finding molecular complexes in large protein interaction networks. BMC Bioinformatics *4*, 2.

Bailey, T.L., and Elkan, C. (1994). Fitting a mixture model by expectation maximization to discover motifs in biopolymers. Proc. Int. Conf. Intell. Syst. Mol. Biol. *2*, 28–36.

Bedont, J.L., LeGates, T.A., Slat, E.A., Byerly, M.S., Wang, H., Hu, J., Rupp, A.C., Qian, J., Wong, G.W., Herzog, E.D., et al. (2014). Lhx1 controls terminal differentiation and circadian function of the suprachiasmatic nucleus. Cell Rep. *7*, 609–622.

Berry, F.B., Miura, Y., Mihara, K., Kaspar, P., Sakata, N., Hashimoto-Tamaoki, T., and Tamaoki, T. (2001). Positive and negative regulation of myogenic differentiation of C2C12 cells by isoforms of the multiple homeodomain zinc finger transcription factor ATBF1. J. Biol. Chem. *276*, 25057–25065.

Bozek, K., Relógio, A., Kielbasa, S.M., Heine, M., Dame, C., Kramer, A., and Herzel, H. (2009). Regulation of clock-controlled genes in mammals. PLoS ONE *4*, e4882.

Bozek, K., Rosahl, A.L., Gaub, S., Lorenzen, S., and Herzel, H. (2010). Circadian transcription in liver. Biosystems *102*, 61–69.

Brancaccio, M., Maywood, E.S., Chesham, J.E., Loudon, A.S., and Hastings, M.H. (2013). A Gq-Ca2+ axis controls circuit-level encoding of circadian time in the suprachiasmatic nucleus. Neuron *78*, 714–728.

Brown, T.M., Hughes, A.T., and Piggins, H.D. (2005). Gastrin-releasing peptide promotes suprachiasmatic nuclei cellular rhythmicity in the absence of vasoactive intestinal polypeptide-VPAC2 receptor signaling. J. Neurosci. *25*, 11155–11164.

Brown, T.M., Colwell, C.S., Waschek, J.A., and Piggins, H.D. (2007). Disrupted neuronal activity rhythms in the suprachiasmatic nuclei of vasoactive intestinal polypeptide-deficient mice. J. Neurophysiol. *97*, 2553–2558.

DeBruyne, J.P., Noton, E., Lambert, C.M., Maywood, E.S., Weaver, D.R., and Reppert, S.M. (2006). A clock shock: mouse CLOCK is not required for circadian oscillator function. Neuron *50*, 465–477.

Feng, D., Liu, T., Sun, Z., Bugge, A., Mullican, S.E., Alenghat, T., Liu, X.S., and Lazar, M.A. (2011). A circadian rhythm orchestrated by histone deacetylase 3 controls hepatic lipid metabolism. Science *331*, 1315–1319.

Godinho, S.I., Maywood, E.S., Shaw, L., Tucci, V., Barnard, A.R., Busino, L., Pagano, M., Kendall, R., Quwailid, M.M., Romero, M.R., et al. (2007). The after-hours mutant reveals a role for Fbxl3 in determining mammalian circadian period. Science *316*, 897–900.

Gold, D.A., Gent, P.M., and Hamilton, B.A. (2007). ROR alpha in genetic control of cerebellum development: 50 staggering years. Brain Res. *1140*, 19–25.

Hatori, M., Gill, S., Mure, L.S., Goulding, M., O'Leary, D.D., and Panda, S. (2014). Lhx1 maintains synchrony among circadian oscillator neurons of the SCN. eLife *3*, e03357.

Ishii, Y., Kawaguchi, M., Takagawa, K., Oya, T., Nogami, S., Tamura, A., Miura, Y., Ido, A., Sakata, N., Hashimoto-Tamaoki, T., et al. (2003). ATBF1-A protein, but not ATBF1-B, is preferentially expressed in developing rat brain. J. Comp. Neurol. *465*, 57–71.

Koike, N., Yoo, S.H., Huang, H.C., Kumar, V., Lee, C., Kim, T.K., and Takahashi, J.S. (2012). Transcriptional architecture and chromatin landscape of the core circadian clock in mammals. Science *338*, 349–354.

Konopka, R.J., and Benzer, S. (1971). Clock mutants of Drosophila melanogaster. Proc. Natl. Acad. Sci. USA *68*, 2112–2116.

Lee, C., Etchegaray, J.P., Cagampang, F.R., Loudon, A.S., and Reppert, S.M. (2001). Posttranslational mechanisms regulate the mammalian circadian clock. Cell *107*, 855–867.

Lee, I.T., Chang, A.S., Manandhar, M., Shan, Y., Fan, J., Izumo, M., Ikeda, Y., Motoike, T., Dixon, S., Seinfeld, J.E., et al. (2015). Neuromedin s-producing neurons act as essential pacemakers in the suprachiasmatic nucleus to couple clock neurons and dictate circadian rhythms. Neuron *85*, 1086–1102.

Lein, E.S., Hawrylycz, M.J., Ao, N., Ayres, M., Bensinger, A., Bernard, A., Boe, A.F., Boguski, M.S., Brockway, K.S., Byrnes, E.J., et al. (2007). Genome-wide atlas of gene expression in the adult mouse brain. Nature *445*, 168–176.

Li, J.D., Hu, W.P., Boehmer, L., Cheng, M.Y., Lee, A.G., Jilek, A., Siegel, J.M., and Zhou, Q.Y. (2006). Attenuated circadian rhythms in mice lacking the prokineticin 2 gene. J. Neurosci. *26*, 11615–11623.

Liu, A.C., Welsh, D.K., Ko, C.H., Tran, H.G., Zhang, E.E., Priest, A.A., Buhr, E.D., Singer, O., Meeker, K., Verma, I.M., et al. (2007). Intercellular coupling confers robustness against mutations in the SCN circadian clock network. Cell *129*, 605–616.

Maywood, E.S., Reddy, A.B., Wong, G.K., O'Neill, J.S., O'Brien, J.A., McMahon, D.G., Harmar, A.J., Okamura, H., and Hastings, M.H. (2006). Synchronization and maintenance of timekeeping in suprachiasmatic circadian clock cells by neuropeptidergic signaling. Curr. Biol. *16*, 599–605.

Maywood, E.S., Chesham, J.E., O'Brien, J.A., and Hastings, M.H. (2011). A diversity of paracrine signals sustains molecular circadian cycling in suprachiasmatic nucleus circuits. Proc. Natl. Acad. Sci. USA *108*, 14306–14311.

Maywood, E.S., Drynan, L., Chesham, J.E., Edwards, M.D., Dardente, H., Fustin, J.M., Hazlerigg, D.G., O'Neill, J.S., Codner, G.F., Smyllie, N.J., et al. (2013). Analysis of core circadian feedback loop in suprachiasmatic nucleus of mCry1-luc transgenic reporter mouse. Proc. Natl. Acad. Sci. USA *110*, 9547–9552.

Meng, Q.J., Maywood, E.S., Bechtold, D.A., Lu, W.Q., Li, J., Gibbs, J.E., Dupré, S.M., Chesham, J.E., Rajamohan, F., Knafels, J., et al. (2010). Entrainment of disrupted circadian behavior through inhibition of casein kinase 1 (CK1) enzymes. Proc. Natl. Acad. Sci. USA *107*, 15240–15245.

Mori, Y., Kataoka, H., Miura, Y., Kawaguchi, M., Kubota, E., Ogasawara, N., Oshima, T., Tanida, S., Sasaki, M., Ohara, H., et al. (2007). Subcellular localization of ATBF1 regulates MUC5AC transcription in gastric cancer. Int. J. Cancer *121*, 241–247.

Prosser, H.M., Bradley, A., Chesham, J.E., Ebling, F.J., Hastings, M.H., and Maywood, E.S. (2007). Prokineticin receptor 2 (Prokr2) is essential for the regulation of circadian behavior by the suprachiasmatic nuclei. Proc. Natl. Acad. Sci. USA *104*, 648–653.

Qi, Y., Ranish, J.A., Zhu, X., Krones, A., Zhang, J., Aebersold, R., Rose, D.W., Rosenfeld, M.G., and Carrière, C. (2008). Atbf1 is required for the Pit1 gene early activation. Proc. Natl. Acad. Sci. USA *105*, 2481–2486.

Ralph, M.R., Foster, R.G., Davis, F.C., and Menaker, M. (1990). Transplanted suprachiasmatic nucleus determines circadian period. Science *247*, 975–978.

Sakata, N., Kaneko, S., Ikeno, S., Miura, Y., Nakabayashi, H., Dong, X.Y., Dong, J.T., Tamaoki, T., Nakano, N., and Itoh, S. (2014). TGF-β signaling cooperates with AT motif-binding factor-1 for repression of the α-fetoprotein promoter. J. Signal Transduct. *2014*, 970346.

Shinohara, K., Tominaga, K., Isobe, Y., and Inouye, S.T. (1993). Photic regulation of peptides located in the ventrolateral subdivision of the suprachiasmatic nucleus of the rat: daily variations of vasoactive intestinal polypeptide, gastrin-releasing peptide, and neuropeptide Y. J. Neurosci. *13*, 793–800.

Sun, X., Fu, X., Li, J., Xing, C., Martin, D.W., Zhang, H.H., Chen, Z., and Dong, J.T. (2012). Heterozygous deletion of Atbf1 by the Cre-loxP system in mice causes preweaning mortality. Genesis *50*, 819–827.

Ukai, H., and Ueda, H.R. (2010). Systems biology of mammalian circadian clocks. Annu. Rev. Physiol. *72*, 579–603.

VanDunk, C., Hunter, L.A., and Gray, P.A. (2011). Development, maturation, and necessity of transcription factors in the mouse suprachiasmatic nucleus. J. Neurosci. *31*, 6457–6467.

Vitaterna, M.H., King, D.P., Chang, A.M., Kornhauser, J.M., Lowrey, P.L., McDonald, J.D., Dove, W.F., Pinto, L.H., Turek, F.W., and Takahashi, J.S. (1994). Mutagenesis and mapping of a mouse gene, Clock, essential for circadian behavior. Science *264*, 719–725.

Yamaguchi, Y., Suzuki, T., Mizoro, Y., Kori, H., Okada, K., Chen, Y., Fustin, J.M., Yamazaki, F., Mizuguchi, N., Zhang, J., et al. (2013). Mice genetically deficient in vasopressin V1a and V1b receptors are resistant to jet lag. Science *342*, 85–90.

Yasuda, H., Mizuno, A., Tamaoki, T., and Morinaga, T. (1994). ATBF1, a multiple-homeodomain zinc finger protein, selectively down-regulates AT-rich elements of the human alpha-fetoprotein gene. Mol. Cell. Biol. *14*, 1395–1401.

Zambelli, F., Pesole, G., and Pavesi, G. (2009). Pscan: finding over-represented transcription factor binding site motifs in sequences from co-regulated or co-expressed genes. Nucleic Acids Res. *37*, W247–W252.

Zhang, E.E., and Kay, S.A. (2010). Clocks not winding down: unravelling circadian networks. Nat. Rev. Mol. Cell Biol. *11*, 764–776.

Zhang, E.E., Liu, A.C., Hirota, T., Miraglia, L.J., Welch, G., Pongsawakul, P.Y., Liu, X., Atwood, A., Huss, J.W., 3rd, Janes, J., et al. (2009). A genome-wide RNAi screen for modifiers of the circadian clock in human cells. Cell *139*, 199–210.

Supplemental Figures

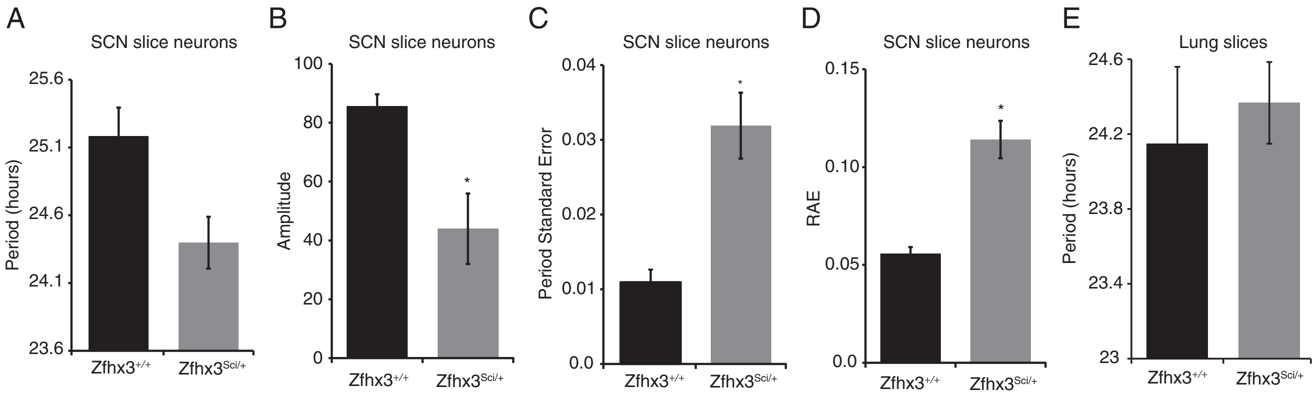

Figure S1. Disruption of PER2::LUC Rhythm in Individual Neurons In Ex Vivo SCN Slices, but Not in Ex Vivo Lung Slices, Related to Figure 1
(A–D) The (A) period and (B) amplitude of fusion protein expression was decreased in individual neurons from *Zfhx3*$^{Sci/+}$ ex vivo SCN slices compared to similar wild-type neurons. Furthermore, the (C) period distribution was broader, and (D) RAE increased in the *Zfhx3*$^{Sci/+}$ neurons ($p < 0.05$, t test). There were no differences in period in organotypic lung slices (Figure S1E), suggesting these differences may be specific to the central oscillator.

Cell

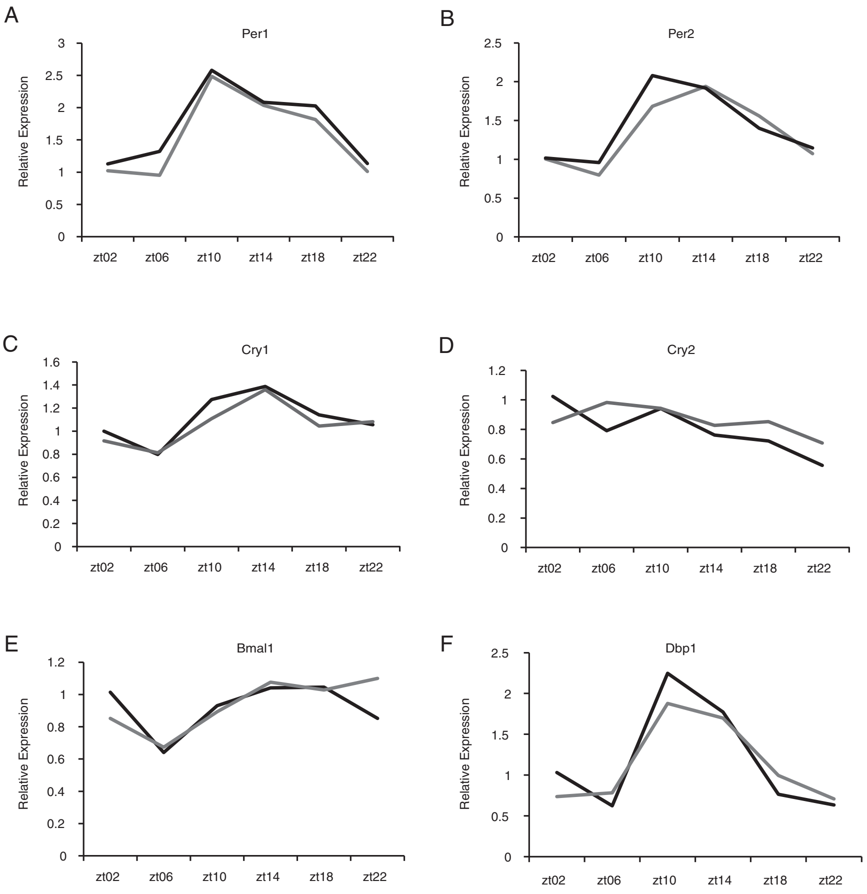

Figure S2. Circadian Gene Expression in *Zfhx3*$^{Sci/+}$ and *Zfhx3*$^{+/+}$ SCN across the Light:Dark Cycle, Related to Figure 1

(A–F) mRNA expression for (A) *Per1*, (B) *Per2*, (C) *Cry1*, (D) *Cry2*, (E) *Bmal1*, and (F) *Dbp1* showed no significant differences in the SCN of *Zfhx3*$^{Sci/+}$ (gray lines) compared to *Zfhx3*$^{+/+}$ (black lines) at multiple time points throughout the day (n = 4, p > 0.2, ANOVA).

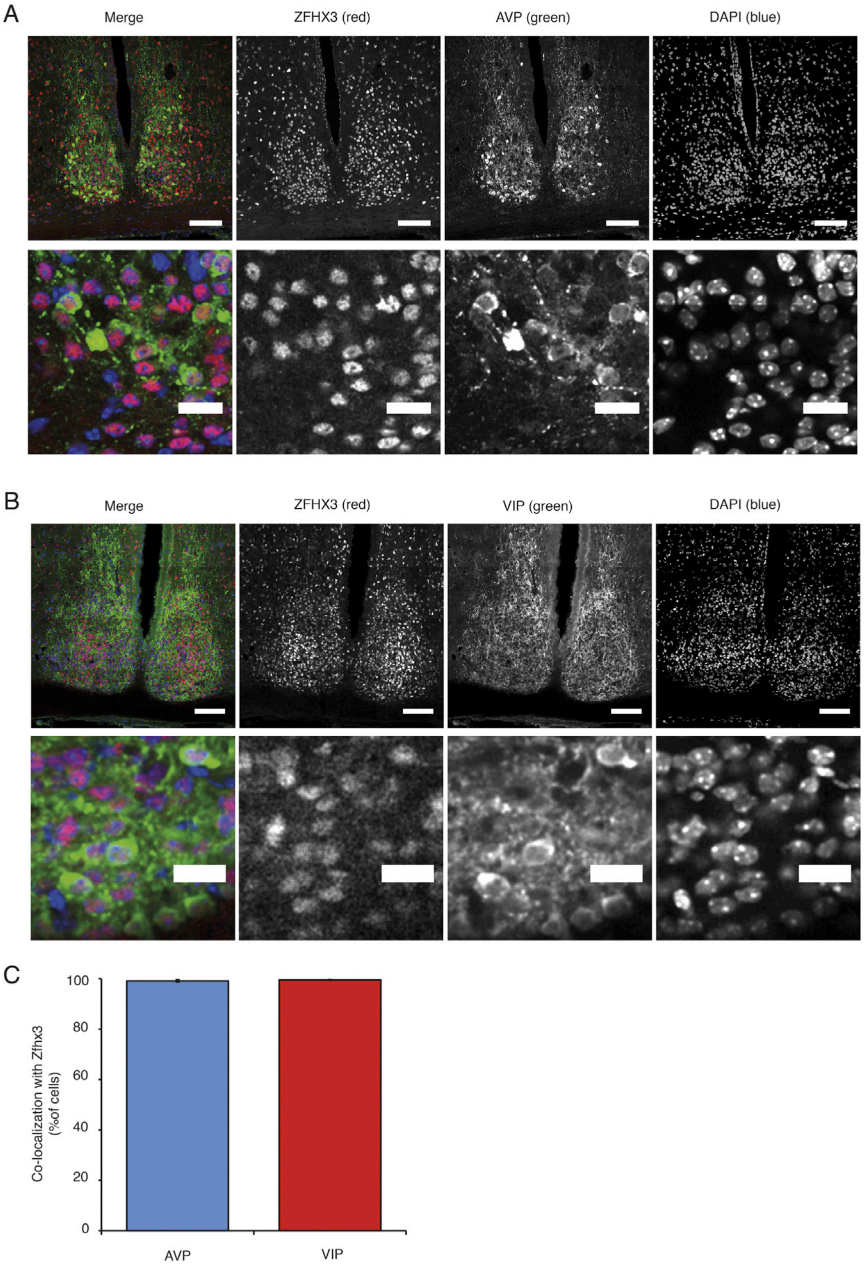

Figure S3. AVP and VIP Immunofluorescence Co-localizes with ZFHX3 Immunofluorescence, Related to Figure 4

(A and B) Confocal micrographs showing co-immunofluorescence of ZFHX3 and (A) AVP or (B) VIP within the SCN (scale bar, 100 μm) and at high magnification (scale bar, 20 μm) at ZT4.

(C) Both AVP (blue bar) and VIP (red bar) neurons almost completely co-localized with ZFHX3 (n = 3 brains), graph shows mean ± SEM for the percentage of co-localized cells.

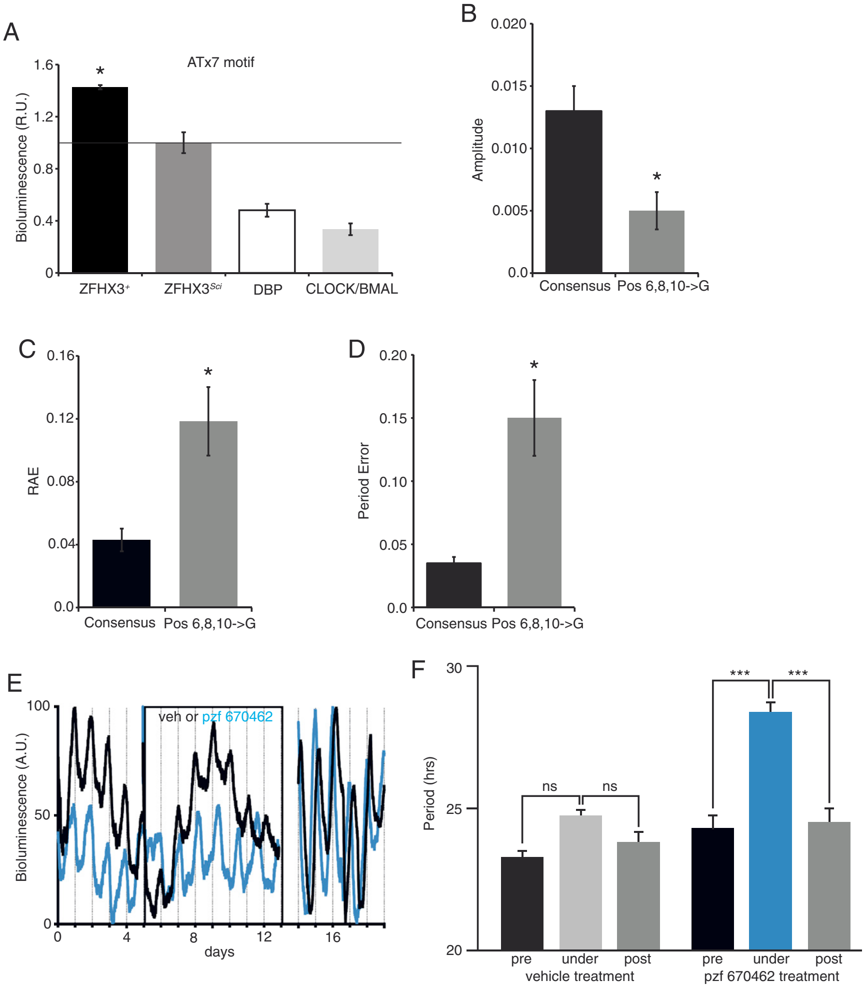

Figure S4. ZFHX3$^+$ Preferentially Activates a Novel, Circadian Motif in SCN, Related to Figure 5

(A) In vitro activation of the AT motif by recombinant ZFHX3, DBP, or combined CLOCK and BMAL using a luciferase reporter construct driven by the AT motif (×7) in HEK293 cells. *Zfhx3*$^+$ activation of the AT motif was more than 3 times that seen for either DBP or CLOCK/BMAL ($p < 0.05$, t test). Activation of AT sequences in SCN slices transduced by LVs showed cyclic activation in SCN slices from *Zfhx3*$^{+/+}$ animals (Figures 5B and 5C).

(B–D) Mutations of the three most conserved residues (positions 6, 8, 10) in the AT motifs strongly reduced AT activation rates (gray bars) when compared to the AT consensus (black bars). This effect was quantified by measuring both the (B) amplitude and robustness of these oscillations, (C) relative amplitude error, and (D) period error ($p < 0.05$, t test).

(E) SCN slices expressing the AT-luciferase reporters were treated with pzf 670462 1 μM (blue lines) or vehicle (black lines), respectively.

(F) Period of AT-mediated oscillations was significantly lengthened by pzf 670462 ($p < 0.001$, 2-way ANOVA repeated-measures), whereas it was unaffected by vehicle treatment. This effect was reversible upon removal of the drug.

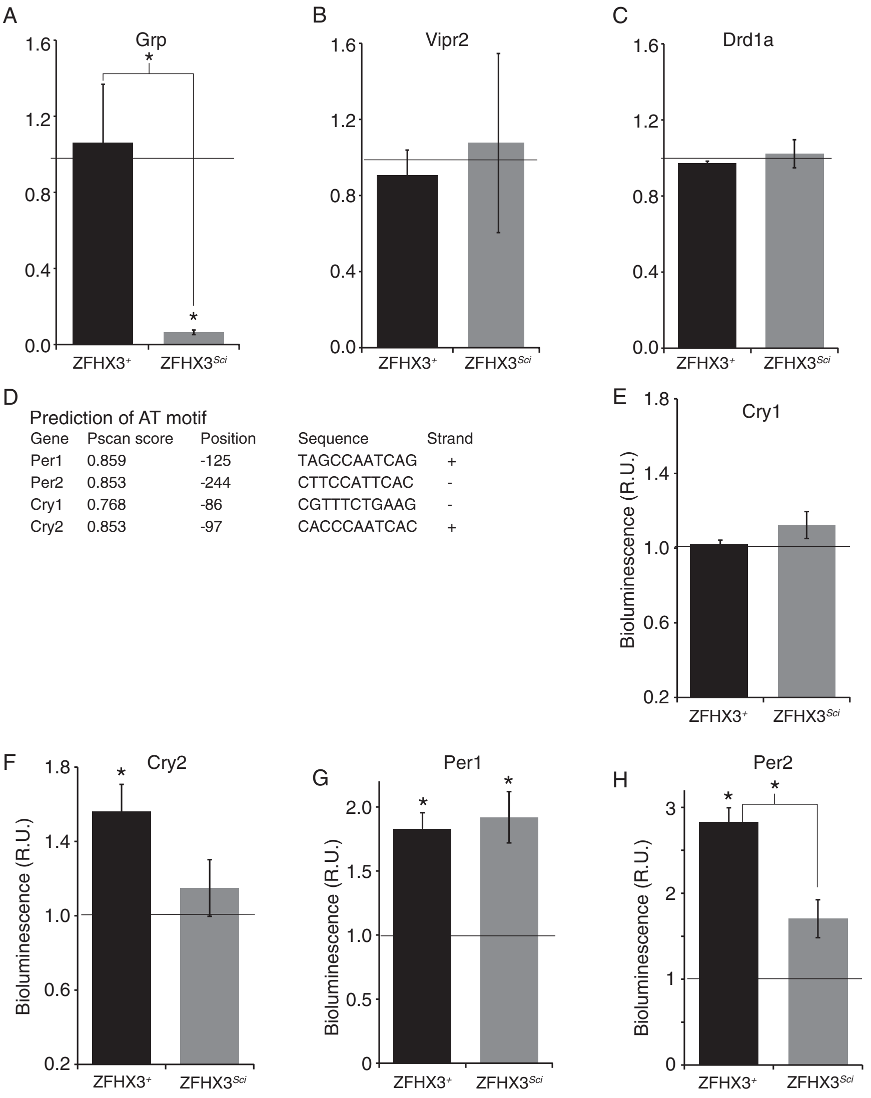

Figure S5. ZFHX3^{SCI} Interaction with the AT Motif in Circadian Gene Promoters, Related to Figure 6

(A–C) Overexpression of recombinant ZFHX3^{Sci} failed to activate the (A) *Grp*, (B) *Vipr2*, and (C) *Drd1a* promoter containing vectors ($p > 0.05$, t test, in HEK293 cells).

(D) *Cry2*, *Per1*, and *Per2* have a predicted AT motif in their promoters, whereas *Cry1* does not (Pscan score and sequences shown; position is relative to start of first exon).

(E) Co-transfection of ZFHX3^{+} failed to activate the *Cry1* promoter, while it did activate the (F) *Cry2*, (G) *Per1*, and (H) *Per2* promoters ($p < 0.05$, t test, in HEK293 cells). We found a small decrease in ZFHX3^{Sci} activation compared to ZFHX3^{+} for the *Per2* promoter ($p < 0.05$, t test, in HEK293 cells).

**AFP**		**MRF4**		**Pit1**		**MUC5AC**	
**Human**	tgattaataattaca	**Human**	ctaattaaatg				
**Rhesus**	tgattaataattaca	**Rhesus**	ctaattaaatg	**Human**	gattatttaattataa		
**Mouse**	tgg------gttcca	**Mouse**	ctaattaaatg	**Rhesus**	gattatttaattataa	**Human**	tttattcattaac
**Dog**	---ttaataattaca	**Dog**	ctaattaaatg	**Mouse**	gattatttaattagaa	**Rhesus**	ttcatccattcac
**Elephant**	ag-ttaataat----	**Platypus**	ctaattaaatg	**Dog**	ggttatttaattataa	**Mouse**	tttgttcattcat

Figure S6. Conservation of ZFHX3 Binding Sites across Species, Related to Experimental Procedures

A multiple sequence alignment for ZFHX3 binding sites for *Afp*, *Mrf4*, *Pit1*, and *Muc5ac* reveals conservation of the motif across both primates and placental mammals.

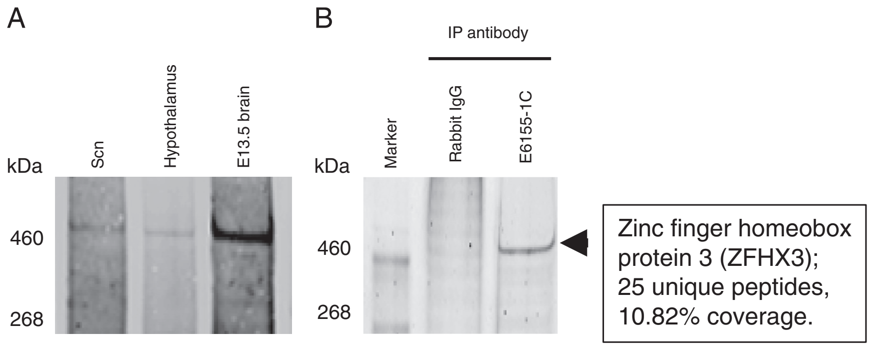

Figure S7. Validation of the ZFHX3 Antibody, Related to Experimental Procedures

Expression and characterization of ZFHX3 in mouse tissues.

(A) Western blot analysis showing ZFHX3 immunoreactive product in mouse SCN, hypothalamus, and embryonic brain lysates.

(B) Immunoprecipitation of ZFHX3 using anti-ZFHX3 antibody and validation by mass spectrometry.

Cell

Supplemental Information

The Regulatory Factor ZFHX3 Modifies Circadian Function in SCN via an AT Motif-Driven Axis

Michael J. Parsons, Marco Brancaccio, Siddharth Sethi, Elizabeth S. Maywood, Rahul Satija, Jessica K. Edwards, Aarti Jagannath, Yvonne Couch, Mattéa J Finelli, Nicola J. Smyllie, Christopher Esapa, Rachel Butler, Alun R. Barnard, Johanna E. Chesham, Shoko Saito, Greg Joynson, Sara Wells, Russell G. Foster, Peter L Oliver, Michelle M. Simon, Ann-Marie Mallon, Michael H. Hastings, and Patrick M. Nolan

Supplemental Experimental Procedures

Mice

When not being tested, mice were housed in individually ventilated cages under 12/12 h light/dark (LD) conditions with food and water available *ad libitum*. Inbred strains and mutant colonies were bred and maintained at MRC Harwell. The Zfhx3$^{Flox/+}$ mice were imported from Dr. Dong's laboratory (Emory University)and are described in detail elsewhere (Sun et al., 2012).

ENU Screen and Genetic Localization of the *Sci* Circadian Mutant

Wheel-running activity was used to identify circadian phenodeviants following ENU mutagenesis (Bacon et al., 2004). The original *Sci* founder, on a C3H/HeH x BALB/cAnNCrl background, displayed reduced amplitude, a short circadian period length under constant darkness and a reduced or absent phase shift in response to a light pulse in constant darkness. The mutant phenotype was tested for inheritance, crossing to C3H/HeH and the behavioral phenotype was completely penetrant on this background. Low resolution mapping identified a novel mutant locus to be associated with circadian rhythms, from 101.86 Mb to 110.46 Mb on distal chromosome 8 (see Bacon et al., 2004), followed by positional sequencing of *in silico* prioritized candidate genes to reveal the causal mutation. All genes within this locus underwent complete Sanger sequencing of their coding regions. Besides the Sci mutation, no other mutations were detected. The *Sci* animals were bred onto both C57BL/6J and C3H/HeH backgrounds and backcrossed for 10 generations until congenic, ensuring that other functional ENU induced mutations were removed. Breeding difficulties however were encountered on both backgrounds. To circumvent these problems, the *Sci* animals were maintained on a mixed C3H/HeH x C57BL6/J F1 background. The mutation for *Sci* was genotyped using a real time PCR (RT-PCR) Taqman assay. DNA extracted from ear biopsies was diluted 1:20 in H_2O for the reaction (forward primer: TCCACGCATTGCTTCAGATG; reverse primer: TGTGCCTTCTGCTTGTTCTCA; wildtype probe:

CTTTGAGCTCGTCATT; mutant probe: TTTGAGCTCTTCATTCA). Circadian wheel running analysis was performed as outlined in (Banks and Nolan, 2011). Mice were individually housed in cages within light-tight chambers, kept under environmentally controlled conditions. Cages contained running wheels in which the revolutions were monitored and plotted as activity on double-plotted actograms. Initially, cages were exposed to a week-long entrainment period of a 12:12 light-dark cycle, followed by two weeks of constant darkness to assess the free-running ability. A light intensity of 100 lux using a fluorescent light source was used for all screens. Food and water were provided *ad libitum*. Littermate controls were run alongside heterozygous mutants.

SCN Collection and RNA Extraction

SCN punches were collected as in (Jagannath et al., 2013). We verified the quality of the dissection by checking for enrichment of *Six6,* a SCN specific marker, compared to cortical punches. Total RNA was extracted using the RNAeasy column method (QIAGEN, Hilden, Germany). Quality and quantity of RNA were measured using an Agilent Bioanalyzer and a Nanodrop1000 (Thermo Fisher Scientific, Waltham, MA USA), respectively.

qPCR

cDNA was synthesized from 100 ηg of SCN RNA using the Invitrogen SuperScript III First Strand cDNA Synthesis SuperMix (Life Technologies, Grand Island, NY), and qPCR was conducted with Sybr green I using an SDS7700 thermal cycler (Applied Biosystems, Foster City, CA). Relative quantification of transcript levels was carried out as described previously (Jagannath et al., 2013). The geometric mean of three housekeeping genes was used for normalization (*Gapdh*, *B2m, Psmb2)*. Primer sequences are provided in **Table S5**.

Luciferase Reporter Gene Assays

We used Jetprime (Polyplus Transfection, Illkirch, France) to co-transfect 100 ng of the pGL3 vectors, 400 ng of the Zfhx3 pcDNA™3.1/V5-His vector and 5 ng of the pRL-TK Vector (renilla) into HEK293 cells. We used the Dual-Luciferase Reporter Assay (Promega, Madison, WI) to quantify the luciferase activity, normalizing to renilla activity (Varioskan, Thermo Fisher Scientific Inc., Waltham, MA, USA). All experiments were conducted in triplicate.

RNA Sequencing

We sent 500 ηg of SCN RNA samples to the Oxford Genomics Centre (Wellcome Trust Centre for Human Genetics, University of Oxford). The samples underwent poly-A selection, after which two multiplexed DSN Library Preparations (6 samples/multiplex) were generated. Each multiplex was run on one 50bp PE lane of a HiSeq2000.

RNA Sequencing Analysis

Sequence data were aligned to the genome using TopHat (Trapnell et al., 2009) resulting in counts for genomic regions where reads accumulate. Read counts were calculated using HTSeq (Anders et al., 2014) and statistical analysis of differentially expressed genes was determined using three softwares; EdgeR (Robinson et al, 2010), DESeq (Anders and Huber, 2010) and Cufflinks (Trapnell et al., 2010), default parameters were used in all cases. A filtering strategy was employed on all three datasets to reduce false positives. Genes with $q>0.05$ and log2 fold change < 1 were removed from further analysis. Remaining genes common to at least two datasets were used for validation and further analysis. The RNA sequence data has been deposited in the European Nucleotide Archive (accession ID: PRJEB9284).

Network Analysis for RNA Sequencing Results

Protein-protein interactions for significant differentially expressed genes were obtained from STRING (Franceschini et al., 2013), visualization was performed with Cytoscape (Shannon et al., 2003). Clusters of densely connected nodes were found using the default parameters in the Molecular Complex Detection (MCODE) (Bader and Hogue, 2003) plugin for Cytoscape. Functional enrichment of Gene Ontology terms was performed using g:Profiler. Significant terms were determined using the default settings and corrected for multiple testing using g:Profiler's native method g:SCS (Reimand et al., 2007). The threshold for significance was $p<0.05$.

Determination of Zfhx3 Specific AT Motif

To construct a motif-binding model for ATBF1, we searched the literature for previously identified binding sites. We found four cases (*Afp*, *Mrf4*, *Muc5ac*, *Pit1*) (Berry et al., 2001; Mori et al., 2007; Qi et al., 2008; Yasuda et al., 1994) where ATBF1 was shown to directly regulate a downstream gene through a characterized binding motif, as primarily demonstrated by luciferase assays. Examining a mammalian multiple sequence alignment on the UCSC genome browser (Blanchette et al., 2004), we observed that ATBF1 binding sites were conserved across both primates and placental mammals (**Figure S6**). We therefore constructed a preliminary binding motif for ATBF1 on the sequences in **Figure 3A** using a mixture model by expectation maximization (MEME) (Bailey and Elkan, 1994).

***Ex vivo* SCN Slice Experiments**

For Zfhx3$^{flox/+}$ ex vivo experiments, SCN slices from Zfhx3$^{+/+}$ and Zfhx3$^{flox/+}$ mice were transduced by LV encoding for AT-luc. One week after LV transduction, PMT recording of AT-luc was started and performed for >7days. Subsequently, Zfhx3$^{flox/+}$ and Zfhx3$^{+/+}$ matched samples were transduced with AAV expressing Syn-mCherry:Cre (Penn Vector Core) to delete the Zfhx3 floxed allele, and immediately

put back for PMT luciferase recording. Period was assessed before AAV treatment and after >7 days to allow for effective Cre recombination. Following recordings, Zfhx3 expression levels in slices were determined by qPCR.

Vector Construction

Mouse *Zfhx3* cDNA was cloned into the pcDNA™3.1/V5-His vector (amino acid positions 1787 to 3723 from transcript ENSMUSG00000038872). The Zfhx3^{Sci} mutation was introduced into this construct using site-directed mutagenesis using the QuikChange® II XL Site-Directed Mutagenesis Kit (Agilent Technologies, Santa Clara, CA, USA). The CLOCK and BMAL expression vectors were generously provide by Dr. Filippo Tamanini. The DBP expression vector was generously provide by Dr. Jürgen Ripperger. To generate the AT motif driven luciferase reporter vectors, we generated forward and reverse oligonucleotides containing x7 consensus motif sequence ATTTAATTAT with an AACT linker sequence and *XhoI* sites on either side, hybridized these oligos and cloned them into both the pGL3-Enhancer and lentivirus Luciferase reporter vectors. We cloned approximately 1 kb of the putative promoters for *Avp*, *Drd1a*, *Grp*, *Prokr2*, *Vip and Vipr2* upstream of the pGL3-Enhancer Luciferase Reporter vector (see **Table S6**). The pGL3-Enhancer Luciferase Reporter vector containing the Cry1 and Cry2 promoters were generous provided by Dr. Saito. The pGL3-Enhancer Luciferase Reporter vector containing the Per1 and Per2 promoters were provided by Dr. Hastings. To test the specificity of the AT motif, we mutated its 3 most conserved residues (residues 6, 8 and 10) in the pGL3-Enhancer Luciferase Reporter vector containing the *Avp* and *Vip* promoters.

Antisera

Two peptides corresponding to amino acid residues 21-49 (1A) and 2114-2154 (1C) of mouse ZFHX3 were synthesized and used to immunize rabbits (Epitomics). Antisera were affinity purified on Sulfolink coupling gel (Pierce) before antibody characterization. Western blot analysis using the affinity purified antibodies identified immunoreactive products of ~460kDa in mouse SCN, hypothalamus and E13.5 embryonic brain lysates (**Figure S7A**). In order to verify the identity of this immunoreactive product, 4.5mg of E13.5 embryonic brain lysate was immunoprecipitated using anti-ZFHX3 antibody overnight at 4°C, followed by incubation with protein G sepharose beads for 2h at 4ºC. Bound proteins were eluted from the beads by adding 40µl LDS sample buffer (Invitrogen) and heating at 95ºC for 5min. Eluted proteins were separated on 3-8% Tris-Acetate gel and stained with AquaStain (**Figure S7B**). Analysis by mass spectrometry identified ZFHX3 as the major protein in the ~460 kDa gel band thereby confirming the specificity of the anti-ZFHX3 antibody.

Immunofluorescence

Confocal microscopy was used to image immunoflourescent staining from free-floating brain sections (40um) (using rabbit anti-ZFHX3 (1:2000 see supplemental methods), rabbit anti-AVP (1:1000 Bachem, USA), guinea-pig anti-VIP (1:1000 Bachem, USA) and rabbit anti-GRP (1:1000 Immunostar, USA) primary antibodies. Alexa-Fluor secondary antibodies (Invitrogen, USA) were used at a dilution of 1:1000 (594 goat anti-rabbit or 488 goat anti- guinea-pig). Sections were slide-mounted using Vectashield mounting medium plus DAPI (Vector Laboratories, USA). To quantify the immunostaining, confocal images were analyzed using ImageJ software by outlining the SCN from the DAPI stained image and using this template to measure the relative intensity of the immunostaining for each neuropeptide. Where more than one section was analyzed from an animal, the mean of the consecutive measures were used for that individual.

In Vivo RNAi

In vivo RNAi experiments were conducted as previously reported (Jagannath et al., 2013). We used the *in vivo* formulation of siRNAs for *Zfhx3* (assay ID: ss62675; Life Technologies, United Kingdom). Briefly, the siRNA was complexed with Invivofectamine 2.0 (Invitrogen) prepared according to manufacturer's instructions and concentrated using Amicon Ultracel-100 to 5ug/ul. 1ul of this mix was delivered using microinjection into the third ventricle of anaesthetised mice, with sterotaxic equipment with coordinates as described previously (Butcher et al., 2002; Cheng et al., 2007). Mice were allowed to recover and returned to the light-tight chambers for further experiments. To measure silencing of *Zfhx3*, mice were injected with either siNT or siZfhx3 and 96 hours later were sacrificed. SCN was collected, RNA extracted and qPCR conducted on the relevant mRNAs. For studies on circadian period length, C57Bl/6 mice (8 weeks of age) were maintained on running wheels in light tight chambers on a 12:12 LD cycle (400 lux from white LED lamps) and injected with siRNA as above. 1 day after the injection, the mice were placed in DD and running wheel activity data were collected and analysed on Clocklab (Actimetrics, Wilmette, IL). Data on onset of activity for 10 days following treatment were used to calculate period length.

Chromatin Immunoprecipitation (ChIP)

Assays were performed using the ChIP-IT enzymatic kit according to the manufacturer's instructions (Active Motif). Briefly, SCN hypothalamic punches were pooled from four sex-matched mice at ZT3 and immediately fixed for 10 min at room temperature with gentle shaking in 1% formaldehyde. The cross-linking reaction was stopped by addition of glycine to a final concentration of 0.25 M. Cross-linked chromatin was sheared by enzymatic digestion following the manufacturer's recommendations. Samples were incubated with 3µg of antiserum against ZFHX3, together with magnetic beads, on a rotator at 4°C overnight. This was followed by crosslinking reversal and proteinase-K digestion as per the

manufacturer's protocol. The recovered DNA was purified using the QIAquick PCR purification kit (Qiagen) and subjected to quantitative PCR using Fast SYBR Green (Life Technologies). Primer pairs were designed upstream of the transcription start site and within the coding region of Avp and Vip with at least one pair covering a predicted AT motif, in addition to the promoter of the negative control gene, Gapdh. The binding of ZFHX3 to adjacent promoter regions (primers spanning approximately 160 bp) was calculated using the ΔΔCt method; non-immunoprecipitated input (diluted 1:10) and primers in the coding region of each gene were used to normalise each reaction. Three independent cohorts of four animals were used as biological replicates and each individual PCR reaction was carried out in triplicate. Primer sequences are available upon request.

Supplemental References

Anders, S., Huber., W. (2010). Differential expression analysis for sequence count data. Genome Biol. *11*,R106.

Anders, S., Pyl, P.T., Huber, W. (2014). A Python framework to work with high-throughput sequencing data. Bioinformatics. 638.

Bacon, Y., Ooi, A., Kerr, S., Shaw-Andrews, L., Winchester, L., Breeds, S., Tymoska-Lalanne, Z., Clay, J., Greenfield, A. G., Nolan, P. M. (2004). Screening for novel ENU-induced rhythm, entrainment and activity mutants. Genes, Brain and Behavior, *3*,196–205.

Bader, G.D., Hogue, C.W. (2003). An automated method for finding molecular complexes in large protein interaction networks. BMC Bioinformatics. *13*,4:2.

Bailey, T.L., Elkan, C. (1994). Fitting a mixture model by expectation maximization to discover motifs in biopolymers. Proceedings of the Second International Conference on Intelligent Systems for Molecular Biology, pp. 28-36, AAAI Press, Menlo Park, California.

Banks, G.T., Nolan, P.M. (2011). Assessment of circadian and light-entrainable parameters in mice using wheel-running activity. Curr. Prot. Mouse Biol. *1*,369-381.

Berry, F.B., Miura, Y., Mihara, K., Kaspar, P., Sakata, N., Hashimoto-Tamaoki, T., Tamaoki, T. (2001). Positive and negative regulation of myogenic differentiation of C2C12 cells by isoforms of the multiple homeodomain zinc finger transcription factor ATBF1. J Biol Chem. *276*,25057-65.

Blanchette, M., Kent, W.J., Riemer, C., Elnitski, L., Smit, A.F., Roskin, K.M., Baertsch, R., Rosenbloom, K., Clawson, H., Green, E.D., et al. (2004). Aligning multiple genomic sequences with the threaded blockset aligner. Genome Res. *14* ,708-15.

Franceschini, A., Szklarczyk, D., Frankild, S., Kuhn, M., Simonovic, M., Roth, A., Lin, J., Minguez, P., Bork, P., von Mering, C., Jensen, L.J. (2012). STRING v9.1: protein-protein interaction networks, with increased coverage and integration. Nucleic Acids Res. *41*,D808-15.

Jagannath, A., Butler, R., Godinho, S.I., Couch, Y., Brown, L.A., Vasudevan, S.R., Flanagan, K.C., Anthony, D., Churchill, G.C., Wood, M.J., et al. (2013). The CRTC1-SIK1 pathway regulates entrainment of the circadian clock. Cell. *154*,1100-11.

Mori, Y., Kataoka, H., Miura, Y., Kawaguchi, M., Kubota, E., Ogasawara, N., Oshima, T., Tanida, S., Sasaki, M., Ohara, H., et al. (2007). Subcellular localization of ATBF1 regulates MUC5AC transcription in gastric cancer. Int J Cancer. *121*,241-7.

Qi, Y., Ranish, J.A., Zhu, X., Krones, A., Zhang, J., Aebersold, R., Rose, D.W., Rosenfeld, M.G., Carrière, C. (2008). Atbf1 is required for the Pit1 gene early activation. Proc Natl Acad Sci U S A. *105*,2481-6.

Reimand J., Kull M., Peterson. H., Hansen, J., Vilo, J. (2007). G:Profiler - a web-based toolset for functional profiling of gene lists from large-scale experiments. Nucleic Acids Res. 35,W193-W200.

Robinson, M.D., McCarthy, D.J. Smyth, G.K. (2010). EdgeR: a Bioconductor package for differential expression analysis of digital gene expression data. Bioinformatics. *26*,139-40.

Shannon, P., Markiel, A., Ozier, O., Baliga, N.S., Wang, J.T., Ramage, D., Amin, N., Schwikowski, B., Ideker, T. (2003). Cytoscape: a software environment for integrated models of biomolecular interaction networks. Genome Res. *13*,2498-504.

Sun X, Fu X, Li J, Xing C, Martin DW, Zhang HH, Chen Z, Dong JT. (2012) Heterozygous deletion of Atbf1 by the Cre-loxP system in mice causes preweaning mortality. Genesis. *50*, 819-27.

Trapnell, C., Pachter, L., Salzberg, S.L. (2009). TopHat: discovering splice junctions with RNA-Seq. Bioinformatics *25*,1105-11.

Trapnell, C., Williams, B.A., Pertea, G., Mortazavi, A., Kwan, G., van Baren, M.J., Salzberg, S.L., Wold, B.J., Pachter, L. (2010). Transcript assembly and quantification by RNA-Seq reveals unannotated transcripts and isoform switching during cell differentiation. Nat Biotechnol *28*,511-5.

Yasuda, H., Mizuno, A., Tamaoki, T., Morinaga, T. (1994). ATBF1, a multiple-homeodomain zinc finger protein, selectively down-regulates AT-rich elements of the human alpha-fetoprotein gene. Mol Cell Biol. *14*,1395-401.

Table S2. Gene Ontology Enrichment of Differentially Expressed Genes, Related to Figure 2

Profiler was used to find the enriched molecular function and biological process terms for the differential gene list predicted from RNA-sequencing data at time interval ZT3 and ZT15 (q<0.05). BP is biological process.

GO ID	p-value	X	GO Category	Term Name
**GO:0044057**	5.28E-03	12	BP	regulation of system process
**GO:0006139**	6.79E-03	54	BP	nucleobase-containing compound metabolic process
**GO:0031326**	6.87E-03	41	BP	regulation of cellular biosynthetic process
**GO:0021953**	2.32E-02	8	BP	central nervous system neuron differentiation
**GO:0051239**	5.78E-03	31	BP	regulation of multicellular organismal process
**GO:1901360**	8.04E-04	59	BP	organic cyclic compound metabolic process

**GO:1901576**	2.59E-03	53	BP	organic substance biosynthetic process
**GO:0009889**	1.18E-02	41	BP	regulation of biosynthetic process
**GO:0051171**	3.83E-02	41	BP	regulation of nitrogen compound metabolic process
**GO:0034641**	4.92E-04	59	BP	cellular nitrogen compound metabolic process
**GO:0046483**	2.79E-03	56	BP	heterocycle metabolic process
**GO:0006725**	3.52E-03	56	BP	cellular aromatic compound metabolic process
**GO:0044249**	1.21E-03	53	BP	cellular biosynthetic process
**GO:0042127**	1.07E-02	22	BP	regulation of cell proliferation
**GO:0031323**	5.38E-04	54	BP	regulation of cellular metabolic process
**GO:0044708**	8.36E-03	12	BP	single-organism behavior
**GO:0030804**	9.24E-03	6	BP	positive regulation of cyclic nucleotide biosynthetic process
**GO:1900373**	1.17E-02	6	BP	positive regulation of purine nucleotide biosynthetic process
**GO:0030182**	8.43E-03	20	BP	neuron differentiation

Table S3. Gene Ontology Enrichment of Differentially Expressed Genes in Module 1, Related to Figure 3

Profiler was used to find the enriched molecular function - and biological process terms - for the genes in module 1. BP is biological process, MF is molecular function and CC is cellular component.

GO ID	p-value	X	GO Category	Term Name
**GO:0007186**	6.49E-12	17	BP	G-protein coupled receptor signaling pathway
**GO:0007218**	4.17E-09	7	BP	neuropeptide signaling pathway
**GO:0005179**	1.32E-08	7	MF	hormone activity
**GO:0007154**	1.82E-08	20	BP	cell communication
**GO:0001664**	2.24E-08	8	MF	G-protein coupled receptor binding
**GO:0007166**	8.09E-08	17	BP	cell surface receptor signaling pathway
**GO:0007165**	1.09E-07	19	BP	signal transduction
**GO:0005184**	2.76E-06	4	MF	neuropeptide hormone activity
**GO:0007204**	2.29E-05	6	BP	positive regulation of cytosolic calcium ion concentration
**GO:0051241**	2.45E-05	7	BP	negative regulation of multicellular organismal process
**GO:0008015**	2.50E-05	7	BP	blood circulation
**GO:0051480**	4.21E-05	6	BP	cytosolic calcium ion homeostasis
**GO:0044708**	4.36E-05	7	BP	single-organism behavior

**GO:0050880**	1.85E-04	5	BP	regulation of blood vessel size
**GO:0035150**	1.92E-04	5	BP	regulation of tube size
**GO:0006874**	2.25E-04	6	BP	cellular calcium ion homeostasis
**GO:0007200**	2.56E-04	4	BP	phospholipase C-activating G-protein coupled receptor signaling pathway
**GO:0055074**	2.86E-04	6	BP	calcium ion homeostasis
**GO:0072503**	3.09E-04	6	BP	cellular divalent inorganic cation homeostasis
**GO:0003018**	3.21E-04	5	BP	vascular process in circulatory system
**GO:0030819**	3.87E-04	4	BP	positive regulation of cAMP biosynthetic process
**GO:0072507**	4.18E-04	6	BP	divalent inorganic cation homeostasis
**GO:0042311**	4.69E-04	4	BP	vasodilation
**GO:0051239**	6.27E-04	11	BP	regulation of multicellular organismal process
**GO:0030804**	7.10E-04	4	BP	positive regulation of cyclic nucleotide biosynthetic process
**GO:0006875**	7.94E-04	6	BP	cellular metal ion homeostasis
**GO:0007626**	1.09E-03	5	BP	locomotory behavior
**GO:0051050**	1.15E-03	7	BP	positive regulation of transport
**GO:0030003**	1.27E-03	6	BP	cellular cation homeostasis
**GO:0030817**	1.39E-03	4	BP	regulation of cAMP biosynthetic process
**GO:0006873**	1.52E-03	6	BP	cellular ion homeostasis
**GO:0006171**	1.74E-03	4	BP	cAMP biosynthetic process
**GO:0023051**	1.90E-03	11	BP	regulation of signaling
**GO:0010646**	1.92E-03	11	BP	regulation of cell communication
**GO:0055065**	1.99E-03	6	BP	metal ion homeostasis
**GO:0071855**	2.14E-03	3	MF	neuropeptide receptor binding
**GO:0045762**	2.72E-03	3	BP	positive regulation of adenylate cyclase activity
**GO:0050878**	3.37E-03	5	BP	regulation of body fluid levels
**GO:0055080**	3.65E-03	6	BP	cation homeostasis
**GO:0007622**	3.77E-03	3	BP	rhythmic behavior
**GO:0007267**	7.61E-03	7	BP	cell-cell signaling
**GO:0045761**	9.83E-03	3	BP	regulation of adenylate cyclase activity
**GO:0008217**	1.48E-02	4	BP	regulation of blood pressure
**GO:0043270**	1.77E-02	4	BP	positive regulation of ion transport
**GO:0090066**	1.88E-02	5	BP	regulation of anatomical structure size
**GO:0007611**	4.31E-02	4	BP	learning or memory

Table S4. Gene Ontology Enrichment of Differentially Expressed Genes in Module 2, Related to Figure 3

Profiler was used to find the enriched molecular function - and biological process terms - for the genes in module 2. BP is biological process, MF is molecular function and CC is cellular component.

term ID	p-value	X	GO Category	Term Name
**GO:0006412**	4.06E-04	5	BP	translation
**GO:0032991**	7.98E-03	8	CC	macromolecular complex
**GO:0030529**	2.11E-03	5	CC	ribonucleoprotein complex
**GO:0005829**	3.27E-02	5	CC	cytosol
**GO:0044445**	3.58E-06	5	CC	cytosolic part
**GO:0005840**	7.51E-06	5	CC	ribosome
**GO:0044391**	1.23E-06	5	CC	ribosomal subunit
**GO:0015934**	4.12E-03	3	CC	large ribosomal subunit
**GO:0022626**	1.87E-07	5	CC	cytosolic ribosome
**GO:0022625**	1.29E-03	3	CC	cytosolic large ribosomal subunit
**GO:0005198**	2.59E-04	5	MF	structural molecule activity
**GO:0003735**	1.23E-04	4	MF	structural constituent of ribosome

Table S5. Quantitative PCR Primer Sequences, Related to Experimental Procedures

Gene	Forward Primer	Reverse Primer
Avp	GCTGCCAGGAGGAGAACTAC	AAAAACCGTCGTGGCACTC
B2m	GCTATCCAGAAAACCCCTCAA	CATGTCTCGATCCCAGTAGACGGT
Bmal1	CCGTGCTAAGGATGGCTGTT	TTGGCTTGTAGTTTGCTTCTG
Chat	GTGAGACCCTGCAGGAAAAG	GCCAGGCGGTTGTTTAGATA
Clock	TGTCTCAAGCTGCAAATTTACCA	TTTAGATGCTGCATGGCTCCTA
Cry1	GCTATGCTCCTGGAGAGAACGT	TGTCCCCGTGAGCATAGTGTAA
Cry2	TGACCTAGACAGAATCATCGAACTG	GGCTGATGAGGGCCTGAA
Dbp	GAGCCTTCTGCAGGGAAACA	GCCTTGCGCTCCTTTTCC
Drd1a	AAAGATCCAACCCGTTACCC	ACAGCAAGCCCTAGGGAACT
Gal	GTGACCCTGTCAGCCACTCT	GGTCTCCTTTCCTCCACCTC
Gapdh	ACGGGAAGCTCACTGGCATGGCCTT	CATGAGGTCCACCACCCTGTTGCTG
Grp	GCCTCTCAGTCTCCAGCCTA	GCAGTTCCTCCCTTTTCCTT
Nms	GCCAGCAGAAGGATGAAAAG	GGCCTGAAAAGGAAAAATGG
Per1	CCCCTGCCTCCCAGTGA	CTGAAAGTGCATCCTGATTGGA
Per2	AGCTACACCACCCCTTACAAGCT	GACACGGCAGAAAAAAGATTTCTC
Prok2	TGCGACAAGGACTCTCAGTG	TTCTTCTTTCCTGCCTTCCA
Prokr2	GGTCTCCCTGTACGTCTCCA	CAAAGCGATCAGGAAGGAAG
Psmb2	AAATGCGGAATGGATATGAATTG	GAAGACAGTCAGCCAGGTT
Reverba	CGTTCGCATCAATCGCAACC	GATGTGGAGTAGGTGAGGTC
Six6	GTGGGCAACTGGTTCAAAAA	AGATGTCGCACTCACTGTCG

Vip	CAGTTCCTGGCATTCCTGAT	GGTCACCTGCTCCTTCAAAC
Vipr2	GACGTTGGGGAAACTGTCAC	AGCCACACGCATCTATGAAA
Zfhx3	CCAATAGCCTGGAGAAGCTG	AGTTGCACAGGACACAGTGG

Table S6. Design of the Luciferase Reporter Gene Vectors Containing the Differentially Expressed Gene Promoters, Related to Experimental Procedures

Regions of the promoters for a subset of the genes that were differentially expressed in the SCN of *Zfhx3*$^{Sci/+}$ and *Zfhx3*$^{+/+}$ mice were cloned into pGL3-Enhancer Luciferase Reporter vector. These regions included the predicted AT motif. The region of the promoter included and the AT motif location is relative to the start of exon 1 for the given transcript.

**Promoter cloning**					**AT motif**		
**Gene**	**Transcript**	**Forward Primer**	**Reverse Primer**	**Region included**	**Pscan Score**	**Sequence**	**Location**
Avp	ENSMUST000 00046001	CTCGAGAGGC ATAGAAGCCA GCATGT	CTCGAGGCTG GGCTGCCTAT TTATGT	-1673 to -29	0.89	TATTCAACTAT	-325
Drd1a	ENSMUST000 00021932	CTCGAGCTCA GGTCACCTCC AGCTTC	CTCGAGGCTT CTGCGGTCAA CTCAC	-1818 to +8	0.80	TATTCTCTAAG	-320
Grp	ENSMUST000 00025395	CTCGAGGGAA CTCAGACCGG AGATTTT	CTCGAGGGAG GGAAACCCTC AGAGC	-781 to +152	0.84	CATCCATCTAC	-345
Prokr2	ENSMUST000 00049997	CAGGCAGAAC CACAGAATGA	TGGTTGAGGC TTGCCTACTT	-761 to +60	0.82	TACTGAATTAG	-129
Vip	ENSMUST000 00019906	CTCGAGCCTG GAATTAAGCC	CTCGAGCTAG GGAAGGCTCC	-1301 to +80	0.91	AATTTAATAAG	-127

		ACAGGA	ACCAGT				
Vipr2	ENSMUST000 00011315	CTCGAGACAA ATGTGCAGGT GGATCA	CTCGAGGCGG ATTTCCTCAGT CTCG	-1115 to +48	0.77	CAAGTAAGCAG	-323
